# Supplementary material for: Inference for Convolutionally Observed Diffusion Processes
Source: Entropy (Basel). 2020 Sep 15;22(9):1031. doi: 10.3390/e22091031 (PMC7597089; doi:10.3390/e22091031)
Supplement: Supplementary file 1 [file entropy-22-01031-s001.pdf]

SUPPLEMENTARY MATERIAL FOR "INFERENCE FOR CONVOLUTIONALLY OBSERVED DIFFUSION PROCESSES"

SHOGO H NAKAKITA[1,3,4] AND MASAYUKI UCHIDA[1,2]

Theorems and Lemmas in the Manuscript

Lemma 1. *Under [A1], we have the convergence in probability such that*

$$\frac{1}{nh_n}\sum_{2\le 2k\le n}\left(\overline{X}^{(i)}_{2kh_n,n}-\overline{X}^{(i)}_{(2k-2)h_n,n}\right)^2 \to^P \begin{cases}\nu_0\left(A^{(i,i)}\left(\cdot\right)\right) & \text{if } \rho_{\star}^{(i)}=0,\\ \nu_0\left(A^{(i,i)}\left(\cdot\right)\right)\left(1-\frac{\rho_{\star}^{(i)}}{6}\right) & \text{if } \rho_{\star}^{(i)}\in(0,2],\\ \nu_0\left(A^{(i,i)}\left(\cdot\right)\right)\left(\frac{2}{\rho_{\star}^{(i)}}-\frac{4}{3\left(\rho_{\star}^{(i)}\right)^2}\right) & \text{if } \rho_{\star}^{(i)}\in(2,\overline{\rho}].\end{cases}$$

Lemma 2. *R is a $\left[(3\overline{\rho}-1)(6\overline{\rho}-4)^{-1},1\right]$-valued monotonically decreasing continuous function, and has a continuous inverse $R^{-1}:\left[(3\overline{\rho}-1)(6\overline{\rho}-4)^{-1},1\right]\to[0,\overline{\rho}]$.*

Theorem 3. *Under [A1], $\hat{\rho}_n$ has consistency, i.e., $\hat{\rho}_n\to^P\rho_{\star}$.*

Theorem 4. *Under H_0 and [A1], we have the convergence in law such that*

$$\mathcal{T}_{i,n}\to^{\mathcal{L}}N(0,1).$$

We also obtain the result to support the consistency of the test.

Theorem 5. *Under H_1 and [A1], we have the convergence such that for any $c\in\mathbf{R}$,*

$$P\left(\mathcal{T}_{i,n}<c\right)\to 1.$$

Theorem 6. *Under [A1]-[A3], $\hat{\alpha}_n$ and $\hat{\beta}_n$ are consistent, i.e., $\hat{\alpha}_n\to^P\alpha_{\star}$ and $\hat{\beta}_n\to^P\beta_{\star}$.*

We have let p denote an integer such that $\sup_{n\in\mathbf{N}}ph_n\le\lambda$, $\Delta_n:=ph_n$. We set the sequence of the kernels $\left\{\Phi_{\Delta_n,n}\right\}_{n\in\mathbf{N}}$ such that $\Phi_{\Delta_n,n}\in\mathcal{K}\left(\Delta_n,d,M\right)$ for some $M>0$, $\int_0^{\Delta_n}\Phi_{\Delta_n,n}\mathrm{d}s=I_d$ and there exist a matrix $B\in\mathbf{R}^d\otimes\mathbf{R}^d$ such that

$$\left\|\int_0^{\Delta_n+h_n}\left(\Phi_{\Delta_n,n}\left((\Delta_n+h_n)-s\right)-\Phi_{\Delta_n,n}\left(\Delta_n-s\right)\right)s\mathrm{d}s-h_nB\right\|\le Ch_n^2\left(1+|x|\right)^C,$$

[1]Graduate School of Engineering Science, Osaka University
[2]Center for Mathematical Modeling and Data Science, Osaka University
[3]Japanese Society for the Promotion of Science
[4]The Ronin Institute for Independent Scholarship
Date: September 14, 2020.

a set $\mathbb{L} \subset \{0, \ldots, p\}$ such that there exist functions $D_\ell : \mathbf{R}^d \to \mathbf{R}^d \otimes \mathbf{R}^d$ for $\ell \in \mathbb{L}$ such that

$$\begin{aligned}&\Bigg\| \mathbf{E}\Bigg[\left(\int_0^{\Delta_n+(1+\ell)h_n} \Phi_{\Delta_n,n}\left(\Delta_n - s_1\right)\left(\int_0^{s_1} a\left(x\right)\mathrm{d}w_{s_2}\right)\mathrm{d}s_1\right)\\ &\quad\left(\int_0^{\Delta_n+(1+\ell)h_n} \left(\Phi_{\Delta_n,n}\left(\left(\Delta_n + \left(1+\ell\right)h_n\right) - s_1\right) - \Phi_{\Delta_n,n}\left(\Delta_n + \ell h_n - s_1\right)\right)\right.\\ &\qquad\qquad\left.\times\left(\int_0^{s_1} a\left(x\right)\mathrm{d}w_{s_2}\right)\mathrm{d}s_1\right)^T\Bigg] - h_n D_\ell\left(x\right)\Bigg\|\\ &\leq C h_n^2 \left(1+|x|\right)^C,\end{aligned}$$

a function $G : \mathbf{R}^d \to \mathbf{R}^d \otimes \mathbf{R}^d$ such that

$$\begin{aligned}&\Bigg\| \mathbf{E}\Bigg[\left(\int_0^{\Delta_n+h_n} \left(\Phi_{\Delta_n,n}\left(\left(\Delta_n + h_n\right) - s_1\right) - \Phi_{\Delta_n,n}\left(\Delta_n - s_1\right)\right)\left(\int_0^{s_1} a\left(x\right)\mathrm{d}w_{s_2}\right)\mathrm{d}s_1\right)\\ &\quad\left(\int_0^{\Delta_n+h_n} \left(\Phi_{\Delta_n,n}\left(\left(\Delta_n + h_n\right) - s_1\right) - \Phi_{\Delta_n,n}\left(\Delta_n - s_1\right)\right)\left(\int_0^{s_1} a\left(x\right)\mathrm{d}w_{s_2}\right)\mathrm{d}s_1\right)^T\Bigg]\\ &\quad - h_n G\left(x\right)\Bigg\|\\ &\leq C h_n^2 \left(1+|x|\right)^C,\end{aligned}$$

and we defined

$$\overline{X}_{t,n} = \int_{t-\Delta_n}^{t} \Phi_{\Delta_n,n}\left(t-s\right) X_s \mathrm{d}s,$$

and the following random quantities such that

$$\begin{aligned}\overline{\nu}_n\left(f\left(\cdot,\xi\right)\right) &:= \frac{1}{n}\sum_{i=1}^{n} f\left(\overline{X}_{ih_n,n},\xi\right),\\ \overline{I}_{\ell,n}\left(v\left(\cdot,\xi\right)\right) &:= \frac{1}{nh_n}\sum_{i=1+\ell}^{n} v\left(\overline{X}_{(i-1-\ell)h_n,n},\xi\right)\left[\overline{X}_{ih_n,n} - \overline{X}_{(i-1)h_n,n} - \left(h_n B\right) b\left(\overline{X}_{(i-1-\ell)h_n,n}\right)\right],\\ \overline{Q}_n\left(M\left(\cdot,\xi\right)\right) &:= \frac{1}{nh_n}\sum_{i=1}^{n} M\left(\overline{X}_{(i-1)h_n,n},\xi\right)\left[\left(\overline{X}_{ih_n,n} - \overline{X}_{(i-1)h_n,n}\right)^{\otimes 2}\right],\end{aligned}$$

where $f : \mathbf{R}^d \times \Xi \to \mathbf{R}$, $v : \mathbf{R}^d \times \Xi \to \mathbf{R}^d$, $M : \mathbf{R}^d \times \Xi \to \mathbf{R}^d \otimes \mathbf{R}^d$ are in $\mathcal{C}^2$-class, and their first and second derivatives and themselves are at most polynomial growth uniformly in $\xi \in \Xi$.

Proposition 7. *Under [A1],* $\overline{\nu}_n\left(f\left(\cdot,\xi\right)\right) \to^P \nu_0\left(f\left(\cdot,\xi\right)\right)$ *uniformly in* $\xi \in \Xi$.

Proposition 8. *If* $\ell \in \mathbb{L}$ *and [A1] hold,* $\overline{I}_{\ell,n}\left(v\left(\cdot,\xi\right)\right) \to^P \nu_0\left(\partial_x v\left[D_\ell^T\right]\left(\cdot,\xi\right)\right)$ *uniformly in* $\xi \in \Xi$.

Proposition 9. *Under [A1],* $\overline{Q}_n\left(M\left(\cdot,\xi\right)\right) \to^P \nu_0\left(M\left[G\right]\left(\cdot,\xi\right)\right)$ *uniformly in* $\xi \in \Xi$.

Appendix A. Proofs for preliminary lemmas and main results

Nonasymptotic results. We assume $\Delta \leq \lambda$, $k \in \mathbf{N}, M > 0$, and consider a class of $\overline{\mathbf{R}}^k \otimes \overline{\mathbf{R}}^d$-valued kernel functions on $\mathbf{R}$ denoted as $\mathcal{K}(\Delta, k, M)$ such that for all $\Phi_\Delta \in \mathcal{K}(\Delta, k, M)$, it holds:

(i) $\mathrm{supp}\Phi_\Delta \subset [0, \Delta]$,

(ii) for all $f : [0, \Delta] \times \Omega \to \mathbf{R}^k, \omega \in \Omega, \left|\int_0^\Delta \Phi_\Delta(\Delta - s) f(s, \omega)\,\mathrm{d}s\right| \leq M \sup_{s\in[0,\Delta]} |f(s, \omega)|$

(iii) for all $t_0 \geq -\lambda, f : \mathbf{R}^d \to \mathbf{R}$which is continuous and at most polynomial growth,

$$\mathbf{E}\left[\int_{t-\Delta}^t \Phi_\Delta(t-s) f(X_s)\,\mathrm{d}s \middle| \mathcal{F}_{t_0}\right] = \int_{t-\Delta}^t \Phi_\Delta(t-s)\,\mathbf{E}\left[f(X_s)\middle|\mathcal{F}_{t_0}\right]\mathrm{d}s.$$

Remark 1. Note one sufficient condition for $\Phi_\Delta \in \mathcal{K}(\Delta, k, M)$ is (i) $\Phi_\Delta : \mathbf{R} \to \mathbf{R}^k \otimes \mathbf{R}^d$, (ii) $\mathrm{supp}\Phi_\Delta \subset [0, \Delta]$, (iii) $\int_0^\Delta \|\Phi_\Delta(\Delta - s)\|\,\mathrm{d}s \leq M$ and (iv) $\mathcal{B}([0, \Delta])$-measurable since

$$\left|\int_0^\Delta \Phi_\Delta(\Delta - s) f(s, \omega)\,\mathrm{d}s\right| \leq \int_0^\Delta \|\Phi_\Delta(\Delta - s)\|\,|f(s, \omega)|\,\mathrm{d}s \leq M \sup_{s\in[0,\Delta]} |f(s, \omega)|$$

for Cauchy-Schwarz inequality, and Fubini's theorem.

It is easily checked that $V_{\rho,h_n} \in \mathcal{K}(\max_{i=1,\ldots,d} \rho_i h_n, d, d)$.

The next theorem is a generalisation of Proposition 2.2 of Gloter (2000), Theorem 1 of Gloter (2006) and Corollary 1 of Nakakita and Uchida (2019).

Theorem 10. *Set $t \geq 0$, $\Delta \in (0, \lambda]$, $k \in \mathbf{N}$, $M > 0$, $\Phi_\Delta, \Psi_\Delta \in \mathcal{K}(\Delta, k, M)$, and assume [A1]. Then we have*

$$\begin{aligned}\int_{t-\Delta}^t \Phi_\Delta(t-s) X_s \mathrm{d}s &= \left(\int_0^\Delta \Phi_\Delta(\Delta - s)\,\mathrm{d}s\right) X_{t-\Delta} + \left(\int_0^\Delta \Phi_\Delta(\Delta - s)\,s\mathrm{d}s\right) b(X_{t-\Delta}) \\ &\quad + \int_{t-\Delta}^t \Phi_\Delta(t-s_1)\left(\int_{t-\Delta}^{s_1} a(X_{t-\Delta})\,\mathrm{d}w_{s_2}\right)\mathrm{d}s_1 + e_{t-\Delta,\Delta},\end{aligned}$$

where $e_{t-\Delta,\Delta}$ is an $\mathbf{R}^k$-valued $\mathcal{F}_t$-measurable random variable such that

$$\text{(i)}\ \left|\mathbf{E}\left[e_{t-\Delta,\Delta}|\mathcal{F}_{t-\Delta}\right]\right| \leq C(M)\,\Delta^2\left(1 + |X_{t-\Delta}|\right)^{C(M)}, \tag{1}$$

$$\text{(ii) for all } m > 0,\ \mathbf{E}\left[|e_{t-\Delta,\Delta}|^m |\mathcal{F}_{t-\Delta}\right] \leq C(m, M)\,\Delta^m\left(1 + |X_{t-\Delta}|\right)^{C(m,M)}, \tag{2}$$

$$\begin{aligned}\text{(iii)}\ &\left|\mathbf{E}\left[e_{t-\Delta,\Delta}\left[\int_{t-\Delta}^t \Psi_\Delta(t-s_1)\left(\int_{t-\Delta}^{s_1} a(X_{t-\Delta})\,\mathrm{d}w_{s_2}\right)\mathrm{d}s_1\right]|\mathcal{F}_{t-\Delta}\right]\right| \\ &\leq C(M)\,\Delta^2\left(1 + |X_{t-\Delta}|\right)^{C(M)}.\end{aligned} \tag{3}$$

The following corollaries correspond more directly to Proposition 2.2 and Theorem 2.3 of Gloter (2000), or Proposition 1 and Theorem 1 of Gloter (2006).

Corollary 11. *Set $t \geq 0$, $\Delta \in (0, \lambda]$, $M > 0$ and $\Phi_\Delta \in \mathcal{K}(\Delta, d, M)$. We assume [A1] and $\int_0^\Delta \Phi_\Delta(\Delta - s)\,\mathrm{d}s = I_d$. Then we obtain*

$$\int_{t-\Delta}^t \Phi_\Delta(t-s) X_s \mathrm{d}s = X_{t-\Delta} + \int_{t-\Delta}^t \Phi_\Delta(t-s_1)\left(\int_{t-\Delta}^{s_1} a(X_{t-\Delta})\,\mathrm{d}w_{s_2}\right)\mathrm{d}s_1 + e_{t-\Delta,\Delta},$$

where $e_{t-\Delta,\Delta}$ *is an* $\mathbf{R}^d$*-valued* $\mathcal{F}_t$*-measurable random variable such that*

$$\text{(i)}\ \left|\mathbf{E}\left[e_{t-\Delta,\Delta}|\mathcal{F}_{t-\Delta}\right]\right| \leq C\left(M\right)\Delta\left(1+\left|X_{t-\Delta}\right|\right)^{C(M)}, \tag{4}$$

$$\text{(ii) for all } m>0,\ \mathbf{E}\left[\left|e_{t-\Delta,\Delta}\right|^m|\mathcal{F}_{t-\Delta}\right] \leq C\left(m,M\right)\Delta^m\left(1+\left|X_{t-\Delta}\right|\right)^{C(m,M)}. \tag{5}$$

Remark 2. This corollary leads to the same result as Proposition 2.2 of Gloter (2000) by setting $\Phi_\Delta(s) = I_d\left(\mathbf{1}_{[0,\Delta]}(s)/\Delta\right)$; we can see $\Phi_\Delta \in \mathcal{K}\left(\Delta, d, \sqrt{d}\right)$ because $\int_0^\Delta \|\Phi_\Delta(s)\|\,\mathrm{d}s = \sqrt{d}$. We have the following equalities

$$\int_0^\Delta \Phi_\Delta(s)\,\mathrm{d}s = I_d,$$

$$\begin{aligned}\int_{t-\Delta}^t \Phi_\Delta(t-s_1)\left(\int_{t-\Delta}^{s_1} a\left(X_{t-\Delta}\right)\mathrm{d}w_{s_2}\right)\mathrm{d}s_1 &= \int_{t-\Delta}^t\left(\int_{s_1}^t \Phi_\Delta(t-s_2)\,\mathrm{d}s_2\right)a\left(X_{t-\Delta}\right)\mathrm{d}w_{s_1}\\ &= \frac{1}{\Delta}a\left(X_{t-\Delta}\right)\int_{t-\Delta}^t (t-s_1)\,\mathrm{d}w_{s_1}.\end{aligned}$$

Then we can see that this result is identical to that of Gloter (2000).

Corollary 12. *Set* $t\geq 0$, $\Delta\in(0,\lambda]$, $M>0$, $\Phi_\Delta\in\mathcal{K}(\Delta,d,M)$, *and* $f(x,\xi)$ *is a real-valued function such that* $f:\mathbf{R}^d\times\Xi\to\mathbf{R}$, *and* f, $\partial_x f$ *and* $\partial_x^2 f$ *are polynomial growth uniformly in* $\xi\in\Xi$. *We assume [A1] and* $\int_0^\Delta \Phi_\Delta(\Delta-s)\,\mathrm{d}s = I_d$. *Then we obtain*

$$\begin{aligned}&\sup_{\xi\in\Xi}\left|\mathbf{E}\left[f\left(\int_{t-\Delta}^t \Phi_\Delta(t-s)X_s\mathrm{d}s,\xi\right)-f\left(X_{t-\Delta},\xi\right)|\mathcal{F}_{t-\Delta}\right]\right|\\ &\quad\leq C(M)\Delta\left(1+\left|X_{t-\Delta}\right|\right)^{C(M)},\\ &\mathbf{E}\left[\sup_{\xi\in\Xi}\left|f\left(\int_{t-\Delta}^t \Phi_\Delta(t-s)X_s\mathrm{d}s,\xi\right)-f\left(X_{t-\Delta},\xi\right)\right|^m|\mathcal{F}_{t-\Delta}\right]\\ &\quad\leq C(m,M)\Delta^{m/2}\left(1+\left|X_{t-\Delta}\right|\right)^{C(m,M)}\end{aligned}$$

for all $m>0$.

Remark 3. We see that this result generalises Proposition 4 of Nakakita and Uchida (2019); let us set $p\in\mathbf{N}$ and $h>0$ such that $ph\leq\lambda$, and Φ_{ph} as follows:

$$\Phi_{ph}(s) = \frac{1}{p}\sum_{i=0}^{p-1}\delta(ih-s)\,I_d.$$

It is obvious that $\Phi_{ph}\in\mathcal{K}(ph,d,d)$ and $\int_0^{ph}\Phi_{ph}(ph-s)\,\mathrm{d}s = I_d$.

Corollary 13. *Set* $t\geq 0$, $\Delta\in(0,\lambda]$, $k\in\mathbf{N}$, $M>0$ *and* $\Phi_\Delta\in\mathcal{K}(\Delta,k,M)$. *We assume that* $\int_0^\Delta\Phi_\Delta(\Delta-s)\,\mathrm{d}s = O$. *Then we obtain*

$$\begin{aligned}\int_{t-\Delta}^t \Phi_\Delta(t-s)X_s\mathrm{d}s &= \left(\int_0^\Delta \Phi_\Delta(\Delta-s)\,s\mathrm{d}s\right)b\left(X_{t-\Delta}\right)\\ &\quad+\int_{t-\Delta}^t \Phi_\Delta(t-s_1)\left(\int_{t-\Delta}^{s_1} a\left(X_{t-\Delta}\right)\mathrm{d}w_{s_2}\right)\mathrm{d}s_1 + e_{t-\Delta,\Delta},\end{aligned}$$

where $e_{t-\Delta,\Delta}$ is an $\mathbf{R}^k$-valued $\mathcal{F}_t$-measurable random variable such that

$$\text{(i)}\ \left|\mathbf{E}\left[e_{t-\Delta,\Delta}|\mathcal{F}_{t-\Delta}\right]\right| \le C\left(M\right)\Delta^2\left(1+\left|X_{t-\Delta}\right|\right)^{C(M)}, \tag{6}$$

$$\text{(ii) for all } m>0,\ \mathbf{E}\left[\left|e_{t-\Delta,\Delta}\right|^m|\mathcal{F}_{t-\Delta}\right] \le C\left(m,M\right)\Delta^m\left(1+\left|X_{t-\Delta}\right|\right)^{C(m,M)}, \tag{7}$$

$$\text{(iii)}\ \left|\mathbf{E}\left[e_{t-\Delta,\Delta}\left[\int_{t-\Delta}^{t}\Phi_\Delta\left(t-s_1\right)\left(\int_{t-\Delta}^{s_1}a\left(X_{t-\Delta}\right)\mathrm{d}w_{s_2}\right)\mathrm{d}s_1\right]|\mathcal{F}_{t-\Delta}\right]\right|^m$$
$$\le C\left(M\right)\Delta^2\left(1+\left|X_{t-\Delta}\right|\right)^{C(M)}. \tag{8}$$

Remark 4. We can have a result similar to Theorem 2.3 of Gloter (2000) if we assume that $\Delta'=\Delta/2$ and $\Phi_\Delta\left(s\right)=I_d\left(\mathbf{1}_{[0,\Delta']}\left(s\right)-\mathbf{1}_{[\Delta',\Delta]}\left(s\right)\right)/\Delta'$ where $\Delta\le\lambda$ and $k=d$. $\Phi_\Delta\left(s\right)\in\mathcal{K}\left(\Delta,d,2\sqrt{d}\right)$ because $\int_0^\Delta\left\|\Phi_\Delta\left(\Delta-s\right)\right\|\mathrm{d}s=2\sqrt{d}$. We have the following equalities

$$\begin{aligned}
&\int_0^\Delta\Phi_\Delta\left(\Delta-s\right)\mathrm{d}s=O,\\
&\int_0^\Delta\Phi_\Delta\left(\Delta-s\right)s\mathrm{d}s=I_d\left(\frac{1}{\Delta'}\int_{\Delta'}^{\Delta}s\mathrm{d}s-\frac{1}{\Delta'}\int_0^{\Delta'}s\mathrm{d}s\right)=I_d\left(\Delta-\frac{\Delta'}{2}-\frac{\Delta'}{2}\right)=\Delta' I_d,\\
&\int_{t-\Delta}^{t}\Phi_\Delta\left(t-s_1\right)\left(\int_{t-\Delta}^{s_1}a\left(X_{t-\Delta}\right)\mathrm{d}w_{s_2}\right)\mathrm{d}s_1\\
&\quad=\int_{t-\Delta}^{t}\left(\int_{s_1}^{t}\Phi_\Delta\left(t-s_2\right)\mathrm{d}s_2\right)a\left(X_{t-\Delta}\right)\mathrm{d}w_{s_1}\\
&\quad=\frac{1}{\Delta'}a\left(X_{t-\Delta}\right)\int_{t-\Delta}^{t}\left(\int_{s_1}^{t}\left(\mathbf{1}_{[0,\Delta']}\left(t-s_2\right)-\mathbf{1}_{[\Delta',\Delta]}\left(t-s_2\right)\right)\mathrm{d}s_2\right)\mathrm{d}w_{s_1}\\
&\quad=\frac{1}{\Delta'}a\left(X_{t-\Delta}\right)\int_{t-\Delta}^{t}\left(\Delta'\mathbf{1}_{[\Delta',\Delta]}\left(t-s_1\right)+\left(t-s_1\right)\mathbf{1}_{[0,\Delta']}\left(t-s_1\right)\right)\mathrm{d}w_{s_1}\\
&\qquad-\frac{1}{\Delta'}a\left(X_{t-\Delta}\right)\int_{t-\Delta}^{t}\left(t-\Delta'-s_1\right)\mathbf{1}_{[0,\Delta']}\left(t-\Delta'-s_1\right)\mathrm{d}w_{s_1}\\
&\quad=\frac{1}{\Delta'}a\left(X_{t-\Delta}\right)\left(\Delta'\int_{t-\Delta}^{t-\Delta'}\mathrm{d}w_s+\int_{t-\Delta'}^{t}\left(t-s_1\right)\mathrm{d}w_{s_1}\right)\\
&\qquad-\frac{1}{\Delta'}a\left(X_{t-\Delta}\right)\int_{t-\Delta}^{t-\Delta'}\left(t-\Delta'-s_1\right)\mathrm{d}w_{s_1}\\
&\quad=\frac{1}{\Delta'}a\left(X_{t-\Delta}\right)\left(\int_{t-\Delta}^{t-\Delta'}\left(s_1-\left(t-\Delta\right)\right)\mathrm{d}w_s+\int_{t-\Delta'}^{t}\left(t-s_1\right)\mathrm{d}w_{s_1}\right)
\end{aligned}$$

because of Lemma 14 in Appendix B. Hence this result and Corollary 12 give the same evaluation as Gloter (2000).

Remark 5. This corollary also generalises Corollary 1 of Nakakita and Uchida (2019) when we ignore noise term; let us set $p\in\mathbf{N}$ and $h>0$ such that $2ph\le\lambda$, and Φ_{2ph} as follows:

$$\Phi_{2ph}\left(s\right)=\frac{1}{p}\sum_{i=0}^{p-1}\delta\left(s-ih\right)I_d-\frac{1}{p}\sum_{i=0}^{p-1}\delta\left(s-\left(p+i\right)h\right)I_d.$$

It is obvious that $\Phi_{2ph} \in \mathcal{K}(2ph, d, 2d)$ and $\int_0^{2ph} \Phi_{2ph}(2ph - s)\,\mathrm{d}s = O$. We can evaluate

$$\int_0^{2ph} \Phi_{2ph}(2ph - s)\, s\mathrm{d}s = \frac{1}{p}\sum_{i=0}^{p-1}(2ph - ih)\, I_d - \frac{1}{p}\sum_{i=0}^{p-1}(2ph - (p+i)\,h)\, I_d = phI_d,$$

$$\begin{aligned}
&\int_{t-2ph}^{t} \Phi_{2ph}(t - s_1)\left(\int_{t-2ph}^{s_1} a(X_{t-2ph})\,\mathrm{d}w_{s_2}\right)\mathrm{d}s_1 \\
&= a(X_{t-2ph})\left(\frac{1}{p}\sum_{i=0}^{p-1}\int_{t-2ph}^{t-ih}\mathrm{d}w_s - \frac{1}{p}\sum_{i=0}^{p-1}\int_{t-2ph}^{t-(p+i)h}\mathrm{d}w_s\right) \\
&= a(X_{t-2ph})\left(\frac{1}{p}\sum_{i=0}^{p-1}(i+1)\int_{t-(2p-i)h}^{t-(2p-i+1)h}\mathrm{d}w_s + \frac{1}{p}\sum_{i=0}^{p-1}(p-1-i)\int_{t-(p-i)h}^{t-(p-i+1)h}\mathrm{d}w_s\right).
\end{aligned}$$

Proof of Theorem 10. We have

$$\begin{aligned}
&\int_{t-\Delta}^{t}\Phi_\Delta(t-s)\,X_s\mathrm{d}s \\
&= \int_{t-\Delta}^{t}\Phi_\Delta(t-s_1)\left(X_{t-\Delta} + \int_{t-\Delta}^{s_1} b(X_{s_2})\,\mathrm{d}s_2 + \int_{t-\Delta}^{s_1} a(X_{s_2})\,\mathrm{d}w_{s_2}\right)\mathrm{d}s_1 \\
&= \left(\int_{t-\Delta}^{t}\Phi_\Delta(t-s_1)\,\mathrm{d}s\right)X_{t-\Delta} + \int_{t-\Delta}^{t}\Phi_\Delta(t-s_1)\left(\int_{t-\Delta}^{s_1} b(X_{t-\Delta})\,\mathrm{d}s_2\right)\mathrm{d}s_1 \\
&\quad + \int_{t-\Delta}^{t}\Phi_\Delta(t-s_1)\left(\int_{t-\Delta}^{s_1} a(X_{t-\Delta})\,\mathrm{d}w_{s_2}\right)\mathrm{d}s_1 \\
&\quad + \int_{t-\Delta}^{t}\Phi_\Delta(t-s_1)\left(\int_{t-\Delta}^{s_1} (b(X_{s_2}) - b(X_{t-\Delta}))\,\mathrm{d}s_2\right)\mathrm{d}s_1 \\
&\quad + \int_{t-\Delta}^{t}\Phi_\Delta(t-s_1)\left(\int_{t-\Delta}^{s_1} (a(X_{s_2}) - a(X_{t-\Delta}))\,\mathrm{d}w_{s_2}\right)\mathrm{d}s_1.
\end{aligned}$$

Set $e_{t-\Delta,\Delta} := e_{t-\Delta,\Delta,1} + e_{t-\Delta,\Delta,2}$ where

$$\begin{aligned}
e_{t-\Delta,\Delta,1} &:= \int_{t-\Delta}^{t}\Phi_\Delta(t-s_1)\left(\int_{t-\Delta}^{s_1}(b(X_{s_2}) - b(X_{t-\Delta}))\,\mathrm{d}s_2\right)\mathrm{d}s_1, \\
e_{t-\Delta,\Delta,2} &:= \int_{t-\Delta}^{t}\Phi_\Delta(t-s_1)\left(\int_{t-\Delta}^{s_1}(a(X_{s_2}) - a(X_{t-\Delta}))\,\mathrm{d}w_{s_2}\right)\mathrm{d}s_1.
\end{aligned}$$

We examine that the properties (i)-(iii) hold for this $e_{t-\Delta,\Delta}$. The assumption of Φ_Δ and martingale property of stochastic integral verify

$$\mathbf{E}\left[e_{t-\Delta,\Delta,2}|\mathcal{F}_{t-\Delta}\right] = \mathbf{0}.$$

Then, in order to show (i) and (ii), it is sufficient to prove the following inequalities.

$$\begin{aligned}
\left|\mathbf{E}\left[e_{t-\Delta,\Delta,1}|\mathcal{F}_{t-\Delta}\right]\right| &\le C(M)\,\Delta^2\,(1 + |X_{t-\Delta}|)^{C(M)}, \\
\mathbf{E}\left[|e_{t-\Delta,\Delta,1}|^m\,|\mathcal{F}_{t-\Delta}\right] &\le C(m,M)\,\Delta^{3m/2}\,(1 + |X_{t-\Delta}|)^{C(m,M)}, \\
\mathbf{E}\left[|e_{t-\Delta,\Delta,2}|^m\,|\mathcal{F}_{t-\Delta}\right] &\le C(m,M)\,\Delta^{m}\,(1 + |X_{t-\Delta}|)^{C(m,M)}.
\end{aligned}$$

In the first place, we have

$$\begin{aligned}
&\left|\mathbf{E}\left[\int_{t-\Delta}^{t}\Phi_{\Delta}\left(t-s_1\right)\left(\int_{t-\Delta}^{s_1}\left(b\left(X_{s_2}\right)-b\left(X_{t-\Delta}\right)\right)\mathrm{d}s_2\right)\mathrm{d}s_1|\mathcal{F}_{t-\Delta}\right]\right|\\
&=\left|\int_{t-\Delta}^{t}\mathbf{E}\left[\Phi_{\Delta}\left(t-s_1\right)\left(\int_{t-\Delta}^{s_1}\left(b\left(X_{s_2}\right)-b\left(X_{t-\Delta}\right)\right)\mathrm{d}s_2\right)|\mathcal{F}_{t-\Delta}\right]\mathrm{d}s_1\right|\\
&=\left|\int_{t-\Delta}^{t}\Phi_{\Delta}\left(t-s_1\right)\mathbf{E}\left[\int_{t-\Delta}^{s_1}\left(b\left(X_{s_2}\right)-b\left(X_{t-\Delta}\right)\right)\mathrm{d}s_2|\mathcal{F}_{t-\Delta}\right]\mathrm{d}s_1\right|\\
&=\left|\int_{t-\Delta}^{t}\Phi_{\Delta}\left(t-s_1\right)\left(\int_{t-\Delta}^{s_1}\mathbf{E}\left[b\left(X_{s_2}\right)-b\left(X_{t-\Delta}\right)|\mathcal{F}_{t-\Delta}\right]\mathrm{d}s_2\right)\mathrm{d}s_1\right|\\
&\leq C\left(M\right)\sup_{s_1\in[t-\Delta,t]}\left|\int_{t-\Delta}^{s_1}\mathbf{E}\left[b\left(X_{s_2}\right)-b\left(X_{t-\Delta}\right)|\mathcal{F}_{t-\Delta}\right]\mathrm{d}s_2\right|\\
&\leq C\left(M\right)\sup_{s_1\in[t-\Delta,t]}\int_{t-\Delta}^{s_1}\left|\mathbf{E}\left[b\left(X_{s_2}\right)-b\left(X_{t-\Delta}\right)|\mathcal{F}_{t-\Delta}\right]\right|\mathrm{d}s_2\\
&\leq C\left(M\right)\Delta\sup_{s\in[t-\Delta,t]}\left|\mathbf{E}\left[b\left(X_{s}\right)-b\left(X_{t-\Delta}\right)|\mathcal{F}_{t-\Delta}\right]\right|\\
&\leq C\left(M\right)\Delta^2\left(1+\left|X_{t-\Delta}\right|\right)^{C(M)}
\end{aligned}$$

because of Proposition A of Gloter (2000). Secondly, we obtain

$$\begin{aligned}
&\left|\int_{t-\Delta}^{t}\Phi_{\Delta}\left(t-s_1\right)\left(\int_{t-\Delta}^{s_1}\left(b\left(X_{s_2}\right)-b\left(X_{t-\Delta}\right)\right)\mathrm{d}s_2\right)\mathrm{d}s_1\right|\\
&\leq C\left(M\right)\sup_{s_1\in[t-\Delta,t]}\left|\int_{t-\Delta}^{s_1}\left(b\left(X_{s_2}\right)-b\left(X_{t-\Delta}\right)\right)\mathrm{d}s_2\right|\\
&\leq C\left(M\right)\sup_{s_1\in[t-\Delta,t]}\int_{t-\Delta}^{s_1}\left|b\left(X_{s_2}\right)-b\left(X_{t-\Delta}\right)\right|\mathrm{d}s_2\\
&\leq C\left(M\right)\Delta\sup_{s\in[t-\Delta,t]}\left|b\left(X_{s}\right)-b\left(X_{t-\Delta}\right)\right|
\end{aligned}$$

by $\Phi_{\Delta}\in\mathcal{K}\left(\Delta,k,M\right)$; therefore, we obtain

$$\begin{aligned}
&\mathbf{E}\left[\left|\int_{t-\Delta}^{t}\Phi_{\Delta}\left(t-s_1\right)\left(\int_{t-\Delta}^{s_1}\left(b\left(X_{s_2}\right)-b\left(X_{t-\Delta}\right)\right)\mathrm{d}s_2\right)\mathrm{d}s_1\right|^m|\mathcal{F}_{t-\Delta}\right]\\
&\leq C\left(m,M\right)\Delta^m\mathbf{E}\left[\sup_{s\in[t-\Delta,t]}\left|b\left(X_{s}\right)-b\left(X_{t-\Delta}\right)\right|^m|\mathcal{F}_{t-\Delta}\right]\\
&\leq C\left(m,M\right)\Delta^{3m/2}\left(1+\left|X_{t-\Delta}\right|\right)^{C(m,M)}
\end{aligned}$$

due to Proposition 5.1 of Gloter (2000). Thirdly, by Hölder's inequality and the Burkholder-Davis-Gundy one, and Proposition A of Gloter (2000),

$$\begin{aligned}
&\mathbf{E}\left[\left|\int_{t-\Delta}^{t}\Phi_{\Delta}\left(t-s_1\right)\left(\int_{t-\Delta}^{s_1}\left(a\left(X_{s_2}\right)-a\left(X_{t-\Delta}\right)\right)\mathrm{d}w_{s_2}\right)\mathrm{d}s_1\right|^m|\mathcal{F}_{t-\Delta}\right]\\
&\leq C\left(m,M\right)\mathbf{E}\left[\left|\sup_{s_1\in[t-\Delta,t]}\int_{t-\Delta}^{s_1}\left(a\left(X_{s_2}\right)-a\left(X_{t-\Delta}\right)\right)\mathrm{d}w_{s_2}\right|^m|\mathcal{F}_{t-\Delta}\right]
\end{aligned}$$

$$\leq C(m,M)\mathbf{E}\left[\left|\int_{t-\Delta}^{t}\left\|a(X_{s_2})-a(X_{t-\Delta})\right\|^2\mathrm{d}s_2\right|^{m/2}|\mathcal{F}_{t-\Delta}\right]$$
$$\leq C(m,M)\Delta^{m/2}\sup_{s\in[t-\Delta,t]}\mathbf{E}\left[\left\|a(X_s)-a(X_{t-\Delta})\right\|^m|\mathcal{F}_{t-\Delta}\right]$$
$$\leq C(m,M)\Delta^{m}\left(1+|X_{t-\Delta}|\right)^{C(m,M)}.$$

To show (iii), it is obvious that

$$\left|\mathbf{E}\left[e_{t-\Delta,\Delta,1}\left[\int_{t-\Delta}^{t}\Phi_\Delta(t-s_1)\left(\int_{t-\Delta}^{s_1}a(X_{t-\Delta})\mathrm{d}w_{s_2}\right)\mathrm{d}s_1\right]|\mathcal{F}_{t-\Delta}\right]\right|$$
$$\leq C(M)\Delta^2\left(1+|X_{t-\Delta}|\right)^{C(M)}$$

due to Hölder's inequality and the evaluation analogous to $\mathbf{E}\left[|e_{t-\Delta,\Delta,2}|^m|\mathcal{F}_{t-\Delta}\right]$ such that

$$\mathbf{E}\left[\left|\int_{t-\Delta}^{t}\Phi_\Delta(t-s_1)\left(\int_{t-\Delta}^{s_1}a(X_{t-\Delta})\mathrm{d}w_{s_2}\right)\mathrm{d}s_1\right|^m|\mathcal{F}_{t-\Delta}\right]$$
$$\leq C(m,M)\Delta^{m/2}\left(1+|X_{t-\Delta}|\right)^{C(m,M)}.$$

Hence it is sufficient to show

$$\left|\mathbf{E}\left[e_{t-\Delta,\Delta,2}\left[\int_{t-\Delta}^{t}\Phi_\Delta(t-s_1)\left(\int_{t-\Delta}^{s_1}a(X_{t-\Delta})\mathrm{d}w_{s_2}\right)\mathrm{d}s_1\right]|\mathcal{F}_{t-\Delta}\right]\right|$$
$$\leq C(M)\Delta^2\left(1+|X_{t-\Delta}|\right)^{C(M)}.$$

We can evaluate the left hand side such that

$$\left|\mathbf{E}\left[\int_{t-\Delta}^{t}\Phi_\Delta(t-s_1)\left(\int_{t-\Delta}^{s_1}\left(a(X_{s_2})-a(X_{t-\Delta})\right)\mathrm{d}w_{s_2}\right)\mathrm{d}s_1\right.\right.$$
$$\left.\left.\left[\int_{t-\Delta}^{t}\Psi_\Delta(t-s_1')\left(\int_{t-\Delta}^{s_1'}a(X_{t-\Delta})\mathrm{d}w_{s_2'}\right)\mathrm{d}s_1'\right]|\mathcal{F}_{t-\Delta}\right]\right|$$
$$=\left|\int_{t-\Delta}^{t}\Phi_\Delta(t-s_1)\mathbf{E}\left[\left(\int_{t-\Delta}^{s_1}\left(a(X_{s_2})-a(X_{t-\Delta})\right)\mathrm{d}w_{s_2}\right)\right.\right.$$
$$\left.\left.\left[\int_{t-\Delta}^{t}\Psi_\Delta(t-s_1')\left(\int_{t-\Delta}^{s_1'}a(X_{t-\Delta})\mathrm{d}w_{s_2'}\right)\mathrm{d}s_1'\right]|\mathcal{F}_{t-\Delta}\right]\mathrm{d}s_1\right|$$
$$\leq C(M)\sup_{s_1\in[t-\Delta,t]}\left|\mathbf{E}\left[\left(\int_{t-\Delta}^{s_1}\left(a(X_{s_2})-a(X_{t-\Delta})\right)\mathrm{d}w_{s_2}\right)\right.\right.$$
$$\left.\left.\left[\int_{t-\Delta}^{t}\Psi_\Delta(t-s_1')\left(\int_{t-\Delta}^{s_1'}a(X_{t-\Delta})\mathrm{d}w_{s_2'}\right)\mathrm{d}s_1'\right]|\mathcal{F}_{t-\Delta}\right]\right|$$
$$=C(M)\sup_{s_1\in[t-\Delta,t]}\left|\int_{t-\Delta}^{t}\Psi_\Delta(t-s_1')\mathbf{E}\left[\left(\int_{t-\Delta}^{s_1}\left(a(X_{s_2})-a(X_{t-\Delta})\right)\mathrm{d}w_{s_2}\right)\right.\right.$$
$$\left.\left.\left[\int_{t-\Delta}^{s_1'}a(X_{t-\Delta})\mathrm{d}w_{s_2'}\right]|\mathcal{F}_{t-\Delta}\right]\mathrm{d}s_1'\right|$$
$$\leq C(M)$$

$$
\begin{aligned}
&\times \sup_{s_1\in[t-\Delta,t]}\sup_{s_1'\in[t-\Delta,t]}\left|\mathbf{E}\left[\int_{t-\Delta}^{s_1}\left(a\left(X_{s_2}\right)-a\left(X_{t-\Delta}\right)\right)\mathrm{d}w_{s_2}\left[\int_{t-\Delta}^{s_1'}a\left(X_{t-\Delta}\right)\mathrm{d}w_{s_2'}\right]|\mathcal{F}_{t-\Delta}\right]\right|\\
&\le C\left(M\right)\sup_{s_1\in[t-\Delta,t]}\left|\mathbf{E}\left[\int_{t-\Delta}^{s_1}\left(a\left(X_{s_2}\right)-a\left(X_{t-\Delta}\right)\right)\mathrm{d}w_{s_2}\left[\int_{t-\Delta}^{s_1}a\left(X_{t-\Delta}\right)\mathrm{d}w_{s_2'}\right]|\mathcal{F}_{t-\Delta}\right]\right|\\
&\le C\left(M\right)\sup_{s_1\in[t-\Delta,t]}\left|\mathbf{E}\left[\int_{t-\Delta}^{s_1}\left(a\left(X_{s_2}\right)-a\left(X_{t-\Delta}\right)\right)\mathrm{d}w_{s_2}\left[\int_{t-\Delta}^{s_1}a\left(X_{t-\Delta}\right)\mathrm{d}w_{s_2'}\right]|\mathcal{F}_{t-\Delta}\right]\right|\\
&= C\left(M\right)\sup_{s_1\in[t-\Delta,t]}\left|\mathbf{E}\left[\int_{t-\Delta}^{s_1}\left(a\left(X_{s_2}\right)-a\left(X_{t-\Delta}\right)\right)\mathrm{d}s_2\left[a\left(X_{t-\Delta}\right)\right]|\mathcal{F}_{t-\Delta}\right]\right|\\
&= C\left(M\right)\sup_{s_1\in[t-\Delta,t]}\left|\int_{t-\Delta}^{s_1}\mathbf{E}\left[\left(a\left(X_{s_2}\right)-a\left(X_{t-\Delta}\right)\right)|\mathcal{F}_{t-\Delta}\right]\mathrm{d}s_2\left[a\left(X_{t-\Delta}\right)\right]\right|\\
&\le C\left(M\right)\Delta\sup_{s\in[t-\Delta,t]}\left\|\mathbf{E}\left[\left(a\left(X_s\right)-a\left(X_{t-\Delta}\right)\right)|\mathcal{F}_{t-\Delta}\right]\right\|\left\|a\left(X_{t-\Delta}\right)\right\|\\
&\le C\left(M\right)\Delta^2\left(1+\left|X_{t-\Delta}\right|\right)^{C(M)}.
\end{aligned}
$$

Hence we obtain the proof of (iii). □

Proof of Corollary 11. It follows from Theorem 10 directly. □

Proof of Corollary 12. By Taylor's expansion,

$$
\begin{aligned}
&f\left(\int_{t-\Delta}^{t}\Phi_\Delta\left(t-s\right)X_s\mathrm{d}s,\xi\right)-f\left(X_{t-\Delta},\xi\right)\\
&=\partial_x f\left(X_{t-\Delta},\xi\right)\left(\int_{t-\Delta}^{t}\Phi_\Delta\left(t-s\right)X_s\mathrm{d}s-X_{t-\Delta}\right)\\
&\quad+\int_0^1\left(1-u\right)\partial_x^2 f\left(X_{t-\Delta}+u\left(\int_{t-\Delta}^{t}\Phi_\Delta\left(t-s\right)X_s\mathrm{d}s-X_{t-\Delta}\right),\xi\right)\mathrm{d}u\\
&\qquad\left[\left(\int_{t-\Delta}^{t}\Phi_\Delta\left(t-s\right)X_s\mathrm{d}s-X_{t-\Delta}\right)^{\otimes 2}\right].
\end{aligned}
$$

It is obvious that

$$
\begin{aligned}
&\sup_{\xi\in\Xi}\left|\mathbf{E}\left[\partial_x f\left(X_{t-\Delta},\xi\right)\left(\int_{t-\Delta}^{t}\Phi_\Delta\left(t-s\right)X_s\mathrm{d}s-X_{t-\Delta}\right)|\mathcal{F}_{t-\Delta}\right]\right|\\
&\le\sup_{\xi\in\Xi}\left|\partial_x f\left(X_{t-\Delta},\xi\right)\right|\left|\mathbf{E}\left[\int_{t-\Delta}^{t}\Phi_\Delta\left(t-s\right)X_s\mathrm{d}s-X_{t-\Delta}|\mathcal{F}_{t-\Delta}\right]\right|\\
&\le C\left(M\right)\left(1+\left|X_{t-\Delta}\right|\right)^{C(M)}\left|\mathbf{E}\left[e_{t-\Delta,\Delta}|\mathcal{F}_{t-\Delta}\right]\right|\\
&\le C\left(M\right)\Delta\left(1+\left|X_{t-\Delta}\right|\right)^{C(M)}
\end{aligned}
$$

by Corollary 11. We also have

$$\begin{aligned}
&\sup_{\xi\in\Xi}\left|\mathbf{E}\left[\int_0^1(1-u)\,\partial_x^2 f\left(X_{t-\Delta}+u\left(\int_{t-\Delta}^t\Phi_\Delta(t-s)\,X_s\mathrm{d}s-X_{t-\Delta}\right),\xi\right)\mathrm{d}u\right.\right.\\
&\qquad\qquad\left.\left.\left[\left(\int_{t-\Delta}^t\Phi_\Delta(t-s)\,X_s\mathrm{d}s-X_{t-\Delta}\right)^{\otimes 2}\right]|\mathcal{F}_{t-\Delta}\right]\right|\\
&\le\sup_{\xi\in\Xi}\mathbf{E}\left[\left\|\int_0^1(1-u)\,\partial_x^2 f\left(X_{t-\Delta}+u\left(\int_{t-\Delta}^t\Phi_\Delta(t-s)\,X_s\mathrm{d}s-X_{t-\Delta}\right),\xi\right)\mathrm{d}u\right\|\right.\\
&\qquad\qquad\left.\left|\int_{t-\Delta}^t\Phi_\Delta(t-s)\,X_s\mathrm{d}s-X_{t-\Delta}\right|^2|\mathcal{F}_{t-\Delta}\right]\\
&\le\mathbf{E}\left[C\left(1+|X_{t-\Delta}|+\left|\int_{t-\Delta}^t\Phi_\Delta(t-s)\,X_s\mathrm{d}s\right|\right)^C|\mathcal{F}_{t-\Delta}\right]^{1/2}\\
&\quad\times\mathbf{E}\left[\left|\int_{t-\Delta}^t\Phi_\Delta(t-s)\,X_s\mathrm{d}s-X_{t-\Delta}\right|^4|\mathcal{F}_{t-\Delta}\right]^{1/2}\\
&\le\mathbf{E}\left[C\left(1+\sup_{s\in[t-\Delta,t]}|X_s|\right)^C|\mathcal{F}_{t-\Delta}\right]^{1/2}C(M)\,\Delta\,(1+|X_{t-\Delta}|)^{C(M)}\\
&\le C(M)\,\Delta\,(1+|X_{t-\Delta}|)^{C(M)}
\end{aligned}$$

because of Corollary 11 and Proposition 5.1 of Gloter (2000). Here we obtain the first evaluation. With respect to the second one, we can have the following evaluation as above:

$$\begin{aligned}
&\mathbf{E}\left[\sup_{\xi\in\Xi}\left|f\left(\int_{t-\Delta}^t\Phi_\Delta(t-s)\,X_s\mathrm{d}s,\xi\right)-f(X_{t-\Delta},\xi)\right|^m|\mathcal{F}_{t-\Delta}\right]\\
&\le\mathbf{E}\left[C(m)\left(1+\sup_{s\in[t-\Delta,t]}|X_s|\right)^{C(m)}|\mathcal{F}_{t-\Delta}\right]^{\frac{1}{2}}\\
&\qquad\qquad\times\mathbf{E}\left[\left|\int_{t-\Delta}^t\Phi_\Delta(t-s)\,X_s\mathrm{d}s-X_{t-\Delta}\right|^{2m}|\mathcal{F}_{t-\Delta}\right]^{\frac{1}{2}}\\
&\le C(m,M)\,\Delta^{m/2}\,(1+|X_{t-\Delta}|)^{C(m,M)}.
\end{aligned}$$

Hence the proof is complete. □

Proof of Corollary 13. It follows from Theorem 10 directly as Corollary 11. □

Proofs of the results for some laws of large numbers.

General results.

Remark 6. When $\Phi_{\Delta_n,n}(s) = \frac{1}{h_n}\mathbf{1}_{[0,h_n]}(s) I_d$, $p=1$, $M=1$, then

$$\begin{aligned}\int_0^{2h_n} \left(\Phi_{\Delta_n,n}(2h_n - s) - \Phi_{\Delta_n,n}(h_n - s)\right) s\mathrm{d}s &= \frac{1}{h_n} I_d \int_{h_n}^{2h_n} s\mathrm{d}s - \frac{1}{h_n} I_d \int_0^{h_n} s\mathrm{d}s \\ &= \frac{1}{h_n} I_d \left[\frac{(2h_n)^2 - 2(h_n)^2}{2}\right] \\ &= h_n I_d\end{aligned}$$

and hence we obtain $B = I_d$; we also can evaluate $D_0(x) = \frac{1}{6}A(x)$, which coincides with that of Gloter (2006).

Proof of Proposition 7. It is obvious by Corollary 12 and the assumption for f. □

Proof of Proposition 8. We decompose the summation as follows:

$$\begin{aligned}&\bar{I}_{\ell,n}(v(\cdot,\xi)) \\ &= \frac{1}{nh_n}\sum_{i=1+\ell}^{n} v\left(\bar{X}_{(i-1-\ell)h_n,n},\xi\right)\left[\bar{X}_{ih_n,n} - \bar{X}_{(i-1)h_n,n} - (h_nB)\, b\left(\bar{X}_{(i-1-\ell)h_n,n}\right)\right] \\ &= \frac{1}{nh_n}\sum_{i=1+\ell}^{n} v\left(\bar{X}_{(i-1-\ell)h_n,n},\xi\right)\left[\bar{X}_{ih_n,n} - \bar{X}_{(i-1)h_n,n} - (h_nB)\, b\left(X_{(i-2p-1)h_n}\right)\right] \\ &\quad + \frac{1}{nh_n}\sum_{i=1+\ell}^{n} v\left(\bar{X}_{(i-1-\ell)h_n,n},\xi\right)\left[(h_nB)\, b\left(X_{(i-2p-1)h_n}\right) - (h_nB)\, b\left(\bar{X}_{(i-1-\ell)h_n,n}\right)\right] \\ &= \frac{1}{nh_n}\sum_{i=1+\ell}^{n} v\left(X_{(i-2p-1)h_n},\xi\right)\left[\bar{X}_{ih_n,n} - \bar{X}_{(i-1)h_n,n} - (h_nB)\, b\left(X_{(i-2p-1)h_n}\right)\right] \\ &\quad + \frac{1}{nh_n}\sum_{i=1+\ell}^{n} \partial_x v\left(X_{(i-2p-1)h_n},\xi\right)\left[\left(\bar{X}_{ih_n,n} - \bar{X}_{(i-1)h_n,n} - (h_nB)\, b\left(X_{(i-2p-1)h_n}\right)\right)\right. \\ &\qquad\qquad \left.\left(\bar{X}_{(i-1-\ell)h_n,n} - X_{(i-2p-1)h_n}\right)^T\right] \\ &\quad + \frac{1}{nh_n}\sum_{i=1+\ell}^{n}\sum_{j_1=1}^{d}\sum_{j_2=1}^{d} \\ &\qquad \int_0^1 (1-s)\,\partial_{x^{(j_1)}}\partial_{x^{(j_2)}} v\left(X_{(i-p-1)h_n} + s\left(\bar{X}_{(i-1)h_n,n} - X_{(i-p-1)h_n}\right),\xi\right)\mathrm{d}s \\ &\qquad \left(\bar{X}_{(i-1-\ell)h_n,n} - X_{(i-2p-1)h_n}\right)^{(j_1)}\left(\bar{X}_{(i-1-\ell)h_n,n} - X_{(i-2p-1)h_n}\right)^{(j_2)} \\ &\qquad \left[\bar{X}_{ih_n,n} - \bar{X}_{(i-1)h_n,n} - (h_nB)\, b\left(X_{(i-2p-1)h_n}\right)\right] \\ &\quad + \frac{1}{nh_n}\sum_{i=1+\ell}^{n} v\left(\bar{X}_{(i-1-\ell)h_n,n},\xi\right)\left[(h_nB)\, b\left(X_{(i-2p-1)h_n}\right) - (h_nB)\, b\left(\bar{X}_{(i-1-\ell)h_n,n}\right)\right].\end{aligned}$$

Because of the evaluation such that

$$\frac{1}{nh_n}\sum_{1+\ell\le(2p+1)i\le n}\left|\mathbf{E}\left[v\left(X_{(2p+1)(i-1)h_n},\xi\right)\left[\overline{X}_{(2p+1)ih_n,n}-\overline{X}_{((2p+1)i-1)h_n,n}-(h_nB)\,b\left(X_{(2p+1)(i-1)h_n}\right)\right]\middle|\mathcal{F}_{(2p+1)(i-1)h_n}\right]\right| \to^P 0,$$

$$\frac{1}{n^2h_n^2}\sum_{1+\ell\le(2p+1)i\le n}\mathbf{E}\left[\left|v\left(X_{(2p+1)(i-1)h_n},\xi\right)\left[\overline{X}_{(2p+1)ih_n,n}-\overline{X}_{((2p+1)i-1)h_n,n}-(h_nB)\,b\left(X_{(2p+1)(i-1)h_n}\right)\right]\right|^2\middle|\mathcal{F}_{(2p+1)(i-1)h_n}\right] \to^P 0$$

for all $\xi\in\Xi$ and Lemma 9 of Genon-Catalot and Jacod (1993), we have

$$\frac{1}{nh_n}\sum_{i=1+\ell}^{n}v\left(X_{(i-2p-1)h_n},\xi\right)\left[\overline{X}_{ih_n,n}-\overline{X}_{(i-1)h_n,n}-(h_nB)\,b\left(X_{(i-2p-1)h_n}\right)\right]\to^P 0$$

for all $\xi\in\Xi$. To verify the uniform convergence in probability of this summation, we show that the following inequalities hold (Ibragimov and Has'minskii, 1981): there exist $C>0$ and $k>\dim\Xi$ such that for all $n\in\mathbf{N}$ and $\xi,\xi'\in\Xi$,

$$\mathbf{E}\left[\left|\frac{1}{nh_n}\sum_{i=1+\ell}^{n}v\left(X_{(i-2p-1)h_n},\xi\right)\left[\overline{X}_{ih_n,n}-\overline{X}_{(i-1)h_n,n}-(h_nB)\,b\left(X_{(i-2p-1)h_n}\right)\right]\right|^k\right]\le C,$$

$$\mathbf{E}\left[\left|\frac{1}{nh_n}\sum_{i=1+\ell}^{n}v\left(X_{(i-2p-1)h_n},\xi\right)\left[\overline{X}_{ih_n,n}-\overline{X}_{(i-1)h_n,n}-(h_nB)\,b\left(X_{(i-2p-1)h_n}\right)\right]-\frac{1}{nh_n}\sum_{i=1+\ell}^{n}v\left(X_{(i-2p-1)h_n},\xi'\right)\left[\overline{X}_{ih_n,n}-\overline{X}_{(i-1)h_n,n}-(h_nB)\,b\left(X_{(i-2p-1)h_n}\right)\right]\right|^k\right]\le C\left|\xi-\xi'\right|^k.$$

These evaluations can be led by the assumption of Φ_Δ and Burkholder's inequality in a similar way to Nakakita and Uchida (2019).

With respect to the second summation, we can easily have the evaluation such that

$$\begin{aligned}&\frac{1}{nh_n}\sum_{1+\ell\le(2p+1)i\le n}\partial_x v\left(X_{(2p+1)(i-1)h_n},\xi\right)\\&\qquad\left[\left(\overline{X}_{(2p+1)ih_n,n}-\overline{X}_{((1p+1)i-1)h_n,n}-(h_nB)\,b\left(X_{(2p+1)(i-1)h_n}\right)\right)\right.\\&\qquad\left.\left(\overline{X}_{((2p+1)i-1-\ell)h_n,n}-X_{(2p+1)(i-1)h_n}-\left(\int_0^{\Delta_n}\Phi_{\Delta_n,n}\left(\Delta_n-s\right)\mathrm{sd}s\right)b\left(X_{(p+1)(i-1)}\right)\right)^T\right]\\&+\frac{1}{nh_n}\sum_{1+\ell\le(2p+1)i\le n}\partial_x v\left(X_{(2p+1)(i-1)h_n},\xi\right)\\&\qquad\left[\left(\overline{X}_{(2p+1)ih_n,n}-\overline{X}_{((2p+1)i-1)h_n,n}-(h_nB)\,b\left(X_{(2p+1)(i-1)h_n}\right)\right)\right.\end{aligned}$$

$$\left(\left(\int_0^{\Delta_n}\Phi_{\Delta_n,n}\left(\Delta_n-s\right)\mathrm{d}s\right)b\left(X_{(2p+1)(i-1)}\right)\right)^T\Bigg]$$
$$\to^P \frac{1}{2p+1}\nu_0\left(\partial_x v\left[D_\ell^T\right](\cdot,\xi)\right) \text{ uniformly in } \xi\in\Xi$$

by an analogous manner to Gloter (2006). Hence we obtain

$$\frac{1}{nh_n}\sum_{i=1+\ell}^{n}\partial_x v\left(X_{(i-2p-1)h_n},\xi\right)\left[\left(\overline{X}_{ih_n,n}-\overline{X}_{(i-1)h_n,n}-\left(h_nB\right)b\left(X_{(i-2p-1)h_n}\right)\right)\right.$$
$$\left.\left(\overline{X}_{(i-1-\ell)h_n,n}-X_{(i-2p-1)h_n}\right)^T\right]$$
$$\to \nu_0\left(\partial_x v\left[D_\ell^T\right](\cdot,\xi)\right) \text{ uniformly in } \xi\in\Xi.$$

For the residual terms, it is obvious that they converge to zero in probability uniformly in $\xi\in\Xi$. Hence we complete the proof. □

Proof of Proposition 9. Because of the fact

$$\frac{1}{nh_n}\sum_{i=1}^{n}M\left(\overline{X}_{(i-1)h_n,n},\xi\right)\left[\left(\overline{X}_{ih_n,n}-\overline{X}_{(i-1)h_n,n}\right)^{\otimes 2}\right]$$
$$-\frac{1}{nh_n}\sum_{i=1}^{n}M\left(X_{(i-p-1)h_n},\xi\right)\left[\left(\overline{X}_{ih_n,n}-\overline{X}_{(i-1)h_n,n}\right)^{\otimes 2}\right]$$
$$\to^P 0 \text{ uniformly in } \xi\in\Xi$$

which can be easily obtained, it is sufficient to evaluate

$$\overline{Q}_n'\left(M(\cdot,\xi)\right)=\frac{1}{nh_n}\sum_{i=1}^{n}M\left(X_{(i-p-1)h_n},\xi\right)\left[\left(\overline{X}_{ih_n,n}-\overline{X}_{(i-1)h_n,n}\right)^{\otimes 2}\right],$$

and we can have an analogous result to Gloter (2006) such that

$$\overline{Q}_n'\left(M(\cdot,\xi)\right)\to^P \nu_0\left(M[G](\cdot,\xi)\right) \text{ uniformly in } \xi\in\Xi$$

because of the definition of G and hence obtain the proof. □

Remark 7. As the previous remark, we can obtain $G(x)=\frac{2}{3}A(x)$ as shown in Gloter (2006).

Some specific evaluation. We set $p=[\overline{\rho}]+1$, $\Delta_n=ph_n$ and show the evaluation of B, D_ℓ and G when setting our kernel $\{\Phi_{\Delta,n}\}=\{V_{\rho,h_n}\}$ as follows: we have $\Delta_n=ph_n$, $B=I_d$,

$D_0(x) = \mathbb{D}_0(x|\rho)|_{\rho=\rho_\star}$, where $\mathbb{D}_0^{(i,j)}(x|\rho) = A^{(i,j)}(x) f_{\mathbb{D}_0}\left(\rho^{(i)}, \rho^{(j)}\right)$,

$$f_{\mathbb{D}_0}\left(\rho^{(i)}, \rho^{(j)}\right) := \begin{cases} 0 & \text{if } \rho^{(j)} = 0, \\ \frac{\rho^{(j)}}{2} & \text{if } \rho^{(i)} = 0, \rho^{(j)} \in (0,1], \\ \frac{2\rho^{(j)}-1}{2\rho^{(j)}} & \text{if } \rho^{(i)} = 0, \rho^{(j)} \in (1,\overline{\rho}], \\ \frac{6\rho^{(i)}\rho^{(j)}-3\left(\rho^{(i)}\right)^2-3\rho^{(i)}}{6\rho^{(i)}\rho^{(j)}} & \text{if } \rho^{(i)} > 0, \rho^{(i)}+1 < \rho^{(j)}, \\ \frac{\left(\rho^{(i)}-\rho^{(j)}\right)^3+3\left(\rho^{(j)}\right)^2-3\rho^{(j)}+1}{6\rho^{(i)}\rho^{(j)}} & \text{if } \rho^{(j)} > 1, \rho^{(i)} < \rho^{(j)} \leq \rho^{(i)}+1, \\ \frac{3\left(\rho^{(j)}\right)^2-3\rho^{(j)}+1}{6\rho^{(i)}\rho^{(j)}} & \text{if } \rho^{(j)} > 1, \rho^{(i)} \geq \rho^{(j)}, \\ \frac{\left(\rho^{(i)}-\rho^{(j)}\right)^3+\left(\rho^{(j)}\right)^3}{6\rho^{(i)}\rho^{(j)}} & \text{if } \rho^{(j)} \in (0,1], \rho^{(i)} \in \left(0,\rho^{(j)}\right), \\ \frac{\left(\rho^{(j)}\right)^3}{6\rho^{(i)}\rho^{(j)}} & \text{if } \rho^{(j)} \in (0,1], \rho^{(i)} \geq \rho^{(j)}, \end{cases}$$

$D_\ell = O$ for $\ell \geq \left[\max_{i=1,\ldots,d} \rho_*^{(d)}\right] + 1$ because of independent increments of the Wiener process, and $G(x) = \mathbb{G}(x|\rho)|_{\rho=\rho_\star}$ where $\mathbb{G}(x|\rho) = \mathbb{G}(x,\alpha|\rho)|_{\alpha=\alpha_\star}$.

Remark 8. Note that $\mathbb{D}_0(x|\rho)$ and $\mathbb{G}(x|\rho)$ is continuous w.r.t. ρ for all fixed x by Lemma 15 and Lemma 16 in Appendix B.

For all $i = 1,\ldots,d$, if $\rho^{(i)} = 0$, then

$$\begin{aligned} &\left[\int_0^{\Delta_n+h_n} \left(V_{\rho,h_n}\left((\Delta_n+h_n)-s\right) - V_{\rho,h_n}\left(\Delta_n - s\right)\right) s\mathrm{d}s\right]^{(i,i)} \\ &= \int_0^{(p+1)h_n} \left(\delta\left((p+1)h_n - s\right) - \delta\left(ph_n - s\right)\right) s\mathrm{d}s \\ &= (p+1)h_n - ph_n \\ &= h_n, \end{aligned}$$

and if $\rho^{(i)} \in (0,1]$, then

$$\begin{aligned} &\left[\int_0^{\Delta_n+h_n} \left(V_{\rho,h_n}\left((\Delta_n+h_n)-s\right) - V_{\rho,h_n}\left(\Delta_n - s\right)\right) s\mathrm{d}s\right]^{(i,i)} \\ &= \int_0^{(p+1)h_n} \left(\rho^{(i)}h_n\right)^{-1} \left(\mathbf{1}_{[0,\rho^{(i)}h_n]}\left((p+1)h_n - s\right) - \mathbf{1}_{[0,\rho^{(i)}h_n]}\left(ph_n - s\right)\right) s\mathrm{d}s \\ &= \int_{\left(p+1-\rho^{(i)}\right)h_n}^{(p+1)h_n} \left(\rho^{(i)}h_n\right)^{-1} s\mathrm{d}s - \int_{\left(p-\rho^{(i)}\right)h_n}^{ph_n} \left(\rho^{(i)}h_n\right)^{-1} s\mathrm{d}s \\ &= \frac{h_n}{2\rho^{(i)}}\left[(p+1)^2 - \left(p+1-\rho^{(i)}\right)^2 - p^2 + \left(p-\rho^{(i)}\right)^2\right] \\ &= h_n, \end{aligned}$$

and if $\rho^{(i)} \in (1, \overline{\rho}]$, then

$$\begin{aligned}
&\left[\int_0^{\Delta_n+h_n} \left(V_{\rho,h_n}\left((\Delta_n+h_n)-s\right)-V_{\rho,h_n}\left(\Delta_n-s\right)\right)s\mathrm{d}s\right]^{(i,i)} \\
&=\int_{\left(p+1-\rho^{(i)}\right)h_n}^{(p+1)h_n} \left(\rho^{(i)}h_n\right)^{-1}s\mathrm{d}s-\int_{\left(p-\rho^{(i)}\right)h_n}^{ph_n}\left(\rho^{(i)}h_n\right)^{-1}s\mathrm{d}s \\
&=\int_{ph_n}^{(p+1)h_n} \left(\rho^{(i)}h_n\right)^{-1}s\mathrm{d}s-\int_{\left(p-\rho^{(i)}\right)h_n}^{\left(p+1-\rho^{(i)}\right)h_n}\left(\rho^{(i)}h_n\right)^{-1}s\mathrm{d}s \\
&=\frac{h_n}{2\rho^{(i)}}\left[(p+1)^2-p^2-\left(p+1-\rho^{(i)}\right)^2+\left(p-\rho^{(i)}\right)^2\right] \\
&=h_n.
\end{aligned}$$

It is obvious that if $i,j=1,\dots d$ and $i\neq j$,

$$\left[\int_0^{\Delta_n+h_n} \left(V_{\rho,h_n}\left((\Delta_n+h_n)-s\right)-V_{\rho,h_n}\left(\Delta_n-s\right)\right)s\mathrm{d}s\right]^{(i,j)}=0;$$

therefore it holds that $B=I_d$. Regarding to $D_0(x)$, for all $i,j=1,\dots,d$, let us define

$$\begin{aligned}
&\mathbf{D}_0^{(i,j)}(x|\rho) \\
&:=\frac{1}{h_n}\mathbf{E}\left[\left(\int_0^{(p+1)h_n}V_{\rho,h_n}\left(ph_n-s_1\right)\left(\int_0^{s_1}a(x)\mathrm{d}w_{s_2}\right)\mathrm{d}s_1\right)\right. \\
&\quad\left.\left(\int_0^{(p+1)h_n}\left(V_{\rho,h_n}\left((p+1)h_n-s_1\right)-V_{\rho,h_n}\left(ph_n-s_1\right)\right)\left(\int_0^{s_1}a(x)\mathrm{d}w_{s_2}\right)\mathrm{d}s_1\right)^T\right]^{(i,j)} \\
&=\frac{1}{h_n}\mathbf{E}\left[\left(\int_0^{(p+1)h_n}V_{\rho,h_n}\left(ph_n-s_1\right)\left(\int_0^{s_1}a(x)\mathrm{d}w_{s_2}\right)\mathrm{d}s_1\right)^{(i)}\right. \\
&\quad\left.\left(\int_0^{(p+1)h_n}\left(V_{\rho,h_n}\left((p+1)h_n-s_1\right)-V_{\rho,h_n}\left(ph_n-s_1\right)\right)\left(\int_0^{s_1}a(x)\mathrm{d}w_{s_2}\right)\mathrm{d}s_1\right)^{(j)}\right] \\
&=\frac{1}{h_n}\mathbf{E}\left[\left(\int_0^{(p+1)h_n}V_{\rho,h_n}^{(i,i)}\left(ph_n-s\right)\left(a(x)w_s\right)^{(i)}\mathrm{d}s\right)\right. \\
&\quad\left.\left(\int_0^{(p+1)h_n}\left(V_{\rho,h_n}^{(j,j)}\left((p+1)h_n-s_1'\right)-V_{\rho,h_n}^{(j,j)}\left(ph_n-s'\right)\right)\left(a(x)w_{s'}\right)^{(j)}\mathrm{d}s'\right)\right] \\
&=\frac{1}{h_n}\int_0^{(p+1)h_n}\int_0^{(p+1)h_n}\mathbf{E}\left[\left(a(x)w_s\right)^{(i)}\left(a(x)w_{s'}\right)^{(j)}\right] \\
&\quad\left(V_{\rho,h_n}^{(i,i)}\left(ph_n-s\right)\left(V_{\rho,h_n}^{(j,j)}\left((p+1)h_n-s'\right)-V_{\rho,h_n}^{(j,j)}\left(ph_n-s'\right)\right)\right)\mathrm{d}s'\mathrm{d}s \\
&=\frac{1}{h_n}\int_0^{(p+1)h_n}\int_0^{(p+1)h_n}A^{(i,j)}(x)\min\left\{s,s'\right\} \\
&\quad\left(V_{\rho,h_n}^{(i,i)}\left(ph_n-s\right)\left(V_{\rho,h_n}^{(j,j)}\left((p+1)h_n-s'\right)-V_{\rho,h_n}^{(j,j)}\left(ph_n-s'\right)\right)\right)\mathrm{d}s'\mathrm{d}s,
\end{aligned}$$

and if $\rho^{(i)}=\rho^{(j)}=0$,

$$h_n\mathbf{D}_0^{(i,j)}(x|\rho)=\int_0^{(p+1)h_n}\int_0^{(p+1)h_n}A^{(i,j)}(x)\min\{s,s'\}\left(V_{\rho,h_n}^{(i,i)}(ph_n-s)\left(V_{\rho,h_n}^{(j,j)}((p+1)h_n-s')-V_{\rho,h_n}^{(j,j)}(ph_n-s')\right)\right)\mathrm{d}s'\mathrm{d}s$$
$$=0,$$

and if $\rho^{(i)}=0,\ \rho^{(j)}\in(0,1]$,

$$\begin{aligned}
&h_n\mathbf{D}_0^{(i,j)}(x|\rho)\\
&=\int_0^{(p+1)h_n}\int_0^{(p+1)h_n}A^{(i,j)}(x)\min\{s,s'\}\left(V_{\rho,h_n}^{(i,i)}(ph_n-s)\left(V_{\rho,h_n}^{(j,j)}((p+1)h_n-s')-V_{\rho,h_n}^{(j,j)}(ph_n-s')\right)\right)\mathrm{d}s'\mathrm{d}s\\
&=\int_0^{(p+1)h_n}A^{(i,j)}(x)\min\{ph_n,s'\}\left(\rho^{(j)}h_n\right)^{-1}\left(\mathbf{1}_{\left[0,\rho^{(j)}h_n\right]}((p+1)h_n-s')-\mathbf{1}_{\left[0,\rho^{(j)}h_n\right]}(ph_n-s')\right)\mathrm{d}s'\\
&=\int_{ph_n}^{(p+1)h_n}A^{(i,j)}(x)ph_n\left(\rho^{(j)}h_n\right)^{-1}\left(\mathbf{1}_{\left[0,\rho^{(j)}h_n\right]}((p+1)h_n-s')-\mathbf{1}_{\left[0,\rho^{(j)}h_n\right]}(ph_n-s')\right)\mathrm{d}s'\\
&\quad+\int_0^{ph_n}A^{(i,j)}(x)s'\left(\rho^{(j)}h_n\right)^{-1}\left(\mathbf{1}_{\left[0,\rho^{(j)}h_n\right]}((p+1)h_n-s')-\mathbf{1}_{\left[0,\rho^{(j)}h_n\right]}(ph_n-s')\right)\mathrm{d}s'\\
&=A^{(i,j)}(x)\left(ph_n-\frac{p^2h_n^2-\left(p-\rho^{(j)}\right)^2h_n^2}{2\rho^{(j)}h_n}\right)\\
&=\frac{\rho^{(j)}h_nA^{(i,j)}(x)}{2},
\end{aligned}$$

and if $\rho^{(i)}=0,\ \rho^{(j)}\in(1,\overline{\rho}]$,

$$\begin{aligned}
&h_n\mathbf{D}_0^{(i,j)}(x|\rho)\\
&=\int_0^{(p+1)h_n}\int_0^{(p+1)h_n}A^{(i,j)}(x)\min\{s,s'\}\left(V_{\rho,h_n}^{(i,i)}(ph_n-s)\left(V_{\rho,h_n}^{(j,j)}((p+1)h_n-s')-V_{\rho,h_n}^{(j,j)}(ph_n-s')\right)\right)\mathrm{d}s'\mathrm{d}s\\
&=\int_{ph_n}^{(p+1)h_n}A^{(i,j)}(x)ph_n\left(\rho^{(j)}h_n\right)^{-1}\left(\mathbf{1}_{\left[0,\rho^{(j)}h_n\right]}((p+1)h_n-s')-\mathbf{1}_{\left[0,\rho^{(j)}h_n\right]}(ph_n-s')\right)\mathrm{d}s'\\
&\quad+\int_0^{ph_n}A^{(i,j)}(x)s'\left(\rho^{(j)}h_n\right)^{-1}\left(\mathbf{1}_{\left[0,\rho^{(j)}h_n\right]}((p+1)h_n-s')-\mathbf{1}_{\left[0,\rho^{(j)}h_n\right]}(ph_n-s')\right)\mathrm{d}s'
\end{aligned}$$

$$
\begin{aligned}
&= A^{(i,j)}(x)\left(\frac{ph_n}{\rho^{(j)}} + \frac{p^2h_n^2 - \left(p+1-\rho^{(j)}\right)^2 h_n^2 - p^2h_n^2 + \left(p-\rho^{(j)}\right)^2 h_n^2}{2\rho^{(j)}h_n}\right)\\
&= A^{(i,j)}(x)\left(\frac{ph_n}{\rho^{(j)}} + \frac{-2\left(p-\rho^{(j)}\right)h_n^2 - h_n^2}{2\rho^{(j)}h_n}\right)\\
&= \frac{\left(2\rho^{(j)}-1\right)h_n A^{(i,j)}(x)}{2\rho^{(j)}},
\end{aligned}
$$

and if $\rho^{(i)} > 0$, $\rho^{(j)} = 0$,

$$
\begin{aligned}
&h_n\mathbf{D}_0^{(i,j)}(x|\rho)\\
&= \int_0^{(p+1)h_n}\int_0^{(p+1)h_n} A^{(i,j)}(x)\min\{s,s'\}\\
&\qquad \left(V_{\rho,h_n}^{(i,i)}(ph_n - s)\left(V_{\rho,h_n}^{(j,j)}((p+1)h_n - s') - V_{\rho,h_n}^{(j,j)}(ph_n - s')\right)\right)\mathrm{d}s'\mathrm{d}s\\
&= \int_0^{(p+1)h_n} A^{(i,j)}(x)\min\{s,(p+1)h_n\}\left(\rho^{(i)}h_n\right)^{-1}\mathbf{1}_{\left[0,\rho^{(i)}h_n\right]}(ph_n - s)\,\mathrm{d}s\\
&\quad - \int_0^{(p+1)h_n} A^{(i,j)}(x)\min\{s,ph_n\}\left(\rho^{(i)}h_n\right)^{-1}\mathbf{1}_{\left[0,\rho^{(i)}h_n\right]}(ph_n - s)\,\mathrm{d}s\\
&= \int_0^{(p+1)h_n} A^{(i,j)}(x)\,s\left(\rho^{(i)}h_n\right)^{-1}\mathbf{1}_{\left[0,\rho^{(i)}h_n\right]}(ph_n - s)\,\mathrm{d}s\\
&\quad - \int_{ph_n}^{(p+1)h_n} A^{(i,j)}(x)\,ph_n\left(\rho^{(i)}h_n\right)^{-1}\mathbf{1}_{\left[0,\rho^{(i)}h_n\right]}(ph_n - s)\,\mathrm{d}s\\
&\quad - \int_0^{ph_n} A^{(i,j)}(x)\,s\left(\rho^{(i)}h_n\right)^{-1}\mathbf{1}_{\left[0,\rho^{(i)}h_n\right]}(ph_n - s)\,\mathrm{d}s\\
&= 0,
\end{aligned}
$$

and if $\rho^{(i)} > 0$, $\rho^{(j)} > 0$,

$$
\begin{aligned}
&h_n\mathbf{D}_0^{(i,j)}(x|\rho)\\
&= \int_0^{(p+1)h_n}\int_0^{(p+1)h_n} A^{(i,j)}(x)\min\{s,s'\}\\
&\qquad \left(V_{\rho,h_n}^{(i,i)}(ph_n - s)\left(V_{\rho,h_n}^{(j,j)}((p+1)h_n - s') - V_{\rho,h_n}^{(j,j)}(ph_n - s')\right)\right)\mathrm{d}s'\mathrm{d}s\\
&= \frac{A^{(i,j)}(x)}{\rho^{(i)}\rho^{(j)}h_n^2}\int_0^{(p+1)h_n}\int_0^{(p+1)h_n}\min\{s,s'\}\,\mathbf{1}_{\left[0,\rho^{(i)}h_n\right]}(ph_n - s)\\
&\qquad \left(\mathbf{1}_{\left[0,\rho^{(j)}h_n\right]}((p+1)h_n - s') - \mathbf{1}_{\left[0,\rho^{(j)}h_n\right]}(ph_n - s')\right)\mathrm{d}s'\mathrm{d}s\\
&= \frac{A^{(i,j)}(x)}{\rho^{(i)}\rho^{(j)}h_n^2}\int_0^{(p+1)h_n}\int_s^{(p+1)h_n} s\mathbf{1}_{\left[0,\rho^{(i)}h_n\right]}(ph_n - s)\\
&\qquad\qquad \left(\mathbf{1}_{\left[0,\rho^{(j)}h_n\right]}((p+1)h_n - s') - \mathbf{1}_{\left[0,\rho^{(j)}h_n\right]}(ph_n - s')\right)\mathrm{d}s'\mathrm{d}s\\
&\quad + \frac{A^{(i,j)}(x)}{\rho^{(i)}\rho^{(j)}h_n^2}\int_0^{(p+1)h_n}\int_0^{s} s'\mathbf{1}_{\left[0,\rho^{(i)}h_n\right]}(ph_n - s)
\end{aligned}
$$

$$
\begin{aligned}
&\qquad\qquad \left(\mathbf{1}_{\left[0,\rho^{(j)}h_n\right]}\left(\left(p+1\right)h_n-s'\right)-\mathbf{1}_{\left[0,\rho^{(j)}h_n\right]}\left(ph_n-s'\right)\right)\mathrm{d}s'\mathrm{d}s\\
&=\frac{A^{(i,j)}\left(x\right)}{\rho^{(i)}\rho^{(j)}h_n^2}\int_0^{(p+1)h_n}s\mathbf{1}_{\left[0,\rho^{(i)}h_n\right]}\left(ph_n-s\right)\\
&\qquad\int_s^{(p+1)h_n}\left(\mathbf{1}_{\left[0,\rho^{(j)}h_n\right]}\left(\left(p+1\right)h_n-s'\right)-\mathbf{1}_{\left[0,\rho^{(j)}h_n\right]}\left(ph_n-s'\right)\right)\mathrm{d}s'\mathrm{d}s\\
&\quad+\frac{A^{(i,j)}\left(x\right)}{\rho^{(i)}\rho^{(j)}h_n^2}\int_0^{(p+1)h_n}\mathbf{1}_{\left[0,\rho^{(i)}h_n\right]}\left(ph_n-s\right)\\
&\qquad\int_0^{s}s'\left(\mathbf{1}_{\left[0,\rho^{(j)}h_n\right]}\left(\left(p+1\right)h_n-s'\right)-\mathbf{1}_{\left[0,\rho^{(j)}h_n\right]}\left(ph_n-s'\right)\right)\mathrm{d}s'\mathrm{d}s\\
&=\frac{A^{(i,j)}\left(x\right)}{\rho^{(i)}\rho^{(j)}h_n^2}\int_0^{(p+1)h_n}s\mathbf{1}_{\left[0,\rho^{(i)}h_n\right]}\left(ph_n-s\right)\\
&\qquad\left[\left(p+1\right)h_n-\max\left\{s,\left(p+1-\rho^{(j)}\right)h_n\right\}\right.\\
&\qquad\qquad\left.-\mathbf{1}_{\left[0,ph_n\right]}\left(s\right)\left(ph_n-\max\left\{s,\left(p-\rho^{(j)}\right)h_n\right\}\right)\right]\mathrm{d}s\\
&\quad+\frac{A^{(i,j)}\left(x\right)}{\rho^{(i)}\rho^{(j)}h_n^2}\int_0^{(p+1)h_n}\mathbf{1}_{\left[0,\rho^{(i)}h_n\right]}\left(ph_n-s\right)\\
&\qquad\left[\mathbf{1}_{\left[\left(p+1-\rho^{(j)}\right)h_n,(p+1)h_n\right]}\left(s\right)\frac{1}{2}\left(s^2-\left(p+1-\rho^{(j)}\right)^2h_n^2\right)\right.\\
&\qquad\qquad\left.-\mathbf{1}_{\left[\left(p-\rho^{(j)}\right)h_n,(p+1)h_n\right]}\left(s\right)\frac{1}{2}\left(\min\left\{s^2,p^2h_n^2\right\}-\left(p-\rho^{(j)}\right)^2h_n^2\right)\right]\mathrm{d}s\\
&=\frac{A^{(i,j)}\left(x\right)\left(p+1\right)}{\rho^{(i)}\rho^{(j)}h_n}\int_0^{(p+1)h_n}s\mathbf{1}_{\left[0,\rho^{(i)}h_n\right]}\left(ph_n-s\right)\mathrm{d}s\\
&\quad-\frac{A^{(i,j)}\left(x\right)}{\rho^{(i)}\rho^{(j)}h_n^2}\int_0^{(p+1)h_n}s\mathbf{1}_{\left[0,\rho^{(i)}h_n\right]}\left(ph_n-s\right)\max\left\{s,\left(p+1-\rho^{(j)}\right)h_n\right\}\mathrm{d}s\\
&\quad-\frac{A^{(i,j)}\left(x\right)p}{\rho^{(i)}\rho^{(j)}h_n}\int_0^{(p+1)h_n}s\mathbf{1}_{\left[0,\rho^{(i)}h_n\right]}\left(ph_n-s\right)\mathbf{1}_{\left[0,ph_n\right]}\left(s\right)\mathrm{d}s\\
&\quad+\frac{A^{(i,j)}\left(x\right)}{\rho^{(i)}\rho^{(j)}h_n^2}\int_0^{(p+1)h_n}s\mathbf{1}_{\left[0,\rho^{(i)}h_n\right]}\left(ph_n-s\right)\mathbf{1}_{\left[0,ph_n\right]}\left(s\right)\max\left\{s,\left(p-\rho^{(j)}\right)h_n\right\}\mathrm{d}s\\
&\quad+\frac{A^{(i,j)}\left(x\right)}{2\rho^{(i)}\rho^{(j)}h_n^2}\int_0^{(p+1)h_n}\mathbf{1}_{\left[0,\rho^{(i)}h_n\right]}\left(ph_n-s\right)\mathbf{1}_{\left[\left(p+1-\rho^{(j)}\right)h_n,(p+1)h_n\right]}\left(s\right)s^2\mathrm{d}s\\
&\quad-\frac{A^{(i,j)}\left(x\right)\left(p+1-\rho^{(j)}\right)^2}{2\rho^{(i)}\rho^{(j)}}\int_0^{(p+1)h_n}\mathbf{1}_{\left[0,\rho^{(i)}h_n\right]}\left(ph_n-s\right)\mathbf{1}_{\left[\left(p+1-\rho^{(j)}\right)h_n,(p+1)h_n\right]}\left(s\right)\mathrm{d}s\\
&\quad-\frac{A^{(i,j)}\left(x\right)}{2\rho^{(i)}\rho^{(j)}h_n^2}\int_0^{(p+1)h_n}\mathbf{1}_{\left[0,\rho^{(i)}h_n\right]}\left(ph_n-s\right)\mathbf{1}_{\left[\left(p-\rho^{(j)}\right)h_n,(p+1)h_n\right]}\left(s\right)\min\left\{s^2,p^2h_n^2\right\}\mathrm{d}s\\
&\quad+\frac{A^{(i,j)}\left(x\right)\left(p-\rho^{(j)}\right)^2}{2\rho^{(i)}\rho^{(j)}}\int_0^{(p+1)h_n}\mathbf{1}_{\left[0,\rho^{(i)}h_n\right]}\left(ph_n-s\right)\mathbf{1}_{\left[\left(p-\rho^{(j)}\right)h_n,(p+1)h_n\right]}\left(s\right)\mathrm{d}s\\
&=\frac{A^{(i,j)}\left(x\right)\left(p+1\right)}{\rho^{(i)}\rho^{(j)}h_n}\int_{\left(p-\rho^{(i)}\right)h_n}^{ph_n}s\mathrm{d}s
\end{aligned}
$$

$$
\begin{aligned}
&- \frac{A^{(i,j)}(x)}{\rho^{(i)}\rho^{(j)}h_n^2} \int_{\left(p+1-\rho^{(j)}\right)h_n}^{(p+1)h_n} s^2 \mathbf{1}_{\left[0,\rho^{(i)}h_n\right]}(ph_n - s)\,\mathrm{d}s \\
&- \frac{A^{(i,j)}(x)\left(p+1-\rho^{(j)}\right)}{\rho^{(i)}\rho^{(j)}h_n} \int_0^{\left(p+1-\rho^{(j)}\right)h_n} s\mathbf{1}_{\left[0,\rho^{(i)}h_n\right]}(ph_n - s)\,\mathrm{d}s \\
&- \frac{A^{(i,j)}(x)\,p}{\rho^{(i)}\rho^{(j)}h_n} \int_{\left(p-\rho^{(i)}\right)h_n}^{ph_n} s\mathrm{d}s \\
&+ \frac{A^{(i,j)}(x)}{\rho^{(i)}\rho^{(j)}h_n^2} \int_{\left(p-\rho^{(j)}\right)h_n}^{(p+1)h_n} s^2 \mathbf{1}_{\left[0,\rho^{(i)}h_n\right]}(ph_n - s)\,\mathbf{1}_{[0,ph_n]}(s)\,\mathrm{d}s \\
&+ \frac{A^{(i,j)}(x)\left(p-\rho^{(j)}\right)}{\rho^{(i)}\rho^{(j)}h_n} \int_0^{\left(p-\rho^{(j)}\right)h_n} s\mathbf{1}_{\left[0,\rho^{(i)}h_n\right]}(ph_n - s)\,\mathbf{1}_{[0,ph_n]}(s)\,\mathrm{d}s \\
&+ \frac{A^{(i,j)}(x)}{2\rho^{(i)}\rho^{(j)}h_n^2} \int_{\left(p-\rho^{(i)}\right)h_n}^{ph_n} \mathbf{1}_{\left[\left(p+1-\rho^{(j)}\right)h_n,(p+1)h_n\right]}(s)\,s^2\mathrm{d}s \\
&- \frac{A^{(i,j)}(x)\left(p+1-\rho^{(j)}\right)^2}{2\rho^{(i)}\rho^{(j)}} \int_{\left(p-\rho^{(i)}\right)h_n}^{ph_n} \mathbf{1}_{\left[\left(p+1-\rho^{(j)}\right)h_n,(p+1)h_n\right]}(s)\,\mathrm{d}s \\
&- \frac{A^{(i,j)}(x)}{2\rho^{(i)}\rho^{(j)}h_n^2} \int_{\left(p-\rho^{(i)}\right)h_n}^{ph_n} \mathbf{1}_{\left[\left(p-\rho^{(j)}\right)h_n,(p+1)h_n\right]}(s)\,s^2\mathrm{d}s \\
&+ \frac{A^{(i,j)}(x)\left(p-\rho^{(j)}\right)^2}{2\rho^{(i)}\rho^{(j)}} \int_{\left(p-\rho^{(i)}\right)h_n}^{ph_n} \mathbf{1}_{\left[\left(p-\rho^{(j)}\right)h_n,(p+1)h_n\right]}(s)\,\mathrm{d}s \\
=& \frac{A^{(i,j)}(x)(p+1)}{2\rho^{(i)}\rho^{(j)}h_n}\left(p^2h_n^2 - \left(p-\rho^{(i)}\right)^2 h_n^2\right) \\
&- \frac{A^{(i,j)}(x)\,\mathbf{1}_{(1,\bar{\rho}]}\left(\rho^{(j)}\right)}{\rho^{(i)}\rho^{(j)}h_n^2} \int_{\max\left\{\left(p-\rho^{(i)}\right),\left(p+1-\rho^{(j)}\right)\right\}h_n}^{ph_n} s^2\mathrm{d}s \\
&- \frac{A^{(i,j)}(x)\left(p+1-\rho^{(j)}\right)\mathbf{1}_{\left(0,\rho^{(i)}+1\right]}\left(\rho^{(j)}\right)}{\rho^{(i)}\rho^{(j)}h_n} \int_{\left(p-\rho^{(i)}\right)h_n}^{\min\left\{p,\left(p+1-\rho^{(j)}\right)\right\}h_n} s\mathrm{d}s \\
&- \frac{A^{(i,j)}(x)\,p}{2\rho^{(i)}\rho^{(j)}h_n}\left(p^2h_n^2 - \left(p-\rho^{(i)}\right)^2 h_n^2\right) \\
&+ \frac{A^{(i,j)}(x)}{\rho^{(i)}\rho^{(j)}h_n^2} \int_{\left(p-\rho^{(j)}\right)h_n}^{ph_n} s^2 \mathbf{1}_{\left[0,\rho^{(i)}h_n\right]}(ph_n - s)\,\mathrm{d}s \\
&+ \frac{A^{(i,j)}(x)\left(p-\rho^{(j)}\right)}{\rho^{(i)}\rho^{(j)}h_n} \int_0^{\left(p-\rho^{(j)}\right)h_n} s\mathbf{1}_{\left[0,\rho^{(i)}h_n\right]}(ph_n - s)\,\mathrm{d}s \\
&+ \frac{A^{(i,j)}(x)\,\mathbf{1}_{(1,\bar{\rho}]}\left(\rho^{(j)}\right)}{2\rho^{(i)}\rho^{(j)}h_n^2} \int_{\max\left\{\left(p-\rho^{(i)}\right),\left(p+1-\rho^{(j)}\right)\right\}h_n}^{ph_n} s^2\mathrm{d}s \\
&- \frac{A^{(i,j)}(x)\left(p+1-\rho^{(j)}\right)^2\mathbf{1}_{(1,\bar{\rho}]}\left(\rho^{(j)}\right)}{2\rho^{(i)}\rho^{(j)}} \int_{\max\left\{\left(p-\rho^{(i)}\right),\left(p+1-\rho^{(j)}\right)\right\}h_n}^{ph_n} \mathrm{d}s
\end{aligned}
$$

$$
\begin{aligned}
&- \frac{A^{(i,j)}(x)}{2\rho^{(i)}\rho^{(j)}h_n^2}\int_{\max\left\{\left(p-\rho^{(i)}\right),\left(p-\rho^{(j)}\right)\right\}h_n}^{ph_n} s^2 \mathrm{d}s \\
&+ \frac{A^{(i,j)}(x)\left(p-\rho^{(j)}\right)^2}{2\rho^{(i)}\rho^{(j)}}\int_{\max\left\{\left(p-\rho^{(i)}\right),\left(p-\rho^{(j)}\right)\right\}h_n}^{ph_n} \mathrm{d}s \\
= &\frac{A^{(i,j)}(x)(p+1)h_n}{2\rho^{(i)}\rho^{(j)}}\left(p^2-\left(p-\rho^{(i)}\right)^2\right) \\
&- \frac{A^{(i,j)}(x)\mathbf{1}_{(1,\bar{\rho}]}\left(\rho^{(j)}\right)h_n}{3\rho^{(i)}\rho^{(j)}}\left(p^3-\max\left\{\left(p-\rho^{(i)}\right)^3,\left(p+1-\rho^{(j)}\right)^3\right\}\right) \\
&- \frac{A^{(i,j)}(x)\left(p+1-\rho^{(j)}\right)\mathbf{1}_{\left(0,\rho^{(i)}+1\right]}\left(\rho^{(j)}\right)h_n}{2\rho^{(i)}\rho^{(j)}}\left(\min\left\{p^2,\left(p+1-\rho^{(j)}\right)^2\right\}-\left(p-\rho^{(i)}\right)^2\right) \\
&- \frac{A^{(i,j)}(x)ph_n}{2\rho^{(i)}\rho^{(j)}}\left(p^2-\left(p-\rho^{(i)}\right)^2\right) \\
&+ \frac{A^{(i,j)}(x)}{\rho^{(i)}\rho^{(j)}h_n^2}\int_{\max\left\{\left(p-\rho^{(i)}\right),\left(p-\rho^{(j)}\right)\right\}h_n}^{ph_n} s^2 \mathrm{d}s \\
&+ \frac{A^{(i,j)}(x)\left(p-\rho^{(j)}\right)\mathbf{1}_{\left(0,\rho^{(i)}\right]}\left(\rho^{(j)}\right)}{\rho^{(i)}\rho^{(j)}h_n}\int_{\left(p-\rho^{(i)}\right)h_n}^{\left(p-\rho^{(j)}\right)h_n} s\mathrm{d}s \\
&+ \frac{A^{(i,j)}(x)\mathbf{1}_{(1,\bar{\rho}]}\left(\rho^{(j)}\right)h_n}{6\rho^{(i)}\rho^{(j)}}\left(p^3-\max\left\{\left(p-\rho^{(i)}\right)^3,\left(p+1-\rho^{(j)}\right)^3\right\}\right) \\
&- \frac{A^{(i,j)}(x)\left(p+1-\rho^{(j)}\right)^2\mathbf{1}_{(1,\bar{\rho}]}\left(\rho^{(j)}\right)h_n}{2\rho^{(i)}\rho^{(j)}}\left(p-\max\left\{\left(p-\rho^{(i)}\right),\left(p+1-\rho^{(j)}\right)\right\}\right) \\
&- \frac{A^{(i,j)}(x)h_n}{6\rho^{(i)}\rho^{(j)}}\left(p^3-\max\left\{\left(p-\rho^{(i)}\right)^3,\left(p-\rho^{(j)}\right)^3\right\}\right) \\
&+ \frac{A^{(i,j)}(x)\left(p-\rho^{(j)}\right)^2h_n}{2\rho^{(i)}\rho^{(j)}}\left(p-\max\left\{\left(p-\rho^{(i)}\right),\left(p-\rho^{(j)}\right)\right\}\right) \\
= &\frac{A^{(i,j)}(x)h_n}{2\rho^{(i)}\rho^{(j)}}\left(p^2-\left(p-\rho^{(i)}\right)^2\right) \\
&- \frac{A^{(i,j)}(x)\mathbf{1}_{(1,\bar{\rho}]}\left(\rho^{(j)}\right)h_n}{6\rho^{(i)}\rho^{(j)}}\left(p^3-\max\left\{\left(p-\rho^{(i)}\right)^3,\left(p+1-\rho^{(j)}\right)^3\right\}\right) \\
&- \frac{A^{(i,j)}(x)\left(p+1-\rho^{(j)}\right)\mathbf{1}_{\left(0,\rho^{(i)}+1\right]}\left(\rho^{(j)}\right)h_n}{2\rho^{(i)}\rho^{(j)}}\left(\min\left\{p^2,\left(p+1-\rho^{(j)}\right)^2\right\}-\left(p-\rho^{(i)}\right)^2\right) \\
&+ \frac{A^{(i,j)}(x)h_n}{6\rho^{(i)}\rho^{(j)}}\left(p^3-\max\left\{\left(p-\rho^{(i)}\right)^3,\left(p-\rho^{(j)}\right)^3\right\}\right) \\
&+ \frac{A^{(i,j)}(x)\left(p-\rho^{(j)}\right)\mathbf{1}_{\left(0,\rho^{(i)}\right]}\left(\rho^{(j)}\right)h_n}{2\rho^{(i)}\rho^{(j)}}\left(\left(p-\rho^{(j)}\right)^2-\left(p-\rho^{(i)}\right)^2\right) \\
&- \frac{A^{(i,j)}(x)\left(p+1-\rho^{(j)}\right)^2\mathbf{1}_{(1,\bar{\rho}]}\left(\rho^{(j)}\right)h_n}{2\rho^{(i)}\rho^{(j)}}\left(p-\max\left\{\left(p-\rho^{(i)}\right),\left(p+1-\rho^{(j)}\right)\right\}\right)
\end{aligned}
$$

$$+\frac{A^{(i,j)}(x)\left(p-\rho^{(j)}\right)^2 h_n}{2\rho^{(i)}\rho^{(j)}}\left(p-\max\left\{\left(p-\rho^{(i)}\right),\left(p-\rho^{(j)}\right)\right\}\right)$$

and then we should consider five cases as follows: (i) $\rho^{(i)}+1<\rho^{(j)}$; (ii) $\rho^{(j)}>1$, $\rho^{(i)}<\rho^{(j)}\leq\rho^{(i)}+1$; (iii) $\rho^{(j)}>1$, $\rho^{(i)}\geq\rho^{(j)}$; (iv) $\rho^{(j)}\leq 1$, $\rho^{(i)}<\rho^{(j)}$; (v) $\rho^{(j)}\leq 1$, $\rho^{(i)}\geq\rho^{(j)}$, and for the case (i), we have

$$\begin{aligned}
&h_n\mathbf{D}_0^{(i,j)}(x|\rho)\\
&=\int_0^{(p+1)h_n}\int_0^{(p+1)h_n}A^{(i,j)}(x)\min\{s,s'\}\\
&\quad\left(V_{\rho,h_n}^{(i,i)}(ph_n-s)\left(V_{\rho,h_n}^{(j,j)}((p+1)h_n-s')-V_{\rho,h_n}^{(j,j)}(ph_n-s')\right)\right)\mathrm{d}s'\mathrm{d}s\\
&=\frac{A^{(i,j)}(x)h_n}{2\rho^{(i)}\rho^{(j)}}\left(p^2-\left(p-\rho^{(i)}\right)^2\right)-\frac{A^{(i,j)}(x)h_n}{6\rho^{(i)}\rho^{(j)}}\left(p^3-\left(p-\rho^{(i)}\right)^3\right)\\
&\quad+\frac{A^{(i,j)}(x)h_n}{6\rho^{(i)}\rho^{(j)}}\left(p^3-\left(p-\rho^{(i)}\right)^3\right)-\frac{A^{(i,j)}(x)\left(p+1-\rho^{(j)}\right)^2 h_n}{2\rho^{(i)}\rho^{(j)}}\left(p-\left(p-\rho^{(i)}\right)\right)\\
&\quad+\frac{A^{(i,j)}(x)\left(p-\rho^{(j)}\right)^2 h_n}{2\rho^{(i)}\rho^{(j)}}\left(p-\left(p-\rho^{(i)}\right)\right)\\
&=\frac{A^{(i,j)}(x)h_n}{2\rho^{(i)}\rho^{(j)}}\left(p^2-\left(p-\rho^{(i)}\right)^2\right)\\
&\quad-\frac{A^{(i,j)}(x)\left(p+1-\rho^{(j)}\right)^2\rho^{(i)}h_n}{2\rho^{(i)}\rho^{(j)}}+\frac{A^{(i,j)}(x)\left(p-\rho^{(j)}\right)^2\rho^{(i)}h_n}{2\rho^{(i)}\rho^{(j)}}\\
&=\frac{A^{(i,j)}(x)h_n}{2\rho^{(i)}\rho^{(j)}}\left(p^2-\left(p-\rho^{(i)}\right)^2-2\left(p-\rho^{(j)}\right)\rho^{(i)}-\rho^{(i)}\right)\\
&=\frac{A^{(i,j)}(x)h_n}{2\rho^{(i)}\rho^{(j)}}\left(p^2-p^2+2p\rho^{(i)}-\left(\rho^{(i)}\right)^2-2p\rho^{(i)}+2\rho^{(i)}\rho^{(j)}-\rho^{(i)}\right)\\
&=\frac{A^{(i,j)}(x)h_n}{6\rho^{(i)}\rho^{(j)}}\left(6\rho^{(i)}\rho^{(j)}-3\left(\rho^{(i)}\right)^2-3\rho^{(i)}\right),
\end{aligned}$$

and for the case (ii),

$$\begin{aligned}
&h_n\mathbf{D}_0^{(i,j)}(x|\rho)\\
&=\int_0^{(p+1)h_n}\int_0^{(p+1)h_n}A^{(i,j)}(x)\min\{s,s'\}\\
&\quad\left(V_{\rho,h_n}^{(i,i)}(ph_n-s)\left(V_{\rho,h_n}^{(j,j)}((p+1)h_n-s')-V_{\rho,h_n}^{(j,j)}(ph_n-s')\right)\right)\mathrm{d}s'\mathrm{d}s\\
&=\frac{A^{(i,j)}(x)h_n}{2\rho^{(i)}\rho^{(j)}}\left(p^2-\left(p-\rho^{(i)}\right)^2\right)-\frac{A^{(i,j)}(x)h_n}{6\rho^{(i)}\rho^{(j)}}\left(p^3-\left(p+1-\rho^{(j)}\right)^3\right)\\
&\quad-\frac{A^{(i,j)}(x)\left(p+1-\rho^{(j)}\right)h_n}{2\rho^{(i)}\rho^{(j)}}\left(\left(p+1-\rho^{(j)}\right)^2-\left(p-\rho^{(i)}\right)^2\right)\\
&\quad+\frac{A^{(i,j)}(x)h_n}{6\rho^{(i)}\rho^{(j)}}\left(p^3-\left(p-\rho^{(i)}\right)^3\right)
\end{aligned}$$

$$
\begin{aligned}
&- \frac{A^{(i,j)}(x)\left(p+1-\rho^{(j)}\right)^2 h_n}{2\rho^{(i)}\rho^{(j)}}\left(p-\left(p+1-\rho^{(j)}\right)\right) \\
&+ \frac{A^{(i,j)}(x)\left(p-\rho^{(j)}\right)^2 h_n}{2\rho^{(i)}\rho^{(j)}}\left(p-\left(p-\rho^{(i)}\right)\right) \\
&= \frac{A^{(i,j)}(x)h_n}{6\rho^{(i)}\rho^{(j)}}\Big[3\left(p^2-\left(p-\rho^{(i)}\right)^2\right)-\left(p^3-\left(p+1-\rho^{(j)}\right)^3\right) \\
&\qquad -3\left(p+1-\rho^{(j)}\right)\left(\left(p+1-\rho^{(j)}\right)^2-\left(p-\rho^{(i)}\right)^2\right) \\
&\qquad +\left(p^3-\left(p-\rho^{(i)}\right)^3\right) \\
&\qquad -3\left(p+1-\rho^{(j)}\right)^2\left(p-\left(p+1-\rho^{(j)}\right)\right) \\
&\qquad +3\left(p-\rho^{(j)}\right)^2\left(p-\left(p-\rho^{(i)}\right)\right)\Big] \\
&= \frac{A^{(i,j)}(x)h_n}{6\rho^{(i)}\rho^{(j)}}\left[\left(\rho^{(i)}-\rho^{(j)}\right)^3+3\left(\rho^{(j)}\right)^2-3\rho^{(j)}+1\right],
\end{aligned}
$$

and for the case (iii),

$$
\begin{aligned}
&h_n\mathbf{D}_0^{(i,j)}(x|\rho) \\
&= \int_0^{(p+1)h_n}\int_0^{(p+1)h_n} A^{(i,j)}(x)\min\{s,s'\} \\
&\qquad \left(V_{\rho,h_n}^{(i,i)}(ph_n-s)\left(V_{\rho,h_n}^{(j,j)}((p+1)h_n-s')-V_{\rho,h_n}^{(j,j)}(ph_n-s')\right)\right)\mathrm{d}s'\mathrm{d}s \\
&= \frac{A^{(i,j)}(x)h_n}{2\rho^{(i)}\rho^{(j)}}\left(p^2-\left(p-\rho^{(i)}\right)^2\right)-\frac{A^{(i,j)}(x)h_n}{6\rho^{(i)}\rho^{(j)}}\left(p^3-\left(p+1-\rho^{(j)}\right)^3\right) \\
&\quad - \frac{A^{(i,j)}(x)\left(p+1-\rho^{(j)}\right)h_n}{2\rho^{(i)}\rho^{(j)}}\left(\left(p+1-\rho^{(j)}\right)^2-\left(p-\rho^{(i)}\right)^2\right) \\
&\quad + \frac{A^{(i,j)}(x)h_n}{6\rho^{(i)}\rho^{(j)}}\left(p^3-\left(p-\rho^{(j)}\right)^3\right) \\
&\quad + \frac{A^{(i,j)}(x)\left(p-\rho^{(j)}\right)h_n}{2\rho^{(i)}\rho^{(j)}}\left(\left(p-\rho^{(j)}\right)^2-\left(p-\rho^{(i)}\right)^2\right) \\
&\quad - \frac{A^{(i,j)}(x)\left(p+1-\rho^{(j)}\right)^2 h_n}{2\rho^{(i)}\rho^{(j)}}\left(p-\left(p+1-\rho^{(j)}\right)\right) \\
&\quad + \frac{A^{(i,j)}(x)\left(p-\rho^{(j)}\right)^2 h_n}{2\rho^{(i)}\rho^{(j)}}\left(p-\left(p-\rho^{(j)}\right)\right) \\
&= \frac{A^{(i,j)}(x)h_n}{6\rho^{(i)}\rho^{(j)}}\Big[3\left(p^2-\left(p-\rho^{(i)}\right)^2\right)-\left(p^3-\left(p+1-\rho^{(j)}\right)^3\right) \\
&\qquad -3\left(p+1-\rho^{(j)}\right)\left(\left(p+1-\rho^{(j)}\right)^2-\left(p-\rho^{(i)}\right)^2\right) \\
&\qquad +\left(p^3-\left(p-\rho^{(j)}\right)^3\right)+3\left(p-\rho^{(j)}\right)\left(\left(p-\rho^{(j)}\right)^2-\left(p-\rho^{(i)}\right)^2\right) \\
&\qquad -3\left(p+1-\rho^{(j)}\right)^2\left(p-\left(p+1-\rho^{(j)}\right)\right)+3\left(p-\rho^{(j)}\right)^2\rho^{(j)}\Big]
\end{aligned}
$$

$$= \frac{A^{(i,j)}(x)\,h_n}{6\rho^{(i)}\rho^{(j)}}\left[3\left(\rho^{(j)}\right)^2 - 3\rho^{(j)} + 1\right]$$

and for the case (iv),

$$\begin{aligned}
h_n \mathbf{D}_0^{(i,j)}(x|\rho) &= \int_0^{(p+1)h_n}\int_0^{(p+1)h_n} A^{(i,j)}(x)\min\{s,s'\} \\
&\qquad \left(V_{\rho,h_n}^{(i,i)}(ph_n - s)\left(V_{\rho,h_n}^{(j,j)}((p+1)h_n - s') - V_{\rho,h_n}^{(j,j)}(ph_n - s')\right)\right)\mathrm{d}s'\mathrm{d}s \\
&= \frac{A^{(i,j)}(x)\,h_n}{2\rho^{(i)}\rho^{(j)}}\left(p^2 - \left(p-\rho^{(i)}\right)^2\right) \\
&\quad - \frac{A^{(i,j)}(x)\left(p+1-\rho^{(j)}\right)h_n}{2\rho^{(i)}\rho^{(j)}}\left(p^2 - \left(p-\rho^{(i)}\right)^2\right) \\
&\quad + \frac{A^{(i,j)}(x)\,h_n}{6\rho^{(i)}\rho^{(j)}}\left(p^3 - \left(p-\rho^{(i)}\right)^3\right) \\
&\quad + \frac{A^{(i,j)}(x)\left(p-\rho^{(j)}\right)^2 h_n}{2\rho^{(i)}\rho^{(j)}}\left(p - \left(p-\rho^{(i)}\right)\right) \\
&= \frac{A^{(i,j)}(x)\,h_n}{6\rho^{(i)}\rho^{(j)}}\left[3\left(p^2 - \left(p-\rho^{(i)}\right)^2\right) - 3\left(p+1-\rho^{(j)}\right)\left(p^2 - \left(p-\rho^{(i)}\right)^2\right)\right. \\
&\qquad\qquad \left. + \left(p^3 - \left(p-\rho^{(i)}\right)^3\right) + 3\left(p-\rho^{(j)}\right)^2\left(p - \left(p-\rho^{(i)}\right)\right)\right] \\
&= \frac{A^{(i,j)}(x)\,h_n}{6\rho^{(i)}\rho^{(j)}}\left[\left(\rho^{(i)} - \rho^{(j)}\right)^3 + \left(\rho^{(j)}\right)^3\right]
\end{aligned}$$

and for the case (v),

$$\begin{aligned}
& h_n \mathbf{D}_0^{(i,j)}(x|\rho) \\
&= \int_0^{(p+1)h_n}\int_0^{(p+1)h_n} A^{(i,j)}(x)\min\{s,s'\} \\
&\qquad \left(V_{\rho,h_n}^{(i,i)}(ph_n - s)\left(V_{\rho,h_n}^{(j,j)}((p+1)h_n - s') - V_{\rho,h_n}^{(j,j)}(ph_n - s')\right)\right)\mathrm{d}s'\mathrm{d}s \\
&= \frac{A^{(i,j)}(x)\,h_n}{2\rho^{(i)}\rho^{(j)}}\left(p^2 - \left(p-\rho^{(i)}\right)^2\right) \\
&\quad - \frac{A^{(i,j)}(x)\left(p+1-\rho^{(j)}\right)h_n}{2\rho^{(i)}\rho^{(j)}}\left(p^2 - \left(p-\rho^{(i)}\right)^2\right) \\
&\quad + \frac{A^{(i,j)}(x)\,h_n}{6\rho^{(i)}\rho^{(j)}}\left(p^3 - \left(p-\rho^{(j)}\right)^3\right) \\
&\quad + \frac{A^{(i,j)}(x)\left(p-\rho^{(j)}\right)h_n}{2\rho^{(i)}\rho^{(j)}}\left(\left(p-\rho^{(j)}\right)^2 - \left(p-\rho^{(i)}\right)^2\right) \\
&\quad + \frac{A^{(i,j)}(x)\left(p-\rho^{(j)}\right)^2 h_n}{2\rho^{(i)}\rho^{(j)}}\left(p - \left(p-\rho^{(j)}\right)\right) \\
&= \frac{A^{(i,j)}(x)\,h_n}{6\rho^{(i)}\rho^{(j)}}\left[3\left(p^2 - \left(p-\rho^{(i)}\right)^2\right)\right. \\
&\qquad\qquad - 3\left(p+1-\rho^{(j)}\right)\left(p^2 - \left(p-\rho^{(i)}\right)^2\right) + \left(p^3 - \left(p-\rho^{(j)}\right)^3\right)
\end{aligned}$$

$$+3\left(p-\rho^{(j)}\right)\left(\left(p-\rho^{(j)}\right)^2-\left(p-\rho^{(i)}\right)^2\right)+3\left(p-\rho^{(j)}\right)^2\left(p-\left(p-\rho^{(j)}\right)\right)\Big]$$
$$=\frac{A^{(i,j)}(x)\,h_n}{6\rho^{(i)}\rho^{(j)}}\left(\rho^{(j)}\right)^3.$$

Hence, it follows from (i)-(v) that $\mathbf{D}_0(x|\rho)=\mathbb{D}_0(x|\rho)$. Regarding $D_\ell(x)$ where $\ell\geq\left[\max_{i=1,\dots,n}\rho^{(i)}\right]+1$, it is obvious that

$$\mathbf{E}\left[\left(\int_0^{(p+1+\ell)h_n}V_{\rho,h_n}(ph_n-s_1)\left(\int_0^{s_1}a(x)\,\mathrm{d}w_{s_2}\right)\mathrm{d}s_1\right)\right.$$
$$\left(\int_0^{(p+1+\ell)h_n}\left(V_{\rho,h_n}((p+1+\ell)h_n-s_1)-V_{\rho,h_n}((p+\ell)h_n-s_1)\right)\right.$$
$$\left.\left.\times\left(\int_0^{s_1}a(x)\,\mathrm{d}w_{s_2}\right)\mathrm{d}s_1\right)^T\right]=O$$

With respect to $G(x)$, for all $i,j=1,\dots,d$, let us define $\mathbf{G}^{(i,j)}(x|\rho)$ such that

$$\mathbf{G}^{(i,j)}(x|\rho)$$
$$:=\frac{1}{h_n}\mathbf{E}\left[\left(\int_0^{\Delta_n+h_n}\left(\Phi_{\Delta_n,n}((\Delta_n+h_n)-s_1)-\Phi_{\Delta_n,n}(\Delta_n-s_1)\right)\left(\int_0^{s_1}a(x)\,\mathrm{d}w_{s_2}\right)\mathrm{d}s_1\right)\right.$$
$$\left.\left(\int_0^{\Delta_n+h_n}\left(\Phi_{\Delta_n,n}((\Delta_n+h_n)-s_1)-\Phi_{\Delta_n,n}(\Delta_n-s_1)\right)\left(\int_0^{s_1}a(x)\,\mathrm{d}w_{s_2}\right)\mathrm{d}s_1\right)^T\right]^{(i,j)}$$
$$=\frac{1}{h_n}\int_0^{(p+1)h_n}\int_0^{(p+1)h_n}A^{(i,j)}(x)\min\{s,s'\}\left(V_{\rho,h_n}^{(i,i)}((p+1)h_n-s)-V_{\rho,h_n}^{(i,i)}(ph_n-s)\right)$$
$$\left(V_{\rho,h_n}^{(j,j)}((p+1)h_n-s')-V_{\rho,h_n}^{(j,j)}(ph_n-s')\right)\mathrm{d}s'\mathrm{d}s$$
$$=\frac{1}{h_n}\int_0^{(p+1)h_n}\int_0^{(p+1)h_n}A^{(i,j)}(x)\min\{s,s'\}\left(V_{\rho,h_n}^{(i,i)}((p+1)h_n-s)\right)$$
$$\left(V_{\rho,h_n}^{(j,j)}((p+1)h_n-s')-V_{\rho,h_n}^{(j,j)}(ph_n-s')\right)\mathrm{d}s'\mathrm{d}s-\mathbf{D}_0^{(i,j)}(x|\rho),$$

and $\mathbf{K}^{(i,j)}(x|\rho):=\mathbf{G}^{(i,j)}(x|\rho)+\mathbf{D}_0^{(i,j)}(x|\rho)$. If $\rho^{(i)}=0$, as evaluation of B,

$$h_n\mathbf{K}^{(i,j)}(x|\rho)$$
$$=\int_0^{(p+1)h_n}\int_0^{(p+1)h_n}A^{(i,j)}(x)\min\{s,s'\}\left(V_{\rho,h_n}^{(i,i)}((p+1)h_n-s)\right)$$
$$\left(V_{\rho,h_n}^{(j,j)}((p+1)h_n-s')-V_{\rho,h_n}^{(j,j)}(ph_n-s')\right)\mathrm{d}s'\mathrm{d}s$$
$$=A^{(i,j)}(x)\int_0^{(p+1)h_n}\left(V_{\rho,h_n}^{(j,j)}((p+1)h_n-s')-V_{\rho,h_n}^{(j,j)}(ph_n-s')\right)s'\mathrm{d}s'$$
$$=h_nA^{(i,j)}(x),$$

and if $\rho^{(i)} \in (0,1]$ and $\rho^{(j)} = 0$,

$$\begin{aligned}
&h_n \mathbf{K}^{(i,j)}(x|\rho) \\
&= \int_0^{(p+1)h_n} \int_0^{(p+1)h_n} A^{(i,j)}(x) \min\{s,s'\} \left(V_{\rho,h_n}^{(i,i)}((p+1)h_n - s)\right) \\
&\qquad \left(V_{\rho,h_n}^{(j,j)}((p+1)h_n - s') - V_{\rho,h_n}^{(j,j)}(ph_n - s')\right) \mathrm{d}s'\mathrm{d}s \\
&= \int_0^{(p+1)h_n} \int_0^{(p+1)h_n} A^{(i,j)}(x) \min\{s,s'\} \left(V_{\rho,h_n}^{(i,i)}((p+1)h_n - s)\right) \\
&\qquad \times V_{\rho,h_n}^{(j,j)}((p+1)h_n - s') \mathrm{d}s'\mathrm{d}s \\
&\quad - \int_0^{(p+1)h_n} \int_0^{(p+1)h_n} A^{(i,j)}(x) \min\{s,s'\} \left(V_{\rho,h_n}^{(i,i)}((p+1)h_n - s)\right) \\
&\qquad \times V_{\rho,h_n}^{(j,j)}(ph_n - s') \mathrm{d}s'\mathrm{d}s \\
&= A^{(i,j)}(x) \int_0^{(p+1)h_n} s \left(V_{\rho,h_n}^{(i,i)}((p+1)h_n - s)\right) \mathrm{d}s \\
&\quad - A^{(i,j)}(x) \int_0^{(p+1)h_n} \min\{s, ph_n\} \left(V_{\rho,h_n}^{(i,i)}((p+1)h_n - s)\right) \mathrm{d}s \\
&= \frac{A^{(i,j)}(x)}{\rho^{(i)} h_n} \left(\int_{\left(p+1-\rho^{(i)}\right)h_n}^{(p+1)h_n} s\mathrm{d}s - \int_{\left(p+1-\rho^{(i)}\right)h_n}^{(p+1)h_n} \min\{s, ph_n\} \mathrm{d}s \right) \\
&= \frac{A^{(i,j)}(x)}{\rho^{(i)} h_n} \left(\int_{\left(p+1-\rho^{(i)}\right)h_n}^{(p+1)h_n} s\mathrm{d}s - \int_{\left(p+1-\rho^{(i)}\right)h_n}^{(p+1)h_n} ph_n \mathrm{d}s \right) \\
&= \frac{A^{(i,j)}(x)}{\rho^{(i)} h_n} \left(\frac{1}{2}(p+1)^2 h_n^2 - \frac{1}{2}\left(p+1-\rho^{(i)}\right)^2 h_n^2 - p(p+1)h_n^2 + p\left(p+1-\rho^{(i)}\right)h_n^2 \right) \\
&= h_n A^{(i,j)}(x) \left(1 - \frac{\rho^{(i)}}{2}\right)
\end{aligned}$$

and if $\rho^{(i)} \in (1, \overline{\rho}]$ and $\rho^{(j)} = 0$,

$$\begin{aligned}
&h_n \mathbf{K}^{(i,j)}(x|\rho) \\
&= \int_0^{(p+1)h_n} \int_0^{(p+1)h_n} A^{(i,j)}(x) \min\{s,s'\} \left(V_{\rho,h_n}^{(i,i)}((p+1)h_n - s)\right) \\
&\qquad \left(V_{\rho,h_n}^{(j,j)}((p+1)h_n - s') - V_{\rho,h_n}^{(j,j)}(ph_n - s')\right) \mathrm{d}s'\mathrm{d}s \\
&= \frac{A^{(i,j)}(x)}{\rho^{(i)} h_n} \left(\int_{\left(p+1-\rho^{(i)}\right)h_n}^{(p+1)h_n} s\mathrm{d}s - \int_{\left(p+1-\rho^{(i)}\right)h_n}^{(p+1)h_n} \min\{s, ph_n\} \mathrm{d}s \right) \\
&= \frac{A^{(i,j)}(x)}{\rho^{(i)} h_n} \left(\int_{\left(p+1-\rho^{(i)}\right)h_n}^{(p+1)h_n} s\mathrm{d}s - \int_{ph_n}^{(p+1)h_n} ph_n \mathrm{d}s - \int_{\left(p+1-\rho^{(i)}\right)h_n}^{ph_n} s\mathrm{d}s \right) \\
&= \frac{A^{(i,j)}(x)}{\rho^{(i)} h_n} \left(\frac{1}{2}(p+1)^2 h_n^2 - \frac{1}{2}\left(p+1-\rho^{(i)}\right)^2 h_n^2 - p(p+1)h_n^2 \right. \\
&\qquad \left. + p^2 h_n^2 - \frac{1}{2} p^2 h_n^2 + \frac{1}{2}\left(p+1-\rho^{(i)}\right)^2 h_n^2 \right)
\end{aligned}$$

$$= \frac{h_n A^{(i,j)}(x)}{2\rho^{(i)}}$$

and if $\rho^{(i)} > 0$ and $\rho^{(j)} > 0$,

$$\begin{aligned}
&h_n \mathbf{K}^{(i,j)}(x|\rho) \\
&= \int_0^{(p+1)h_n} \int_0^{(p+1)h_n} A^{(i,j)}(x) \min\{s,s'\} \left(V_{\rho,h_n}^{(i,i)}((p+1)h_n - s)\right) \\
&\qquad \left(V_{\rho,h_n}^{(j,j)}((p+1)h_n - s') - V_{\rho,h_n}^{(j,j)}(ph_n - s')\right) \mathrm{d}s'\mathrm{d}s \\
&= \frac{A^{(i,j)}(x)}{\rho^{(i)}\rho^{(j)}h_n^2} \int_0^{(p+1)h_n} \int_0^{(p+1)h_n} \min\{s,s'\} \left(\mathbf{1}_{\left[0,\rho^{(i)}h_n\right]}((p+1)h_n - s)\right) \\
&\qquad \left(\mathbf{1}_{\left[0,\rho^{(j)}h_n\right]}((p+1)h_n - s') - \mathbf{1}_{\left[0,\rho^{(j)}h_n\right]}(ph_n - s')\right) \mathrm{d}s'\mathrm{d}s \\
&= \frac{A^{(i,j)}(x)}{\rho^{(i)}\rho^{(j)}h_n^2} \int_0^{(p+1)h_n} \int_s^{(p+1)h_n} s \left(\mathbf{1}_{\left[0,\rho^{(i)}h_n\right]}((p+1)h_n - s)\right) \\
&\qquad \left(\mathbf{1}_{\left[0,\rho^{(j)}h_n\right]}((p+1)h_n - s') - \mathbf{1}_{\left[0,\rho^{(j)}h_n\right]}(ph_n - s')\right) \mathrm{d}s'\mathrm{d}s \\
&\quad + \frac{A^{(i,j)}(x)}{\rho^{(i)}\rho^{(j)}h_n^2} \int_0^{(p+1)h_n} \int_0^{s} s' \left(\mathbf{1}_{\left[0,\rho^{(i)}h_n\right]}((p+1)h_n - s)\right) \\
&\qquad \left(\mathbf{1}_{\left[0,\rho^{(j)}h_n\right]}((p+1)h_n - s') - \mathbf{1}_{\left[0,\rho^{(j)}h_n\right]}(ph_n - s')\right) \mathrm{d}s'\mathrm{d}s \\
&= \frac{A^{(i,j)}(x)}{\rho^{(i)}\rho^{(j)}h_n^2} \int_0^{(p+1)h_n} s \left(\mathbf{1}_{\left[0,\rho^{(i)}h_n\right]}((p+1)h_n - s)\right) \\
&\qquad \int_s^{(p+1)h_n} \left(\mathbf{1}_{\left[0,\rho^{(j)}h_n\right]}((p+1)h_n - s')\right) \mathrm{d}s'\mathrm{d}s \\
&\quad - \frac{A^{(i,j)}(x)}{\rho^{(i)}\rho^{(j)}h_n^2} \int_0^{(p+1)h_n} s \left(\mathbf{1}_{\left[0,\rho^{(i)}h_n\right]}((p+1)h_n - s)\right) \\
&\qquad \int_s^{(p+1)h_n} \left(\mathbf{1}_{\left[0,\rho^{(j)}h_n\right]}(ph_n - s')\right) \mathrm{d}s'\mathrm{d}s \\
&\quad + \frac{A^{(i,j)}(x)}{\rho^{(i)}\rho^{(j)}h_n^2} \int_0^{(p+1)h_n} \left(\mathbf{1}_{\left[0,\rho^{(i)}h_n\right]}((p+1)h_n - s)\right) \\
&\qquad \int_0^{s} s' \left(\mathbf{1}_{\left[0,\rho^{(j)}h_n\right]}((p+1)h_n - s')\right) \mathrm{d}s'\mathrm{d}s \\
&\quad - \frac{A^{(i,j)}(x)}{\rho^{(i)}\rho^{(j)}h_n^2} \int_0^{(p+1)h_n} \left(\mathbf{1}_{\left[0,\rho^{(i)}h_n\right]}((p+1)h_n - s)\right) \\
&\qquad \int_0^{s} s' \left(\mathbf{1}_{\left[0,\rho^{(j)}h_n\right]}(ph_n - s')\right) \mathrm{d}s'\mathrm{d}s \\
&= \frac{A^{(i,j)}(x)}{\rho^{(i)}\rho^{(j)}h_n^2} \int_0^{(p+1)h_n} s \left(\mathbf{1}_{\left[0,\rho^{(i)}h_n\right]}((p+1)h_n - s)\right) \int_{\max\left\{s,\left(p+1-\rho^{(j)}\right)h_n\right\}}^{(p+1)h_n} \mathrm{d}s'\mathrm{d}s \\
&\quad - \frac{A^{(i,j)}(x)}{\rho^{(i)}\rho^{(j)}h_n^2} \int_0^{(p+1)h_n} s \left(\mathbf{1}_{\left[0,\rho^{(i)}h_n\right]}((p+1)h_n - s)\right)
\end{aligned}$$

$$\left(\mathbf{1}_{[0,ph_n]}(s)\right)\int_{\max\left\{s,\left(p-\rho^{(j)}\right)h_n\right\}}^{ph_n}\mathrm{d}s'\mathrm{d}s$$

$$+\frac{A^{(i,j)}(x)}{\rho^{(i)}\rho^{(j)}h_n^2}\int_0^{(p+1)h_n}\left(\mathbf{1}_{\left[0,\rho^{(i)}h_n\right]}((p+1)h_n-s)\right)\left(\mathbf{1}_{\left[\left(p+1-\rho^{(j)}\right)h_n,(p+1)h_n\right]}(s)\right)$$

$$\int_{\left(p+1-\rho^{(j)}\right)h_n}^{s}s'\mathrm{d}s'\mathrm{d}s$$

$$-\frac{A^{(i,j)}(x)}{\rho^{(i)}\rho^{(j)}h_n^2}\int_0^{(p+1)h_n}\left(\mathbf{1}_{\left[0,\rho^{(i)}h_n\right]}((p+1)h_n-s)\right)\left(\mathbf{1}_{\left[\left(p-\rho^{(j)}\right)h_n,(p+1)h_n\right]}(s)\right)$$

$$\int_{\left(p-\rho^{(j)}\right)h_n}^{\min\{s,ph_n\}}s'\mathrm{d}s'\mathrm{d}s$$

$$=\frac{A^{(i,j)}(x)}{\rho^{(i)}\rho^{(j)}h_n^2}\int_{\left(p+1-\rho^{(i)}\right)h_n}^{(p+1)h_n}s\left((p+1)h_n-\max\left\{s,\left(p+1-\rho^{(j)}\right)h_n\right\}\right)\mathrm{d}s$$

$$-\frac{A^{(i,j)}(x)}{\rho^{(i)}\rho^{(j)}h_n^2}\mathbf{1}_{(1,\bar{\rho}]}\left(\rho^{(i)}\right)\int_{\left(p+1-\rho^{(i)}\right)h_n}^{ph_n}s\left(ph_n-\max\left\{s,\left(p-\rho^{(j)}\right)h_n\right\}\right)\mathrm{d}s$$

$$+\frac{A^{(i,j)}(x)}{\rho^{(i)}\rho^{(j)}h_n^2}\int_{\max\left\{\left(p+1-\rho^{(i)}\right)h_n,\left(p+1-\rho^{(j)}\right)h_n\right\}}^{(p+1)h_n}\left(\frac{s^2}{2}-\frac{\left(p+1-\rho^{(j)}\right)^2h_n^2}{2}\right)\mathrm{d}s$$

$$-\frac{A^{(i,j)}(x)}{\rho^{(i)}\rho^{(j)}h_n^2}\int_{\max\left\{\left(p+1-\rho^{(i)}\right)h_n,\left(p-\rho^{(j)}\right)h_n\right\}}^{(p+1)h_n}\left(\frac{\min\left\{s^2,p^2h_n^2\right\}}{2}-\frac{\left(p-\rho^{(j)}\right)^2h_n^2}{2}\right)\mathrm{d}s$$

$$=\frac{A^{(i,j)}(x)}{\rho^{(i)}\rho^{(j)}h_n^2}\int_{\max\left\{\left(p+1-\rho^{(i)}\right)h_n,\left(p+1-\rho^{(j)}\right)h_n\right\}}^{(p+1)h_n}s\left((p+1)h_n-s\right)\mathrm{d}s$$

$$+\frac{A^{(i,j)}(x)}{\rho^{(i)}\rho^{(j)}h_n^2}\mathbf{1}_{\left(\rho^{(j)},\bar{\rho}\right]}\left(\rho^{(i)}\right)\int_{\left(p+1-\rho^{(i)}\right)h_n}^{\left(p+1-\rho^{(j)}\right)h_n}s\left((p+1)h_n-\left(p+1-\rho^{(j)}\right)h_n\right)\mathrm{d}s$$

$$-\frac{A^{(i,j)}(x)}{\rho^{(i)}\rho^{(j)}h_n^2}\mathbf{1}_{(1,\bar{\rho}]}\left(\rho^{(i)}\right)\int_{\max\left\{\left(p+1-\rho^{(i)}\right)h_n,\left(p-\rho^{(j)}\right)h_n\right\}}^{ph_n}s\left(ph_n-s\right)\mathrm{d}s$$

$$-\frac{A^{(i,j)}(x)}{\rho^{(i)}\rho^{(j)}h_n^2}\mathbf{1}_{\left(\rho^{(j)}+1,\bar{\rho}\right]}\left(\rho^{(i)}\right)\int_{\left(p+1-\rho^{(i)}\right)h_n}^{\left(p-\rho^{(j)}\right)h_n}s\left(ph_n-\left(p-\rho^{(j)}\right)h_n\right)\mathrm{d}s$$

$$+\frac{A^{(i,j)}(x)}{\rho^{(i)}\rho^{(j)}h_n^2}\int_{\max\left\{\left(p+1-\rho^{(i)}\right)h_n,\left(p+1-\rho^{(j)}\right)h_n\right\}}^{(p+1)h_n}\left(\frac{s^2}{2}-\frac{\left(p+1-\rho^{(j)}\right)^2h_n^2}{2}\right)\mathrm{d}s$$

$$-\frac{A^{(i,j)}(x)}{\rho^{(i)}\rho^{(j)}h_n^2}\mathbf{1}_{(1,\bar{\rho}]}\left(\rho^{(i)}\right)\int_{ph_n}^{(p+1)h_n}\left(\frac{p^2h_n^2}{2}-\frac{\left(p-\rho^{(j)}\right)^2h_n^2}{2}\right)\mathrm{d}s$$

$$-\frac{A^{(i,j)}(x)}{\rho^{(i)}\rho^{(j)}h_n^2}\mathbf{1}_{(1,\bar{\rho}]}\left(\rho^{(i)}\right)\int_{\max\left\{\left(p+1-\rho^{(i)}\right)h_n,\left(p-\rho^{(j)}\right)h_n\right\}}^{ph_n}\left(\frac{s^2}{2}-\frac{\left(p-\rho^{(j)}\right)^2h_n^2}{2}\right)\mathrm{d}s$$

$$-\frac{A^{(i,j)}(x)}{\rho^{(i)}\rho^{(j)}h_n^2}\mathbf{1}_{(0,1]}\left(\rho^{(i)}\right)\int_{\left(p+1-\rho^{(i)}\right)h_n}^{(p+1)h_n}\left(\frac{p^2h_n^2}{2}-\frac{\left(p-\rho^{(j)}\right)^2h_n^2}{2}\right)\mathrm{d}s$$

$$
\begin{aligned}
&= \frac{A^{(i,j)}(x)}{\rho^{(i)}\rho^{(j)}h_n^2}\left(\frac{(p+1)^2 h_n^2}{2} - \frac{\max\left\{\left(p+1-\rho^{(i)}\right)^2 h_n^2, \left(p+1-\rho^{(j)}\right)^2 h_n^2\right\}}{2}\right)((p+1)h_n)\\
&- \frac{A^{(i,j)}(x)}{\rho^{(i)}\rho^{(j)}h_n^2}\left(\frac{(p+1)^3 h_n^3}{3} - \frac{\max\left\{\left(p+1-\rho^{(i)}\right)^3 h_n^3, \left(p+1-\rho^{(j)}\right)^3 h_n^3\right\}}{3}\right)\\
&+ \frac{A^{(i,j)}(x)}{\rho^{(i)}\rho^{(j)}h_n^2}\mathbf{1}_{\left(\rho^{(j)},\bar{\rho}\right]}\left(\rho^{(i)}\right)\left(\frac{\left(p+1-\rho^{(j)}\right)^2 h_n^2}{2} - \frac{\left(p+1-\rho^{(i)}\right)^2 h_n^2}{2}\right)\left(\rho^{(j)}h_n\right)\\
&- \frac{A^{(i,j)}(x)}{\rho^{(i)}\rho^{(j)}h_n^2}\mathbf{1}_{(1,\bar{\rho}]}\left(\rho^{(i)}\right)\left(\frac{p^2h_n^2}{2} - \frac{\max\left\{\left(p+1-\rho^{(i)}\right)^2 h_n^2, \left(p-\rho^{(j)}\right)^2 h_n^2\right\}}{2}\right)(ph_n)\\
&+ \frac{A^{(i,j)}(x)}{\rho^{(i)}\rho^{(j)}h_n^2}\mathbf{1}_{(1,\bar{\rho}]}\left(\rho^{(i)}\right)\left(\frac{p^3h_n^3}{3} - \frac{\max\left\{\left(p+1-\rho^{(i)}\right)^3 h_n^3, \left(p-\rho^{(j)}\right)^3 h_n^3\right\}}{3}\right)\\
&- \frac{A^{(i,j)}(x)}{\rho^{(i)}\rho^{(j)}h_n^2}\mathbf{1}_{\left(\rho^{(j)}+1,\bar{\rho}\right]}\left(\rho^{(i)}\right)\left(\frac{\left(p-\rho^{(j)}\right)^2 h_n^2}{2} - \frac{\left(p+1-\rho^{(i)}\right)^2 h_n^2}{2}\right)\left(\rho^{(j)}h_n\right)\\
&+ \frac{A^{(i,j)}(x)}{\rho^{(i)}\rho^{(j)}h_n^2}\left(\frac{(p+1)^3 h_n^3}{6} - \frac{\max\left\{\left(p+1-\rho^{(i)}\right)^3 h_n^3, \left(p+1-\rho^{(j)}\right)^3 h_n^3\right\}}{6}\right)\\
&- \frac{A^{(i,j)}(x)}{\rho^{(i)}\rho^{(j)}h_n^2}\left(\min\left\{\rho^{(i)}h_n, \rho^{(j)}h_n\right\}\right)\left(\frac{\left(p+1-\rho^{(j)}\right)^2 h_n^2}{2}\right)\\
&- \frac{A^{(i,j)}(x)}{\rho^{(i)}\rho^{(j)}h_n^2}\mathbf{1}_{(1,\bar{\rho}]}\left(\rho^{(i)}\right)\left(\frac{p^2h_n^2}{2} - \frac{\left(p-\rho^{(j)}\right)^2 h_n^2}{2}\right)h_n\\
&- \frac{A^{(i,j)}(x)}{\rho^{(i)}\rho^{(j)}h_n^2}\mathbf{1}_{(1,\bar{\rho}]}\left(\rho^{(i)}\right)\left(\frac{p^3h_n^3}{6} - \frac{\max\left\{\left(p+1-\rho^{(i)}\right)^3 h_n^3, \left(p-\rho^{(j)}\right)^3 h_n^3\right\}}{6}\right)\\
&+ \frac{A^{(i,j)}(x)}{\rho^{(i)}\rho^{(j)}h_n^2}\mathbf{1}_{(1,\bar{\rho}]}\left(\rho^{(i)}\right)\left(ph_n - \max\left\{\left(p+1-\rho^{(i)}\right)h_n, \left(p-\rho^{(j)}\right)h_n\right\}\right)\left(\frac{\left(p-\rho^{(j)}\right)^2 h_n^2}{2}\right)\\
&- \frac{A^{(i,j)}(x)}{\rho^{(i)}\rho^{(j)}h_n^2}\mathbf{1}_{(0,1]}\left(\rho^{(i)}\right)\left(\frac{p^2h_n^2}{2} - \frac{\left(p-\rho^{(j)}\right)^2 h_n^2}{2}\right)\left(\rho^{(i)}h_n\right),
\end{aligned}
$$

and we consider the following cases: (i) $\rho^{(i)} > 1$ and $\rho^{(i)} > \rho^{(j)} + 1$; (ii) $\rho^{(i)} > 1$ and $\rho^{(j)} < \rho^{(i)} \leq \rho^{(j)} + 1$; (iii) $\rho^{(i)} > 1$ and $\rho^{(i)} \leq \rho^{(j)}$; (iv) $\rho^{(i)} \leq 1$ and $\rho^{(j)} < \rho^{(i)}$; (v) $\rho^{(i)} \leq 1$ and $\rho^{(i)} \leq \rho^{(j)}$, and for the case (i),

$$
\begin{aligned}
&h_n\mathbf{K}^{(i,j)}(x|\rho)\\
&= \int_0^{(p+1)h_n}\int_0^{(p+1)h_n} A^{(i,j)}(x)\min\{s,s'\}\left(V_{\rho,h_n}^{(i,i)}((p+1)h_n - s)\right)\\
&\qquad \left(V_{\rho,h_n}^{(j,j)}((p+1)h_n - s') - V_{\rho,h_n}^{(j,j)}(ph_n - s')\right)\mathrm{d}s'\mathrm{d}s
\end{aligned}
$$

$$
\begin{aligned}
&= \frac{A^{(i,j)}(x)}{\rho^{(i)}\rho^{(j)}h_n^2}\left(\frac{(p+1)^2 h_n^2}{2} - \frac{\left(p+1-\rho^{(j)}\right)^2 h_n^2}{2}\right)((p+1)h_n) \\
&\quad - \frac{A^{(i,j)}(x)}{\rho^{(i)}\rho^{(j)}h_n^2}\left(\frac{(p+1)^3 h_n^3}{3} - \frac{\left(p+1-\rho^{(j)}\right)^3 h_n^3}{3}\right) \\
&\quad + \frac{A^{(i,j)}(x)}{\rho^{(i)}\rho^{(j)}h_n^2}\mathbf{1}_{\left(\rho^{(j)},\overline{\rho}\right]}\left(\rho^{(i)}\right)\left(\frac{\left(p+1-\rho^{(j)}\right)^2 h_n^2}{2} - \frac{\left(p+1-\rho^{(i)}\right)^2 h_n^2}{2}\right)\left(\rho^{(j)}h_n\right) \\
&\quad - \frac{A^{(i,j)}(x)}{\rho^{(i)}\rho^{(j)}h_n^2}\mathbf{1}_{(1,\overline{\rho}]}\left(\rho^{(i)}\right)\left(\frac{p^2 h_n^2}{2} - \frac{\left(p-\rho^{(j)}\right)^2 h_n^2}{2}\right)(ph_n) \\
&\quad + \frac{A^{(i,j)}(x)}{\rho^{(i)}\rho^{(j)}h_n^2}\mathbf{1}_{(1,\overline{\rho}]}\left(\rho^{(i)}\right)\left(\frac{p^3 h_n^3}{3} - \frac{\left(p-\rho^{(j)}\right)^3 h_n^3}{3}\right) \\
&\quad - \frac{A^{(i,j)}(x)}{\rho^{(i)}\rho^{(j)}h_n^2}\mathbf{1}_{\left(\rho^{(j)}+1,\overline{\rho}\right]}\left(\rho^{(i)}\right)\left(\frac{\left(p-\rho^{(j)}\right)^2 h_n^2}{2} - \frac{\left(p+1-\rho^{(i)}\right)^2 h_n^2}{2}\right)\left(\rho^{(j)}h_n\right) \\
&\quad + \frac{A^{(i,j)}(x)}{\rho^{(i)}\rho^{(j)}h_n^2}\left(\frac{(p+1)^3 h_n^3}{6} - \frac{\left(p+1-\rho^{(j)}\right)^3 h_n^3}{6}\right) \\
&\quad - \frac{A^{(i,j)}(x)}{\rho^{(i)}\rho^{(j)}h_n^2}\left(\rho^{(j)}h_n\right)\left(\frac{\left(p+1-\rho^{(j)}\right)^2 h_n^2}{2}\right) \\
&\quad - \frac{A^{(i,j)}(x)}{\rho^{(i)}\rho^{(j)}h_n^2}\mathbf{1}_{(1,\overline{\rho}]}\left(\rho^{(i)}\right)\left(\frac{p^2 h_n^2}{2} - \frac{\left(p-\rho^{(j)}\right)^2 h_n^2}{2}\right)h_n \\
&\quad - \frac{A^{(i,j)}(x)}{\rho^{(i)}\rho^{(j)}h_n^2}\mathbf{1}_{(1,\overline{\rho}]}\left(\rho^{(i)}\right)\left(\frac{p^3 h_n^3}{6} - \frac{\left(p-\rho^{(j)}\right)^3 h_n^3}{6}\right) \\
&\quad + \frac{A^{(i,j)}(x)}{\rho^{(i)}\rho^{(j)}h_n^2}\mathbf{1}_{(1,\overline{\rho}]}\left(\rho^{(i)}\right)\left(\rho^{(j)}h_n\right)\left(\frac{\left(p-\rho^{(j)}\right)^2 h_n^2}{2}\right) \\
&= \frac{h_n A^{(i,j)}(x)}{\rho^{(i)}\rho^{(j)}}\left(\frac{\left(\rho^{(j)}\right)^2}{2} + \frac{\rho^{(j)}}{2}\right),
\end{aligned}
$$

and for the case (ii),

$$
\begin{aligned}
&h_n\mathbf{K}^{(i,j)}(x|\rho) \\
&= \int_0^{(p+1)h_n}\int_0^{(p+1)h_n} A^{(i,j)}(x)\min\{s,s'\}\left(V_{\rho,h_n}^{(i,i)}((p+1)h_n - s)\right) \\
&\qquad\qquad \left(V_{\rho,h_n}^{(j,j)}((p+1)h_n - s') - V_{\rho,h_n}^{(j,j)}(ph_n - s')\right)\mathrm{d}s'\mathrm{d}s \\
&= \frac{A^{(i,j)}(x)}{\rho^{(i)}\rho^{(j)}h_n^2}\left(\frac{(p+1)^2 h_n^2}{2} - \frac{\left(p+1-\rho^{(j)}\right)^2 h_n^2}{2}\right)((p+1)h_n) \\
&\quad - \frac{A^{(i,j)}(x)}{\rho^{(i)}\rho^{(j)}h_n^2}\left(\frac{(p+1)^3 h_n^3}{3} - \frac{\left(p+1-\rho^{(j)}\right)^3 h_n^3}{3}\right)
\end{aligned}
$$

$$
\begin{aligned}
&+\frac{A^{(i,j)}(x)}{\rho^{(i)}\rho^{(j)}h_n^2}\mathbf{1}_{(\rho^{(j)},\bar{\rho}]}\left(\rho^{(i)}\right)\left(\frac{\left(p+1-\rho^{(j)}\right)^2h_n^2}{2}-\frac{\left(p+1-\rho^{(i)}\right)^2h_n^2}{2}\right)\left(\rho^{(j)}h_n\right)\\
&-\frac{A^{(i,j)}(x)}{\rho^{(i)}\rho^{(j)}h_n^2}\mathbf{1}_{(1,\bar{\rho}]}\left(\rho^{(i)}\right)\left(\frac{p^2h_n^2}{2}-\frac{\left(p+1-\rho^{(i)}\right)^2h_n^2}{2}\right)(ph_n)\\
&+\frac{A^{(i,j)}(x)}{\rho^{(i)}\rho^{(j)}h_n^2}\mathbf{1}_{(1,\bar{\rho}]}\left(\rho^{(i)}\right)\left(\frac{p^3h_n^3}{3}-\frac{\left(p+1-\rho^{(i)}\right)^3h_n^3}{3}\right)\\
&+\frac{A^{(i,j)}(x)}{\rho^{(i)}\rho^{(j)}h_n^2}\left(\frac{(p+1)^3h_n^3}{6}-\frac{\left(p+1-\rho^{(j)}\right)^3h_n^3}{6}\right)\\
&-\frac{A^{(i,j)}(x)}{\rho^{(i)}\rho^{(j)}h_n^2}\left(\rho^{(j)}h_n\right)\left(\frac{\left(p+1-\rho^{(j)}\right)^2h_n^2}{2}\right)\\
&-\frac{A^{(i,j)}(x)}{\rho^{(i)}\rho^{(j)}h_n^2}\mathbf{1}_{(1,\bar{\rho}]}\left(\rho^{(i)}\right)\left(\frac{p^2h_n^2}{2}-\frac{\left(p-\rho^{(j)}\right)^2h_n^2}{2}\right)h_n\\
&-\frac{A^{(i,j)}(x)}{\rho^{(i)}\rho^{(j)}h_n^2}\mathbf{1}_{(1,\bar{\rho}]}\left(\rho^{(i)}\right)\left(\frac{p^3h_n^3}{6}-\frac{\left(p+1-\rho^{(i)}\right)^3h_n^3}{6}\right)\\
&+\frac{A^{(i,j)}(x)}{\rho^{(i)}\rho^{(j)}h_n^2}\mathbf{1}_{(1,\bar{\rho}]}\left(\rho^{(i)}\right)\left(\left(\rho^{(i)}-1\right)h_n\right)\left(\frac{\left(p-\rho^{(j)}\right)^2h_n^2}{2}\right)\\
=&\frac{h_nA^{(i,j)}(x)}{6\rho^{(i)}\rho^{(j)}}\\
&\times\left(\left(\rho^{(i)}\right)^3-3\left(\rho^{(i)}\right)^2\rho^{(j)}-3\left(\rho^{(i)}\right)^2+3\rho^{(i)}\left(\rho^{(j)}\right)^2+6\rho^{(i)}\rho^{(j)}+3\rho^{(i)}-\left(\rho^{(j)}\right)^3-1\right)
\end{aligned}
$$

and for the case (iii),

$$
\begin{aligned}
&h_n\mathbf{K}^{(i,j)}(x|\rho)\\
&=\int_0^{(p+1)h_n}\int_0^{(p+1)h_n}A^{(i,j)}(x)\min\{s,s'\}\left(V_{\rho,h_n}^{(i,i)}((p+1)h_n-s)\right)\\
&\qquad\left(V_{\rho,h_n}^{(j,j)}((p+1)h_n-s')-V_{\rho,h_n}^{(j,j)}(ph_n-s')\right)\mathrm{d}s'\mathrm{d}s\\
&=\frac{A^{(i,j)}(x)}{\rho^{(i)}\rho^{(j)}h_n^2}\left(\frac{(p+1)^2h_n^2}{2}-\frac{\left(p+1-\rho^{(i)}\right)^2h_n^2}{2}\right)((p+1)h_n)\\
&\quad-\frac{A^{(i,j)}(x)}{\rho^{(i)}\rho^{(j)}h_n^2}\left(\frac{(p+1)^3h_n^3}{3}-\frac{\left(p+1-\rho^{(i)}\right)^3h_n^3}{3}\right)\\
&\quad-\frac{A^{(i,j)}(x)}{\rho^{(i)}\rho^{(j)}h_n^2}\mathbf{1}_{(1,\bar{\rho}]}\left(\rho^{(i)}\right)\left(\frac{p^2h_n^2}{2}-\frac{\left(p+1-\rho^{(i)}\right)^2h_n^2}{2}\right)(ph_n)\\
&\quad+\frac{A^{(i,j)}(x)}{\rho^{(i)}\rho^{(j)}h_n^2}\mathbf{1}_{(1,\bar{\rho}]}\left(\rho^{(i)}\right)\left(\frac{p^3h_n^3}{3}-\frac{\left(p+1-\rho^{(i)}\right)^3h_n^3}{3}\right)
\end{aligned}
$$

$$
\begin{aligned}
&+\frac{A^{(i,j)}(x)}{\rho^{(i)}\rho^{(j)}h_n^2}\left(\frac{(p+1)^3h_n^3}{6}-\frac{\left(p+1-\rho^{(i)}\right)^3h_n^3}{6}\right)\\
&-\frac{A^{(i,j)}(x)}{\rho^{(i)}\rho^{(j)}h_n^2}\left(\rho^{(i)}h_n\right)\left(\frac{\left(p+1-\rho^{(j)}\right)^2h_n^2}{2}\right)\\
&-\frac{A^{(i,j)}(x)}{\rho^{(i)}\rho^{(j)}h_n^2}\mathbf{1}_{(1,\bar{\rho}]}\left(\rho^{(i)}\right)\left(\frac{p^2h_n^2}{2}-\frac{\left(p-\rho^{(j)}\right)^2h_n^2}{2}\right)h_n\\
&-\frac{A^{(i,j)}(x)}{\rho^{(i)}\rho^{(j)}h_n^2}\mathbf{1}_{(1,\bar{\rho}]}\left(\rho^{(i)}\right)\left(\frac{p^3h_n^3}{6}-\frac{\left(p+1-\rho^{(i)}\right)^3h_n^3}{6}\right)\\
&+\frac{A^{(i,j)}(x)}{\rho^{(i)}\rho^{(j)}h_n^2}\mathbf{1}_{(1,\bar{\rho}]}\left(\rho^{(i)}\right)\left(\left(\rho^{(i)}-1\right)h_n\right)\left(\frac{\left(p-\rho^{(j)}\right)^2h_n^2}{2}\right)\\
=&\frac{h_nA^{(i,j)}(x)}{\rho^{(i)}\rho^{(j)}}\left(-\frac{\left(\rho^{(i)}\right)^2}{2}+\rho^{(i)}\rho^{(j)}+\frac{\rho^{(i)}}{2}-\frac{1}{6}\right),
\end{aligned}
$$

and for the case (iv),

$$
\begin{aligned}
&h_n\mathbf{K}^{(i,j)}(x|\rho)\\
&=\int_0^{(p+1)h_n}\int_0^{(p+1)h_n}A^{(i,j)}(x)\min\{s,s'\}\left(V_{\rho,h_n}^{(i,i)}((p+1)h_n-s)\right)\\
&\qquad\left(V_{\rho,h_n}^{(j,j)}((p+1)h_n-s')-V_{\rho,h_n}^{(j,j)}(ph_n-s')\right)\mathrm{d}s'\mathrm{d}s\\
&=\frac{A^{(i,j)}(x)}{\rho^{(i)}\rho^{(j)}h_n^2}\left(\frac{(p+1)^2h_n^2}{2}-\frac{\left(p+1-\rho^{(j)}\right)^2h_n^2}{2}\right)((p+1)h_n)\\
&-\frac{A^{(i,j)}(x)}{\rho^{(i)}\rho^{(j)}h_n^2}\left(\frac{(p+1)^3h_n^3}{3}-\frac{\left(p+1-\rho^{(j)}\right)^3h_n^3}{3}\right)\\
&+\frac{A^{(i,j)}(x)}{\rho^{(i)}\rho^{(j)}h_n^2}\mathbf{1}_{\left(\rho^{(j)},\bar{\rho}\right]}\left(\rho^{(i)}\right)\left(\frac{\left(p+1-\rho^{(j)}\right)^2h_n^2}{2}-\frac{\left(p+1-\rho^{(i)}\right)^2h_n^2}{2}\right)\left(\rho^{(j)}h_n\right)\\
&+\frac{A^{(i,j)}(x)}{\rho^{(i)}\rho^{(j)}h_n^2}\left(\frac{(p+1)^3h_n^3}{6}-\frac{\left(p+1-\rho^{(j)}\right)^3h_n^3}{6}\right)\\
&-\frac{A^{(i,j)}(x)}{\rho^{(i)}\rho^{(j)}h_n^2}\left(\rho^{(j)}h_n\right)\left(\frac{\left(p+1-\rho^{(j)}\right)^2h_n^2}{2}\right)\\
&-\frac{A^{(i,j)}(x)}{\rho^{(i)}\rho^{(j)}h_n^2}\mathbf{1}_{(0,1]}\left(\rho^{(i)}\right)\left(\frac{p^2h_n^2}{2}-\frac{\left(p-\rho^{(j)}\right)^2h_n^2}{2}\right)\left(\rho^{(i)}h_n\right)\\
&=\frac{h_nA^{(i,j)}(x)}{\rho^{(i)}\rho^{(j)}}\left(-\frac{\left(\rho^{(i)}\right)^2\rho^{(j)}}{2}+\frac{\rho^{(i)}\left(\rho^{(j)}\right)^2}{2}+\rho^{(i)}\rho^{(j)}-\frac{\left(\rho^{(j)}\right)^3}{6}\right),
\end{aligned}
$$

and for the case (v),

$$h_n\mathbf{K}^{(i,j)}(x|\rho)$$

$$\begin{aligned}
&= \int_0^{(p+1)h_n} \int_0^{(p+1)h_n} A^{(i,j)}(x) \min\{s,s'\} \left(V_{\rho,h_n}^{(i,i)}((p+1)h_n - s)\right) \\
&\qquad \left(V_{\rho,h_n}^{(j,j)}((p+1)h_n - s') - V_{\rho,h_n}^{(j,j)}(ph_n - s')\right) \mathrm{d}s'\mathrm{d}s \\
&= \frac{A^{(i,j)}(x)}{\rho^{(i)}\rho^{(j)}h_n^2} \left(\frac{(p+1)^2 h_n^2}{2} - \frac{\left(p+1-\rho^{(i)}\right)^2 h_n^2}{2}\right)((p+1)h_n) \\
&\quad - \frac{A^{(i,j)}(x)}{\rho^{(i)}\rho^{(j)}h_n^2} \left(\frac{(p+1)^3 h_n^3}{3} - \frac{\left(p+1-\rho^{(i)}\right)^3 h_n^3}{3}\right) \\
&\quad + \frac{A^{(i,j)}(x)}{\rho^{(i)}\rho^{(j)}h_n^2} \left(\frac{(p+1)^3 h_n^3}{6} - \frac{\left(p+1-\rho^{(i)}\right)^3 h_n^3}{6}\right) \\
&\quad - \frac{A^{(i,j)}(x)}{\rho^{(i)}\rho^{(j)}h_n^2} \left(\rho^{(i)}h_n\right) \left(\frac{\left(p+1-\rho^{(j)}\right)^2 h_n^2}{2}\right) \\
&\quad - \frac{A^{(i,j)}(x)}{\rho^{(i)}\rho^{(j)}h_n^2} \mathbf{1}_{(0,1]}\left(\rho^{(i)}\right) \left(\frac{p^2h_n^2}{2} - \frac{\left(p-\rho^{(j)}\right)^2 h_n^2}{2}\right)\left(\rho^{(i)}h_n\right) \\
&= \frac{h_n A^{(i,j)}(x)}{\rho^{(i)}\rho^{(j)}} \left(\rho^{(i)}\rho^{(j)} - \frac{\left(\rho^{(i)}\right)^3}{6}\right);
\end{aligned}$$

therefore, we obtain

$$\begin{aligned}
&\mathbf{G}^{(i,j)}(x|\rho) + \mathbf{D}_0^{(i,j)}(x|\rho) \\
&= \begin{cases}
A^{(i,j)}(x) & \text{if } \rho^{(i)} = 0, \\
A^{(i,j)}(x)\left(1 - \frac{\rho^{(i)}}{2}\right) & \text{if } \rho^{(i)} \in (0,1], \rho^{(j)} = 0, \\
A^{(i,j)}(x)\frac{1}{2\rho^{(i)}} & \text{if } \rho^{(i)} \in (1,\overline{\rho}], \rho^{(j)} = 0, \\
A^{(i,j)}(x)\frac{\left(\rho^{(j)}\right)^2 + \rho^{(j)}}{2\rho^{(i)}\rho^{(j)}} & \text{if } \rho^{(i)} \in \left(\rho^{(j)}+1,\overline{\rho}\right], \rho^{(j)} > 0, \\
A^{(i,j)}(x)\frac{\left(\rho^{(i)}-\rho^{(j)}\right)^3 - 3\left(\rho^{(i)}\right)^2 + 6\rho^{(i)}\rho^{(j)} + 3\rho^{(i)} - 1}{6\rho^{(i)}\rho^{(j)}} & \text{if } \rho^{(i)} \in (1,\overline{\rho}], \rho^{(i)} \in \left(\rho^{(j)},\rho^{(j)}+1\right], \\
A^{(i,j)}(x)\frac{-3\left(\rho^{(i)}\right)^2 + 6\rho^{(i)}\rho^{(j)} + 3\rho^{(i)} - 1}{6\rho^{(i)}\rho^{(j)}} & \text{if } \rho^{(i)} \in (1,\overline{\rho}], \rho^{(i)} \le \rho^{(j)}, \\
A^{(i,j)}(x)\frac{-3\left(\rho^{(i)}\right)^2\rho^{(j)} + 3\rho^{(i)}\left(\rho^{(j)}\right)^2 + 6\rho^{(i)}\rho^{(j)} - \left(\rho^{(j)}\right)^3}{6\rho^{(i)}\rho^{(j)}} & \text{if } \rho^{(i)} \in (0,1], \rho^{(i)} > \rho^{(j)}, \\
A^{(i,j)}(x)\frac{6\rho^{(i)}\rho^{(j)} - \left(\rho^{(i)}\right)^3}{6\rho^{(i)}\rho^{(j)}} & \text{if } \rho^{(i)} \in (0,1], \rho^{(i)} \le \rho^{(j)},
\end{cases}
\end{aligned}$$

and $\mathbf{G}(x|\rho) = \mathbb{G}(x|\rho) = \mathbb{G}(x,\alpha|\rho)|_{\alpha=\alpha_\star}$.

Proof of the results in Section 3.1.

Proof of Lemma 1. By following the proof of the Proposition 9, it is sufficient to evaluate

$$\begin{aligned}
&\int_0^{(p+2)h_n} \int_0^{(p+2)h_n} A^{(i,i)}(x) \min\{s,s'\} \left(V_{\rho,h_n}^{(i,i)}((p+2)h_n - s) - V_{\rho,h_n}^{(i,i)}(ph_n - s)\right) \\
&\qquad \left(V_{\rho,h_n}^{(i,i)}((p+2)h_n - s') - V_{\rho,h_n}^{(i,i)}(ph_n - s')\right) \mathrm{d}s'\mathrm{d}s
\end{aligned}$$

for the asymptotic behaviour of the reduced quadratic variation. If $\rho^{(i)}=0$,

$$\begin{aligned}
&\int_0^{(p+2)h_n}\int_0^{(p+2)h_n} A^{(i,i)}(x)\min\{s,s'\}\left(V_{\rho,h_n}^{(i,i)}((p+2)h_n-s)-V_{\rho,h_n}^{(i,i)}(ph_n-s)\right)\\
&\qquad\left(V_{\rho,h_n}^{(i,i)}((p+2)h_n-s')-V_{\rho,h_n}^{(i,i)}(ph_n-s')\right)\mathrm{d}s'\mathrm{d}s\\
&=A^{(i,i)}(x)\int_0^{(p+2)h_n}\int_0^{(p+2)h_n}\min\{s,s'\}\left(\delta((p+2)h_n-s)-\delta(ph_n-s)\right)\\
&\qquad\left(\delta((p+2)h_n-s')-\delta(ph_n-s')\right)\mathrm{d}s'\mathrm{d}s\\
&=A^{(i,i)}(x)\left((p+2)h_n-2ph_n+ph_n\right)\\
&=2h_nA^{(i,i)}(x),
\end{aligned}$$

and if $\rho^{(i)}\in(0,\overline{\rho}]$,

$$\begin{aligned}
&\int_0^{(p+2)h_n}\int_0^{(p+2)h_n} A^{(i,i)}(x)\min\{s,s'\}\left(V_{\rho,h_n}^{(i,i)}((p+2)h_n-s)-V_{\rho,h_n}^{(i,i)}(ph_n-s)\right)\\
&\qquad\left(V_{\rho,h_n}^{(i,i)}((p+2)h_n-s')-V_{\rho,h_n}^{(i,i)}(ph_n-s')\right)\mathrm{d}s'\mathrm{d}s\\
&=\frac{A^{(i,i)}(x)}{\left(\rho^{(i)}h_n\right)^2}\int_0^{(p+2)h_n}\int_0^{(p+2)h_n}\min\{s,s'\}\\
&\qquad\times\left(\mathbf{1}_{\left[0,\rho^{(i)}h_n\right]}((p+2)h_n-s)-\mathbf{1}_{\left[0,\rho^{(i)}h_n\right]}(ph_n-s)\right)\\
&\qquad\times\left(\mathbf{1}_{\left[0,\rho^{(i)}h_n\right]}((p+2)h_n-s')-\mathbf{1}_{\left[0,\rho^{(i)}h_n\right]}(ph_n-s')\right)\mathrm{d}s'\mathrm{d}s\\
&=\frac{A^{(i,i)}(x)}{\left(\rho^{(i)}h_n\right)^2}\int_{\left(p+2-\rho^{(i)}\right)h_n}^{(p+2)h_n}\int_{\left(p+2-\rho^{(i)}\right)h_n}^{(p+2)h_n}\min\{s,s'\}\mathrm{d}s'\mathrm{d}s\\
&\quad-\frac{2A^{(i,i)}(x)}{\left(\rho^{(i)}h_n\right)^2}\int_{\left(p+2-\rho^{(i)}\right)h_n}^{(p+2)h_n}\int_{\left(p-\rho^{(i)}\right)h_n}^{ph_n}\min\{s,s'\}\mathrm{d}s'\mathrm{d}s\\
&\quad+\frac{A^{(i,i)}(x)}{\left(\rho^{(i)}h_n\right)^2}\int_{\left(p-\rho^{(i)}\right)h_n}^{ph_n}\int_{\left(p-\rho^{(i)}\right)h_n}^{ph_n}\min\{s,s'\}\mathrm{d}s'\mathrm{d}s\\
&=\frac{A^{(i,i)}(x)}{\left(\rho^{(i)}h_n\right)^2}\int_{\left(p+2-\rho^{(i)}\right)h_n}^{(p+2)h_n}\left(\int_s^{(p+2)h_n}s\mathrm{d}s'+\int_{\left(p+2-\rho^{(i)}\right)h_n}^{s}s'\mathrm{d}s'\right)\mathrm{d}s\\
&\quad-\frac{2A^{(i,i)}(x)}{\left(\rho^{(i)}h_n\right)^2}\mathbf{1}_{(2,\overline{\rho}]}\left(\rho^{(i)}\right)\int_{ph_n}^{(p+2)h_n}\int_{\left(p-\rho^{(i)}\right)h_n}^{ph_n}s'\mathrm{d}s'\mathrm{d}s\\
&\quad-\frac{2A^{(i,i)}(x)}{\left(\rho^{(i)}h_n\right)^2}\mathbf{1}_{(2,\overline{\rho}]}\left(\rho^{(i)}\right)\int_{\left(p+2-\rho^{(i)}\right)h_n}^{ph_n}\left(\int_s^{ph_n}s\mathrm{d}s'+\int_{\left(p-\rho^{(i)}\right)h_n}^{s}s'\mathrm{d}s'\right)\mathrm{d}s\\
&\quad-\frac{2A^{(i,i)}(x)}{\left(\rho^{(i)}h_n\right)^2}\mathbf{1}_{(0,2]}\left(\rho^{(i)}\right)\int_{\left(p+2-\rho^{(i)}\right)h_n}^{(p+2)h_n}\int_{\left(p-\rho^{(i)}\right)h_n}^{ph_n}s'\mathrm{d}s'\mathrm{d}s\\
&\quad+\frac{A^{(i,i)}(x)}{\left(\rho^{(i)}h_n\right)^2}\int_{\left(p-\rho^{(i)}\right)h_n}^{ph_n}\left(\int_s^{ph_n}s\mathrm{d}s'+\int_{\left(p-\rho^{(i)}\right)h_n}^{s}s'\mathrm{d}s'\right)\mathrm{d}s
\end{aligned}$$

$$
\begin{aligned}
&= \frac{A^{(i,i)}(x)}{(\rho^{(i)}h_n)^2}\int_{(p+2-\rho^{(i)})h_n}^{(p+2)h_n}\left(\left((p+2)h_n s - s^2\right) + \left(\frac{s^2}{2} - \frac{\left(p+2-\rho^{(i)}\right)^2 h_n^2}{2}\right)\right)\mathrm{d}s\\
&\quad - \frac{A^{(i,i)}(x)}{(\rho^{(i)}h_n)^2}\mathbf{1}_{(2,\bar{\rho}]}\left(\rho^{(i)}\right)\int_{ph_n}^{(p+2)h_n}\left(p^2h_n^2 - \left(p-\rho^{(i)}\right)^2 h_n^2\right)\mathrm{d}s\\
&\quad - \frac{2A^{(i,i)}(x)}{(\rho^{(i)}h_n)^2}\mathbf{1}_{(2,\bar{\rho}]}\left(\rho^{(i)}\right)\int_{(p+2-\rho^{(i)})h_n}^{ph_n}\left(\left(ph_n s - s^2\right) + \left(\frac{s^2}{2} - \frac{\left(p-\rho^{(i)}\right)^2 h_n^2}{2}\right)\right)\mathrm{d}s\\
&\quad - \frac{2A^{(i,i)}(x)}{(\rho^{(i)}h_n)^2}\mathbf{1}_{(0,2]}\left(\rho^{(i)}\right)\int_{(p+2-\rho^{(i)})h_n}^{(p+2)h_n}\left(\frac{p^2h_n^2}{2} - \frac{\left(p-\rho^{(i)}\right)^2 h_n^2}{2}\right)\mathrm{d}s\\
&\quad + \frac{A^{(i,i)}(x)}{(\rho^{(i)}h_n)^2}\int_{(p-\rho^{(i)})h_n}^{ph_n}\left(\left(ph_n s - s^2\right) + \left(\frac{s^2}{2} - \frac{\left(p-\rho^{(i)}\right)^2 h_n^2}{2}\right)\right)\mathrm{d}s\\
&= \frac{A^{(i,i)}(x)}{(\rho^{(i)}h_n)^2}\int_{(p+2-\rho^{(i)})h_n}^{(p+2)h_n}\left(-\frac{s^2}{2} + (p+2)h_n s - \frac{\left(p+2-\rho^{(i)}\right)^2 h_n^2}{2}\right)\mathrm{d}s\\
&\quad - \frac{A^{(i,i)}(x)}{(\rho^{(i)}h_n)^2}\mathbf{1}_{(2,\bar{\rho}]}\left(\rho^{(i)}\right)\left(p^2h_n^2 - \left(p-\rho^{(i)}\right)^2 h_n^2\right)2h_n\\
&\quad - \frac{2A^{(i,i)}(x)}{(\rho^{(i)}h_n)^2}\mathbf{1}_{(2,\bar{\rho}]}\left(\rho^{(i)}\right)\int_{(p+2-\rho^{(i)})h_n}^{ph_n}\left(-\frac{s^2}{2} + ph_n s - \frac{\left(p-\rho^{(i)}\right)^2 h_n^2}{2}\right)\mathrm{d}s\\
&\quad - \frac{2A^{(i,i)}(x)}{(\rho^{(i)}h_n)^2}\mathbf{1}_{(0,2]}\left(\rho^{(i)}\right)\left(\frac{p^2h_n^2}{2} - \frac{\left(p-\rho^{(i)}\right)^2 h_n^2}{2}\right)\rho^{(i)}h_n\\
&\quad + \frac{A^{(i,i)}(x)}{(\rho^{(i)}h_n)^2}\int_{(p-\rho^{(i)})h_n}^{ph_n}\left(-\frac{s^2}{2} + ph_n s - \frac{\left(p-\rho^{(i)}\right)^2 h_n^2}{2}\right)\mathrm{d}s\\
&= -\frac{A^{(i,i)}(x)}{(\rho^{(i)}h_n)^2}\left(\frac{(p+2)^3 h_n^3}{6} - \frac{\left(p+2-\rho^{(i)}\right)^3 h_n^3}{6}\right)\\
&\quad + \frac{A^{(i,i)}(x)}{(\rho^{(i)}h_n)^2}\left(\frac{(p+2)^2 h_n^2}{2} - \frac{\left(p+2-\rho^{(i)}\right)^2 h_n^2}{2}\right)(p+2)h_n\\
&\quad - \frac{A^{(i,i)}(x)}{(\rho^{(i)}h_n)^2}\frac{\left(p+2-\rho^{(i)}\right)^2\rho^{(i)}h_n^3}{2}\\
&\quad - \frac{A^{(i,i)}(x)}{(\rho^{(i)}h_n)^2}\mathbf{1}_{(2,\bar{\rho}]}\left(\rho^{(i)}\right)\left(p^2h_n^2 - \left(p-\rho^{(i)}\right)^2 h_n^2\right)2h_n\\
&\quad + \frac{A^{(i,i)}(x)}{(\rho^{(i)}h_n)^2}\mathbf{1}_{(2,\bar{\rho}]}\left(\rho^{(i)}\right)\left(\frac{p^3h_n^3}{3} - \frac{\left(p+2-\rho^{(i)}\right)^3 h_n^3}{3}\right)\\
&\quad - \frac{A^{(i,i)}(x)}{(\rho^{(i)}h_n)^2}\mathbf{1}_{(2,\bar{\rho}]}\left(\rho^{(i)}\right)\left(p^2h_n^2 - \left(p+2-\rho^{(i)}\right)^2 h_n^2\right)ph_n\\
&\quad + \frac{A^{(i,i)}(x)}{(\rho^{(i)}h_n)^2}\mathbf{1}_{(2,\bar{\rho}]}\left(\rho^{(i)}\right)\left(p-\rho^{(i)}\right)^2\left(\rho^{(i)}-2\right)h_n^3
\end{aligned}
$$

$$
\begin{aligned}
&- \frac{A^{(i,i)}(x)}{\left(\rho^{(i)} h_n\right)^2} \mathbf{1}_{(0,2]}\left(\rho^{(i)}\right)\left(p^2 \rho^{(i)} h_n^3 - \left(p-\rho^{(i)}\right)^2 \rho^{(i)} h_n^3\right) \\
&- \frac{A^{(i,i)}(x)}{\left(\rho^{(i)} h_n\right)^2}\left(\frac{p^3 h_n^3}{6} - \frac{\left(p-\rho^{(i)}\right)^3 h_n^3}{6}\right) \\
&+ \frac{A^{(i,i)}(x)}{\left(\rho^{(i)} h_n\right)^2}\left(\frac{p^2 h_n^2}{2} - \frac{\left(p-\rho^{(i)}\right)^2 h_n^2}{2}\right) p h_n \\
&- \frac{A^{(i,i)}(x)}{\left(\rho^{(i)} h_n\right)^2} \frac{\left(p-\rho^{(i)}\right)^2 \rho^{(i)} h_n^3}{2} \\
&= \begin{cases} 2h_n A^{(i,i)}(x)\left(1 - \frac{\rho^{(i)}}{6}\right) & \text{if } \rho^{(i)} \in (0,2], \\ 2h_n A^{(i,i)}(x)\left(\frac{2}{\rho^{(i)}} - \frac{4}{3\left(\rho^{(i)}\right)^2}\right) & \text{if } \rho^{(i)} \in (2,\overline{\rho}]. \end{cases}
\end{aligned}
$$

Hence, we obtain the proof. □

Proof of Lemma 2. Continuity is obvious, and monotonicity is obtained as follows: if $\rho^{(i)} \in (0,1]$,

$$
\begin{aligned}
\frac{\mathrm{d}}{\mathrm{d}\rho^{(i)}}\left(6-2\rho^{(i)}\right)\left(6-\rho^{(i)}\right)^{-1} &= \left((-2)\left(6-\rho^{(i)}\right) - \left(6-2\rho^{(i)}\right)(-1)\right)\left(6-\rho^{(i)}\right)^{-2} \\
&= (-12)\left(6-\rho^{(i)}\right)^{-2} < 0,
\end{aligned}
$$

and if $\rho^{(i)} \in (1,2]$,

$$
\begin{aligned}
&\frac{\mathrm{d}}{\mathrm{d}\rho^{(i)}}\left(6\rho^{(i)}-2\right)\left(6\left(\rho^{(i)}\right)^2 - \left(\rho^{(i)}\right)^3\right)^{-1} \\
&= \left(6\left(6\left(\rho^{(i)}\right)^2 - \left(\rho^{(i)}\right)^3\right) - \left(6\rho^{(i)}-2\right)\left(12\rho^{(i)} - 3\left(\rho^{(i)}\right)^2\right)\right)\left(6\left(\rho^{(i)}\right)^2 - \left(\rho^{(i)}\right)^3\right)^{-2} \\
&= 6\rho^{(i)}\left(-7\rho^{(i)} + 4 + 2\left(\rho^{(i)}\right)^2\right)\left(6\left(\rho^{(i)}\right)^2 - \left(\rho^{(i)}\right)^3\right)^{-2} < 0,
\end{aligned}
$$

and if $\rho^{(i)} \in (2,\overline{\rho}]$,

$$
\frac{\mathrm{d}}{\mathrm{d}\rho^{(i)}}\left(3\rho^{(i)}-1\right)\left(6\rho^{(i)}-4\right)^{-1} = (-18)\left(6\rho^{(i)}-4\right)^{-2} < 0.
$$

The inverse can be obtained directly. □

Proof of Theorem 3. It follows from Lemma 1, 2 and continuous mapping theorem. □

Proofs of the results in Section 3.2.

Proof of Theorem 4. We can clearly prove the result by using Lemma 7 in Kessler (1997), Proposition 7 in Nakakita and Uchida (2019), and Slutsky's theorem. □

Proof of Theorem 5. By Lemma 1, there exists a number $\ell < 0$ such that

$$
\frac{1}{nh_n}\sum_{k=1}^{n}\left(\overline{X}^{(i)}_{kh_n,n} - \overline{X}^{(i)}_{(k-1)h_n,n}\right)^2 - \frac{1}{nh_n}\sum_{2\le 2k\le n}\left(\overline{X}^{(i)}_{2kh_n,n} - \overline{X}^{(i)}_{(2k-2)h_n,n}\right)^2 \to^P \ell < 0,
$$

and hence it is sufficient to show that

$$\sup_{n\in\mathbf{N}} \mathbf{E}\left[\frac{2}{3nh_n^2}\sum_{k=1}^{n}\left(\overline{X}^{(i)}_{kh_n,n}-\overline{X}^{(i)}_{(k-1)h_n,n}\right)^4\right]<\infty;$$

and it is obvious that

$$\left(\overline{X}^{(i)}_{kh_n,n}-\overline{X}^{(i)}_{(k-1)h_n,n}\right)^4 \le C\left(\overline{X}^{(i)}_{kh_n,n}-X_{(k-\overline{\rho}-1)h_n}\right)^4 + C\left(\overline{X}^{(i)}_{(k-1)h_n,n}-X_{(k-\overline{\rho}-1)h_n}\right)^4$$

and

$$\begin{aligned}
&\mathbf{E}\left[\left(\overline{X}^{(i)}_{kh_n,n}-X_{(k-\overline{\rho}-1)h_n}\right)^4\middle|\mathcal{F}_{(k-\overline{\rho}-1)h_n}\right]\\
&=\mathbf{E}\left[\left(\frac{1}{\rho_\star^{(i)}h_n}\int_{\left(k-\rho_\star^{(i)}\right)h_n}^{kh_n}\left(X_s^{(i)}-X_{(k-\overline{\rho}-1)h_n}\right)\mathrm{d}s\right)^4\middle|\mathcal{F}_{(k-\overline{\rho}-1)h_n}\right]\\
&\le\mathbf{E}\left[\left(\frac{1}{\rho_\star^{(i)}h_n}\int_{\left(k-\rho_\star^{(i)}\right)h_n}^{kh_n}\left|X_s^{(i)}-X_{(k-\overline{\rho}-1)h_n}\right|\mathrm{d}s\right)^4\middle|\mathcal{F}_{(k-\overline{\rho}-1)h_n}\right]\\
&\le\mathbf{E}\left[\left(\frac{1}{\rho_\star^{(i)}h_n}\int_{\left(k-\rho_\star^{(i)}\right)h_n}^{kh_n}\sup_{s'\in\left[\left(k-\rho_\star^{(i)}\right)h_n,kh_n\right]}\left|X_{s'}^{(i)}-X_{(k-\overline{\rho}-1)h_n}\right|\mathrm{d}s\right)^4\middle|\mathcal{F}_{(k-\overline{\rho}-1)h_n}\right]\\
&=\mathbf{E}\left[\sup_{s\in\left[\left(k-\rho_\star^{(i)}\right)h_n,kh_n\right]}\left|X_s^{(i)}-X_{(k-\overline{\rho}-1)h_n}\right|^4\middle|\mathcal{F}_{(k-\overline{\rho}-1)h_n}\right]\\
&\le Ch_n\left(1+\left|X_{(k-\overline{\rho}-1)h_n}\right|\right)^C
\end{aligned}$$

by Proposition A in Gloter (2000), and a parallel result holds for $\left(\overline{X}^{(i)}_{(k-1)h_n,n}-X_{(k-\overline{\rho}-1)h_n}\right)^4$. Hence we obtain the result. □

Proof of the results in Section 4.

Proof of Theorem 6. We only deal with the case where $\rho_\star$ is unknown because the discussion for the case where $\rho_\star$ is known is parallel. First of all, we prove the consistency of $\hat{\alpha}_n$. We obtain that

$$\begin{aligned}
&\left|\frac{1}{n}\mathbb{H}_{1,n}\left(\alpha|\hat{\rho}_n\right)-\frac{1}{n}\mathbb{H}_{1,n}\left(\alpha|\rho_\star\right)\right|\\
&=\left|-\frac{1}{n}\sum_{k=1}^{n}\left\|\frac{1}{h_n}\left(\overline{X}_{kh_n,n}-\overline{X}_{(k-1)h_n,n}\right)^{\otimes 2}-\mathbb{G}\left(\overline{X}_{(k-1)h_n,n},\alpha|\hat{\rho}_n\right)\right\|^2\right.\\
&\qquad\left.+\frac{1}{n}\sum_{k=1}^{n}\left\|\frac{1}{h_n}\left(\overline{X}_{kh_n,n}-\overline{X}_{(k-1)h_n,n}\right)^{\otimes 2}-\mathbb{G}\left(\overline{X}_{(k-1)h_n,n},\alpha|\rho_\star\right)\right\|^2\right|\\
&\le\left|\frac{2}{nh_n}\sum_{k=1}^{n}\left(\mathbb{G}\left(\overline{X}_{(k-1)h_n,n},\alpha|\hat{\rho}_n\right)-\mathbb{G}\left(\overline{X}_{(k-1)h_n,n},\alpha|\rho_\star\right)\right)\left[\left(\overline{X}_{kh_n,n}-\overline{X}_{(k-1)h_n,n}\right)^{\otimes 2}\right]\right|\\
&\quad+\left|\frac{1}{n}\sum_{k=1}^{n}\left(\left\|\mathbb{G}\left(\overline{X}_{(k-1)h_n,n},\alpha|\hat{\rho}_n\right)\right\|^2-\left\|\mathbb{G}\left(\overline{X}_{(k-1)h_n,n},\alpha|\rho_\star\right)\right\|^2\right)\right|
\end{aligned}$$

$$
\begin{aligned}
&\leq \frac{2}{nh_n}\sum_{k=1}^{n}\left|\overline{X}_{kh_n,n}-\overline{X}_{(k-1)h_n,n}\right|^2 \\
&\qquad\times\sum_{i=1}^{d}\sum_{j=1}^{d}\left|A^{(i,j)}\left(\overline{X}_{(k-1)h_n,n},\alpha\right)\right|\left|f_{\mathbb{G}}\left(\hat{\rho}_n^{(i)},\hat{\rho}_n^{(j)}\right)-f_{\mathbb{G}}\left(\rho_\star^{(i)},\rho_\star^{(j)}\right)\right| \\
&\quad+\frac{1}{n}\sum_{k=1}^{n}\sum_{i=1}^{d}\sum_{j=1}^{d}\left|A^{(i,j)}\left(\overline{X}_{(k-1)h_n,n},\alpha\right)\right|^2\left|f_{\mathbb{G}}^2\left(\hat{\rho}_n^{(i)},\hat{\rho}_n^{(j)}\right)-f_{\mathbb{G}}^2\left(\rho_\star^{(i)},\rho_\star^{(j)}\right)\right| \\
&\leq \frac{C}{nh_n}\sum_{k=1}^{n}\left(1+\left|\overline{X}_{(k-1)h_n,n}\right|\right)^C\left|\overline{X}_{kh_n,n}-\overline{X}_{(k-1)h_n,n}\right|^2 \\
&\qquad\times\sum_{i=1}^{d}\sum_{j=1}^{d}\left|f_{\mathbb{G}}\left(\hat{\rho}_n^{(i)},\hat{\rho}_n^{(j)}\right)-f_{\mathbb{G}}\left(\rho_\star^{(i)},\rho_\star^{(j)}\right)\right| \\
&\quad+\frac{C}{n}\sum_{k=1}^{n}\left(1+\left|\overline{X}_{(k-1)h_n,n}\right|\right)^C\sum_{i=1}^{d}\sum_{j=1}^{d}\left|f_{\mathbb{G}}^2\left(\hat{\rho}_n^{(i)},\hat{\rho}_n^{(j)}\right)-f_{\mathbb{G}}^2\left(\rho_\star^{(i)},\rho_\star^{(j)}\right)\right| \\
&\to^P 0 \text{ uniformly in } \alpha,
\end{aligned}
$$

because continuous mapping theorem holds. Therefore, it follows from Proposition 7 and Proposition 9 that

$$
\begin{aligned}
&\frac{1}{n}\mathbb{H}_{1,n}\left(\alpha|\hat{\rho}_n\right)-\frac{1}{n}\mathbb{H}_{1,n}\left(\alpha_\star|\rho_\star\right) \\
&=\frac{2}{nh_n}\sum_{k=1}^{n}\mathbb{G}\left(\overline{X}_{(k-1)h_n,n},\alpha|\rho_\star\right)\left[\left(\overline{X}_{kh_n,n}-\overline{X}_{(k-1)h_n,n}\right)^{\otimes 2}\right] \\
&\quad-\frac{1}{n}\sum_{k=1}^{n}\left\|\mathbb{G}\left(\overline{X}_{(k-1)h_n,n},\alpha|\rho_\star\right)\right\|^2 \\
&\quad-\frac{2}{nh_n}\sum_{k=1}^{n}\mathbb{G}\left(\overline{X}_{(k-1)h_n,n},\alpha_\star|\rho_\star\right)\left[\left(\overline{X}_{kh_n,n}-\overline{X}_{(k-1)h_n,n}\right)^{\otimes 2}\right] \\
&\quad+\frac{1}{n}\sum_{k=1}^{n}\left\|\mathbb{G}\left(\overline{X}_{(k-1)h_n,n},\alpha_\star|\rho_\star\right)\right\|^2 \\
&\quad+o_P^*(1) \\
&\to^P \mathbb{V}_1\left(\alpha|\xi_\star\right) \text{ uniformly in } \alpha
\end{aligned}
$$

where $o_P^*(1)$ indicates the term converging in probability to zero uniformly in θ. Then we obtain that $\hat{\alpha}_n \to \alpha_\star$ in the same way as Kessler (1997) with Assumption [A3].

In the next place, we consider the consistency of $\hat{\beta}_n$. Firstly, we consider the case $\max_i \rho_\star^{(i)} \in (\ell-1,\ell)$ for an integer $\ell \in \{1,\ldots,[\overline{\rho}]+1\}$. Then it is sufficient to show

$$
\frac{1}{nh_n}\mathbb{H}_{2,n}\left(\beta|\hat{\rho}_n\right)-\frac{1}{nh_n}\mathbb{H}_{2,n}\left(\beta_\star|\rho_\star\right)\to^P \mathbb{V}_2\left(\beta|\xi_\star\right) \text{ uniformly in } \beta
$$

due to Assumption [A3]. Because the evaluation $D_j(x)=O$ where $j \geq \left[\max_{i=1,\ldots,n}\rho_*^{(i)}\right]+1$ using independent increments of the Wiener process, Proposition 7 and Proposition 8

verify

$$F_\ell(\beta) - \frac{1}{nh_n}\mathbb{H}_{2,n}(\beta_\star|\rho_\star) \to^P \mathbb{V}_2(\beta|\xi_\star) \text{ uniformly in } \beta,$$

where

$$F_j(\beta) := -\frac{1}{nh_n^2}\sum_{k=1+j}^{n}\left|\overline{X}_{kh_n,n} - \overline{X}_{(k-1)h_n,n} - h_n b\left(\overline{X}_{(k-1-j)h_n,n}, \beta\right)\right|^2.$$

In addition, the exact convergences such that

$$P\left(\mathbf{1}_{\{\ell\}}\left(\left[\max_i \hat{\rho}_n^{(i)}\right]+1\right)=1\right) \to 1,\ P\left(\mathbf{1}_{\{j\}}\left(\left[\max_i \hat{\rho}_n^{(i)}\right]+1\right)=1\right) \to 0$$

hold for all $j \neq \ell$, since for all $j = 1, \ldots, [\overline{\rho}] + 1$,

$$P\left(\mathbf{1}_{\{j\}}\left(\left[\max_i \hat{\rho}_n^{(i)}\right]+1\right)=1\right) = P\left(\max_i \hat{\rho}_n^{(i)} \in [j-1, j)\right).$$

Therefore, for any $\epsilon > 0$,

$$\begin{aligned}
&P\left(\sup_{\beta\in\Theta_2}\left|\frac{1}{nh_n}\mathbb{H}_{2,n}(\beta|\hat{\rho}_n) - \frac{1}{nh_n}\mathbb{H}_{2,n}(\beta_\star|\rho_\star) - \mathbb{V}_2(\beta|\xi_\star)\right| > \epsilon\right)\\
&\le \sum_{j\neq\ell} P\left(\mathbf{1}_{\{j\}}\left(\left[\max_i \hat{\rho}_n^{(i)}\right]+1\right)=1\right)\\
&\quad + P\left(\left\{\mathbf{1}_{\{\ell\}}\left(\left[\max_i \hat{\rho}_n^{(i)}\right]+1\right)=1\right\}\right.\\
&\qquad\qquad \left.\cap\left\{\sup_{\beta\in\Theta_2}\left|F_\ell(\beta) - \frac{1}{nh_n}\mathbb{H}_{2,n}(\beta_\star|\rho_\star) - \mathbb{V}_2(\beta|\xi_\star)\right| > \epsilon\right\}\right)\\
&\to 0.
\end{aligned}$$

For the case $\max_i \rho_\star^{(i)} = \ell$ for an integer $\ell = \{0, \ldots, [\overline{\rho}] + 1\}$, we similarly obtain

$$\frac{1}{nh_n}\mathbb{H}_{2,n}(\beta|\hat{\rho}_n) - \frac{1}{nh_n}\mathbb{H}_{2,n}(\beta_\star|\rho_\star) \to^P \mathbb{V}_2(\beta|\xi_\star) \text{ uniformly in } \beta$$

because we have

$$\begin{aligned}
F_\ell(\beta) - \frac{1}{nh_n}\mathbb{H}_{2,n}(\beta_\star|\rho_\star) &\to^P \mathbb{V}_2(\beta|\xi_\star) \text{ uniformly in } \beta,\\
F_{\ell+1}(\beta) - \frac{1}{nh_n}\mathbb{H}_{2,n}(\beta_\star|\rho_\star) &\to^P \mathbb{V}_2(\beta|\xi_\star) \text{ uniformly in } \beta,
\end{aligned}$$

and

$$\begin{aligned}
&P\left(\mathbf{1}_{\{\ell\}}\left(\left[\max_i \hat{\rho}_n^{(i)}\right]+1\right) + \mathbf{1}_{\{\ell+1\}}\left(\left[\max_i \hat{\rho}_n^{(i)}\right]+1\right)=1\right) \to 1,\\
&P\left(\mathbf{1}_{\{j\}}\left(\left[\max_i \hat{\rho}_n^{(i)}\right]+1\right)=0\right) \to 1, \text{ for all } j \neq \ell, \ell+1,
\end{aligned}$$

and it holds that for any $\epsilon > 0$,

$$\begin{aligned}
&P\left(\sup_{\beta\in\Theta_2}\left|\frac{1}{nh_n}\mathbb{H}_{2,n}\left(\beta|\hat{\rho}_n\right)-\frac{1}{nh_n}\mathbb{H}_{2,n}\left(\beta_\star|\rho_\star\right)-\mathbb{V}_2\left(\beta|\xi_\star\right)\right|>\epsilon\right)\\
&\le\sum_{j\neq\ell,\ell+1}P\left(\mathbf{1}_{\{j\}}\left(\left[\max_i\hat{\rho}_n^{(i)}\right]+1\right)=1\right)\\
&\quad+P\left(\left\{\mathbf{1}_{\{\ell\}}\left(\left[\max_i\hat{\rho}_n^{(i)}\right]+1\right)=1\right\}\right.\\
&\qquad\qquad\left.\cap\left\{\sup_{\beta\in\Theta_2}\left|F_\ell\left(\beta\right)-\frac{1}{nh_n}\mathbb{H}_{2,n}\left(\beta_\star|\rho_\star\right)-\mathbb{V}_2\left(\beta|\xi_\star\right)\right|>\epsilon\right\}\right)\\
&\quad+P\left(\left\{\mathbf{1}_{\{\ell+1\}}\left(\left[\max_i\hat{\rho}_n^{(i)}\right]+1\right)=1\right\}\right.\\
&\qquad\qquad\left.\cap\left\{\sup_{\beta\in\Theta_2}\left|F_{\ell+1}\left(\beta\right)-\frac{1}{nh_n}\mathbb{H}_{2,n}\left(\beta_\star|\rho_\star\right)-\mathbb{V}_2\left(\beta|\xi_\star\right)\right|>\epsilon\right\}\right)\\
&\to 0.
\end{aligned}$$

Hence it is shown that $\hat{\beta}_n \to^P \beta_\star$ with Assumption [A3]. □

Appendix B. Additional discussion

The next lemma supports an exchangeability of integrals.

Lemma 14. *Let us fix $C > 0$. For any t_1, t_2 such that $0 \le t_1 \le t_2$ and $t_2 - t_1 \le C$, and $f : \mathbf{R}_+ \to \mathbf{R}^d \otimes \mathbf{R}^r$ such that $f \in L^1([t_1, t_2])$, the following equation holds:*

$$\int_{t_1}^{t_2} f\left(s\right)\left(\int_{t_1}^{s_1}\mathrm{d}w_{s_2}\right)\mathrm{d}s_1=\int_{t_1}^{t_2}\left(\int_{s_1}^{t_2}f\left(s_2\right)\mathrm{d}s_2\right)\mathrm{d}w_{s_1};$$

and both are normally distributed with mean $\mathbf{0}$ and variance $\int_{t_1}^{t_2}\left(\int_{s_1}^{t_2} f\left(s_2\right)\mathrm{d}s_2\right)^{\otimes 2}\mathrm{d}s_1$.

Proof. For $f : \mathbf{R}_+ \to \mathbf{R}^d \otimes \mathbf{R}^r$, we set $F(t) = \int_0^t f(s)\,\mathrm{d}s$, and then it follows from Itô's formula that

$$\begin{aligned}
F\left(t\right)w_t&=\int_0^t\left(\frac{\mathrm{d}}{\mathrm{d}t}F\left(t\right)\bigg|_{t=s}\right)w_s\mathrm{d}s+\int_0^t F\left(s\right)\mathrm{d}w_s\\
&=\int_0^t f\left(s\right)w_s\mathrm{d}s+\int_0^t F\left(s\right)\mathrm{d}w_s,
\end{aligned}$$

and

$$\begin{aligned}\int_{t_1}^{t_2} f(s)\left(\int_{t_1}^{s_1} \mathrm{d}w_{s_2}\right)\mathrm{d}s_1 &= \int_{t_1}^{t_2} f(s)\left(w_{s_1}-w_{t_1}\right)\mathrm{d}s_1\\ &= F(t_2)\,w_{t_2} - F(t_1)\,w_{t_1} - \int_{t_1}^{t_2} f(s)\,w_{t_1}\mathrm{d}s - \int_{t_1}^{t_2} F(s)\,\mathrm{d}w_s\\ &= F(t_2)\,w_{t_2} - F(t_1)\,w_{t_1} - \left(F(t_2)-F(t_1)\right)w_{t_1} - \int_{t_1}^{t_2} F(s)\,\mathrm{d}w_s\\ &= \int_{t_1}^{t_2}\left(F(t_2)-F(s)\right)\mathrm{d}w_s\\ &= \int_{t_1}^{t_2}\left(\int_{s_1}^{t_2} f(s_2)\,\mathrm{d}s_2\right)\mathrm{d}w_{s_1},\end{aligned}$$

which is normally distributed because of Wiener integral obviously. □

Lemma 15. *$f_{\mathbb{D}_0}\left(\rho^{(i)},\rho^{(j)}\right)$ is continuous.*

Proof. We check the continuity at (i) $\rho^{(i)}=\rho^{(j)}=0$, (ii) $\rho^{(i)}=0$ and $\rho^{(j)}\in(0,1)$, (iii) $\rho^{(i)}=0$ and $\rho^{(j)}=1$, (iv) $\rho^{(i)}=0$ and $\rho^{(j)}\in(1,\overline{\rho}]$, (v) $\rho^{(j)}=0$ and $\rho^{(i)}\in(0,\overline{\rho}]$, (vi) $\rho^{(i)}=\rho^{(j)}\in(0,1)$, (vii) $\rho^{(i)}=\rho^{(j)}=1$, (viii) $\rho^{(i)}=\rho^{(j)}\in(1,p]$, (ix) $\rho^{(j)}=1$ and $\rho^{(i)}\in(0,1)$, (x) $\rho^{(j)}=1$ and $\rho^{(i)}\in(1,\overline{\rho}]$, (xi) $\rho^{(i)}\in(0,\overline{\rho}]$ and $\rho^{(i)}+1=\rho^{(j)}$.

(i) We have that

$$\begin{aligned}&f_{\mathbb{D}_0}\left(\rho^{(i)},\rho^{(j)}\right)\big|_{\rho^{(i)}=\rho^{(j)}=0}=0,\\ &\lim_{\rho^{(i)}=0,\rho^{(j)}\downarrow 0} f_{\mathbb{D}_0}\left(\rho^{(i)},\rho^{(j)}\right)=\lim_{\rho^{(i)}=0,\rho^{(j)}\downarrow 0}\frac{\rho^{(j)}}{2}=0,\\ &\lim_{\substack{\rho^{(i)}\downarrow,\rho^{(j)}\downarrow 0\\ \rho^{(i)}<\rho^{(j)}}} f_{\mathbb{D}_0}\left(\rho^{(i)},\rho^{(j)}\right)=\lim_{\substack{\rho^{(i)}\downarrow,\rho^{(j)}\downarrow 0\\ \rho^{(i)}<\rho^{(j)}}}\frac{\left(\rho^{(i)}\right)^2-3\rho^{(i)}\rho^{(j)}+3\left(\rho^{(j)}\right)^2}{6\rho^{(j)}}=0,\\ &\lim_{\substack{\rho^{(i)}\downarrow,\rho^{(j)}\downarrow 0\\ \rho^{(i)}\geq\rho^{(j)}}} f_{\mathbb{D}_0}\left(\rho^{(i)},\rho^{(j)}\right)=\lim_{\substack{\rho^{(i)}\downarrow,\rho^{(j)}\downarrow 0\\ \rho^{(i)}\geq\rho^{(j)}}}\frac{\left(\rho^{(j)}\right)^2}{6\rho^{(i)}}=0,\\ &\lim_{\rho^{(j)}=0,\rho^{(i)}\downarrow 0} f_{\mathbb{D}_0}\left(\rho^{(i)},\rho^{(j)}\right)=0.\end{aligned}$$

(ii) It holds that

$$\begin{aligned}&f_{\mathbb{D}_0}\left(\rho^{(i)},\rho^{(j)}\right)\big|_{\rho^{(i)}=0,\rho^{(j)}\in(0,1)}=\frac{\rho^{(j)}}{2},\\ &\lim_{\rho^{(j)}\in(0,1),\rho^{(i)}\downarrow 0} f_{\mathbb{D}_0}\left(\rho^{(i)},\rho^{(j)}\right)=\lim_{\rho^{(j)}\in(0,1),\rho^{(i)}\downarrow 0}\frac{\left(\rho^{(i)}\right)^2-3\rho^{(i)}\rho^{(j)}+3\left(\rho^{(j)}\right)^2}{6\rho^{(j)}}=\frac{\rho^{(j)}}{2}.\end{aligned}$$

(iii) We obtain that

$$
\begin{aligned}
f_{\mathbb{D}_0}\left(\rho^{(i)},\rho^{(j)}\right)\big|_{\rho^{(i)}=0,\rho^{(j)}=1} &= \frac{1}{2},\\
\lim_{\rho^{(i)}=0,\rho^{(j)}\uparrow 1} f_{\mathbb{D}_0}\left(\rho^{(i)},\rho^{(j)}\right) &= \frac{1}{2},\\
\lim_{\rho^{(i)}=0,\rho^{(j)}\downarrow 1} f_{\mathbb{D}_0}\left(\rho^{(i)},\rho^{(j)}\right) &= \lim_{\rho^{(i)}=0,\rho^{(j)}\downarrow 1} \frac{2\rho^{(j)}-1}{2\rho^{(j)}}\\
&= \frac{1}{2},\\
\lim_{\substack{\rho^{(i)}\downarrow 0,\rho^{(j)}\uparrow 1\\ \rho^{(i)}<\rho^{(j)}}} f_{\mathbb{D}_0}\left(\rho^{(i)},\rho^{(j)}\right) &= \lim_{\substack{\rho^{(i)}\downarrow 0,\rho^{(j)}\uparrow 1\\ \rho^{(i)}<\rho^{(j)}}} \frac{\left(\rho^{(i)}\right)^2-3\rho^{(i)}\rho^{(j)}+3\left(\rho^{(j)}\right)^2}{6\rho^{(j)}}\\
&= \frac{1}{2},\\
\lim_{\substack{\rho^{(i)}\downarrow 0,\rho^{(j)}\downarrow 1,\\ \rho^{(i)}<\rho^{(j)}\le\rho^{(i)}+1}} f_{\mathbb{D}_0}\left(\rho^{(i)},\rho^{(j)}\right) &= \lim_{\substack{\rho^{(i)}\downarrow 0,\rho^{(j)}\downarrow 1,\\ \rho^{(i)}<\rho^{(j)}\le\rho^{(i)}+1}} \frac{\left(\rho^{(i)}-\rho^{(j)}\right)^3+3\left(\rho^{(j)}\right)^2-3\rho^{(j)}+1}{6\rho^{(i)}\rho^{(j)}}\\
&= \lim_{\substack{\rho^{(i)}\downarrow 0,\rho^{(j)}\downarrow 1,\\ \rho^{(i)}<\rho^{(j)}\le\rho^{(i)}+1}} \frac{\left(\rho^{(i)}\right)^3-3\left(\rho^{(i)}\right)^2\rho^{(j)}+3\rho^{(i)}\left(\rho^{(j)}\right)^2}{6\rho^{(i)}\rho^{(j)}}\\
&\quad - \lim_{\substack{\rho^{(i)}\downarrow 0,\rho^{(j)}\downarrow 1,\\ \rho^{(i)}<\rho^{(j)}\le\rho^{(i)}+1}} \frac{\left(\rho^{(j)}-1\right)^3}{6\rho^{(i)}\rho^{(j)}}\\
&= \frac{1}{2},\\
\lim_{\substack{\rho^{(i)}\downarrow 0,\rho^{(j)}\downarrow 1,\\ \rho^{(j)}>\rho^{(i)}+1}} f_{\mathbb{D}_0}\left(\rho^{(i)},\rho^{(j)}\right) &= \lim_{\substack{\rho^{(i)}\downarrow 0,\rho^{(j)}\downarrow 1,\\ \rho^{(j)}>\rho^{(i)}+1}} \frac{6\rho^{(i)}\rho^{(j)}-3\left(\rho^{(i)}\right)^2-3\rho^{(i)}}{6\rho^{(i)}\rho^{(j)}}\\
&= \frac{1}{2}.
\end{aligned}
$$

(iv) We can have that

$$
\begin{aligned}
f_{\mathbb{D}_0}\left(\rho^{(i)},\rho^{(j)}\right)\big|_{\rho^{(i)}=0,\rho^{(j)}\in(1,\overline{\rho}]} &= \frac{2\rho^{(j)}-1}{2\rho^{(j)}},\\
\lim_{\rho^{(i)}\downarrow 0,\rho^{(j)}\in(1,\overline{\rho}]} f_{\mathbb{D}_0}\left(\rho^{(i)},\rho^{(j)}\right) &= \lim_{\rho^{(i)}\downarrow 0,\rho^{(j)}\in(1,\overline{\rho}]} \frac{6\rho^{(i)}\rho^{(j)}-3\left(\rho^{(i)}\right)^2-3\rho^{(i)}}{6\rho^{(i)}\rho^{(j)}}\\
&= \frac{2\rho^{(j)}-1}{2\rho^{(j)}}.
\end{aligned}
$$

(v) It holds that

$$
\begin{aligned}
f_{\mathbb{D}_0}\left(\rho^{(i)},\rho^{(j)}\right)\big|_{\rho^{(i)}\in(0,\overline{\rho}],\rho^{(j)}=0} &= 0,\\
\lim_{\substack{\rho^{(i)}\in(0,\overline{\rho}],\rho^{(j)}\downarrow 0\\ \rho^{(i)}\ge\rho^{(j)}}} f_{\mathbb{D}_0}\left(\rho^{(i)},\rho^{(j)}\right) &= \lim_{\substack{\rho^{(i)}\in(0,\overline{\rho}],\rho^{(j)}\downarrow 0\\ \rho^{(i)}\ge\rho^{(j)}}} \frac{\left(\rho^{(j)}\right)^3}{6\rho^{(i)}\rho^{(j)}} = 0.
\end{aligned}
$$

(vi) We have that

$$\begin{aligned}
f_{\mathbb{D}_0}\left(\rho^{(i)},\rho^{(j)}\right)\Big|_{\rho^{(i)}=\rho^{(j)}\in(0,1)} &= \frac{\rho^{(i)}}{6}\\
\lim_{\substack{\rho^{(i)}\in(0,1),\rho^{(i)}-\rho^{(j)}\to 0,\\ \rho^{(i)}\geq\rho^{(j)}}} f_{\mathbb{D}_0}\left(\rho^{(i)},\rho^{(j)}\right) &= \frac{\rho^{(i)}}{6},\\
\lim_{\substack{\rho^{(i)}\in(0,1),\rho^{(i)}-\rho^{(j)}\to 0,\\ \rho^{(i)}<\rho^{(j)}}} f_{\mathbb{D}_0}\left(\rho^{(i)},\rho^{(j)}\right) &= \lim_{\substack{\rho^{(i)}\in(0,1),\rho^{(i)}-\rho^{(j)}\to 0,\\ \rho^{(i)}<\rho^{(j)}}} \frac{\left(\rho^{(i)}-\rho^{(j)}\right)^3+\left(\rho^{(j)}\right)^3}{6\rho^{(i)}\rho^{(j)}} = \frac{\rho^{(i)}}{6}.
\end{aligned}$$

(vii) It holds that

$$\begin{aligned}
f_{\mathbb{D}_0}\left(\rho^{(i)},\rho^{(j)}\right)\Big|_{\rho^{(i)}=\rho^{(j)}=1} &= \frac{1}{6},\\
\lim_{\substack{\rho^{(i)}\to 1,\rho^{(j)}\uparrow 1\\ \rho^{(i)}<\rho^{(j)}}} f_{\mathbb{D}_0}\left(\rho^{(i)},\rho^{(j)}\right) &= \lim_{\substack{\rho^{(i)}\to 1,\rho^{(j)}\uparrow 1\\ \rho^{(i)}<\rho^{(j)}}} \frac{\left(\rho^{(i)}-\rho^{(j)}\right)^3+\left(\rho^{(j)}\right)^3}{6\rho^{(i)}\rho^{(j)}} = \frac{1}{6},\\
\lim_{\substack{\rho^{(i)}\to 1,\rho^{(j)}\uparrow 1\\ \rho^{(i)}\geq\rho^{(j)}}} f_{\mathbb{D}_0}\left(\rho^{(i)},\rho^{(j)}\right) &= \lim_{\substack{\rho^{(i)}\to 1,\rho^{(j)}\uparrow 1\\ \rho^{(i)}\geq\rho^{(j)}}} \frac{\left(\rho^{(j)}\right)^3}{6\rho^{(i)}\rho^{(j)}} = \frac{1}{6},\\
\lim_{\substack{\rho^{(i)}\to 1,\rho^{(j)}\downarrow 1\\ \rho^{(i)}<\rho^{(j)}}} f_{\mathbb{D}_0}\left(\rho^{(i)},\rho^{(j)}\right) &= \lim_{\substack{\rho^{(i)}\to 1,\rho^{(j)}\downarrow 1\\ \rho^{(i)}<\rho^{(j)}}} \frac{\left(\rho^{(i)}-\rho^{(j)}\right)^3+3\left(\rho^{(j)}\right)^2-3\rho^{(j)}+1}{6\rho^{(i)}\rho^{(j)}} = \frac{1}{6},\\
\lim_{\substack{\rho^{(i)}\to 1,\rho^{(j)}\downarrow 1\\ \rho^{(i)}\geq\rho^{(j)}}} f_{\mathbb{D}_0}\left(\rho^{(i)},\rho^{(j)}\right) &= \lim_{\substack{\rho^{(i)}\to 1,\rho^{(j)}\downarrow 1\\ \rho^{(i)}\geq\rho^{(j)}}} \frac{3\left(\rho^{(j)}\right)^2-3\rho^{(j)}+1}{6\rho^{(i)}\rho^{(j)}} = \frac{1}{6}.
\end{aligned}$$

(viii) We have that

$$\begin{aligned}
f_{\mathbb{D}_0}\left(\rho^{(i)},\rho^{(j)}\right)\Big|_{\rho^{(i)}=\rho^{(j)}\in(1,\overline{\rho}]} &= \frac{3\left(\rho^{(i)}\right)^2-3\rho^{(i)}+1}{6\left(\rho^{(i)}\right)^2}\\
\lim_{\substack{\rho^{(i)}\in(1,\overline{\rho}],\rho^{(i)}-\rho^{(j)}\to 0,\\ \rho^{(i)}\geq\rho^{(j)}}} f_{\mathbb{D}_0}\left(\rho^{(i)},\rho^{(j)}\right) &= \frac{3\left(\rho^{(i)}\right)^2-3\rho^{(i)}+1}{6\left(\rho^{(i)}\right)^2},\\
\lim_{\substack{\rho^{(i)}\in(1,\overline{\rho}],\rho^{(i)}-\rho^{(j)}\to 0,\\ \rho^{(i)}<\rho^{(j)}}} f_{\mathbb{D}_0}\left(\rho^{(i)},\rho^{(j)}\right) &= \lim_{\substack{\rho^{(i)}\in(1,\overline{\rho}],\rho^{(i)}-\rho^{(j)}\to 0,\\ \rho^{(i)}<\rho^{(j)}}} \frac{\left(\rho^{(i)}-\rho^{(j)}\right)^3+3\left(\rho^{(j)}\right)^2-3\rho^{(j)}+1}{6\rho^{(i)}\rho^{(j)}}\\
&= \frac{3\left(\rho^{(i)}\right)^2-3\rho^{(i)}+1}{6\left(\rho^{(i)}\right)^2}.
\end{aligned}$$

(ix) It holds that

$$
\begin{aligned}
f_{\mathbb{D}_0}\left(\rho^{(i)},\rho^{(j)}\right)\Big|_{\rho^{(i)}\in(0,1),\rho^{(j)}=1} &= \frac{\left(\rho^{(i)}-1\right)^3+1}{6\rho^{(i)}},\\
\lim_{\rho^{(i)}\in(0,1),\rho^{(j)}\uparrow 1} f_{\mathbb{D}_0}\left(\rho^{(i)},\rho^{(j)}\right) &= \frac{\left(\rho^{(i)}-1\right)^3+1}{6\rho^{(i)}},\\
\lim_{\rho^{(i)}\in(0,1),\rho^{(j)}\downarrow 1} f_{\mathbb{D}_0}\left(\rho^{(i)},\rho^{(j)}\right) &= \lim_{\rho^{(i)}\in(0,1),\rho^{(j)}\downarrow 1} \frac{\left(\rho^{(i)}-\rho^{(j)}\right)^3+3\left(\rho^{(j)}\right)^2-3\rho^{(j)}+1}{6\rho^{(i)}\rho^{(j)}}\\
&= \frac{\left(\rho^{(i)}-1\right)^3+1}{6\rho^{(i)}}.
\end{aligned}
$$

(x) We have that

$$
\begin{aligned}
f_{\mathbb{D}_0}\left(\rho^{(i)},\rho^{(j)}\right)\Big|_{\rho^{(i)}\in(1,\overline{\rho}],\rho^{(j)}=1} &= \frac{1}{6\rho^{(i)}},\\
\lim_{\rho^{(i)}\in(1,\overline{\rho}],\rho^{(j)}\uparrow 1} f_{\mathbb{D}_0}\left(\rho^{(i)},\rho^{(j)}\right) &= \frac{1}{6\rho^{(i)}},\\
\lim_{\rho^{(i)}\in(1,\overline{\rho}],\rho^{(j)}\downarrow 1} f_{\mathbb{D}_0}\left(\rho^{(i)},\rho^{(j)}\right) &= \lim_{\rho^{(i)}\in(1,\overline{\rho}],\rho^{(j)}\downarrow 1} \frac{3\left(\rho^{(j)}\right)^2-3\rho^{(j)}+1}{6\rho^{(i)}\rho^{(j)}}\\
&= \frac{1}{6\rho^{(i)}}.
\end{aligned}
$$

(xi) It holds that

$$
\begin{aligned}
f_{\mathbb{D}_0}\left(\rho^{(i)},\rho^{(j)}\right)\Big|_{\rho^{(i)}\in(0,\overline{\rho}],\rho^{(i)}+1=\rho^{(j)}} &= \frac{1}{2},\\
\lim_{\substack{\rho^{(i)}\in(0,\overline{\rho}],\rho^{(i)}+1-\rho^{(j)}\to 0\\ \rho^{(j)}<\rho^{(i)}+1}} f_{\mathbb{D}_0}\left(\rho^{(i)},\rho^{(j)}\right) &= \frac{1}{2},\\
\lim_{\substack{\rho^{(i)}\in(0,\overline{\rho}],\rho^{(i)}+1-\rho^{(j)}\to 0\\ \rho^{(j)}>\rho^{(i)}+1}} f_{\mathbb{D}_0}\left(\rho^{(i)},\rho^{(j)}\right) &= \lim_{\substack{\rho^{(i)}\in(0,\overline{\rho}],\rho^{(i)}+1-\rho^{(j)}\to 0\\ \rho^{(j)}>\rho^{(i)}+1}} \frac{6\rho^{(i)}\rho^{(j)}-3\left(\rho^{(i)}\right)^2-3\rho^{(i)}}{6\rho^{(i)}\rho^{(j)}}\\
&= \lim_{\substack{\rho^{(i)}\in(0,\overline{\rho}],\rho^{(i)}+1-\rho^{(j)}\to 0\\ \rho^{(j)}>\rho^{(i)}+1}} \frac{6\left(\rho^{(i)}+1\right)-3\rho^{(i)}-3}{6\left(\rho^{(i)}+1\right)}\\
&= \frac{1}{2}.
\end{aligned}
$$

Therefore we obtain the continuity of $f_{\mathbb{D}_0}$. □

Lemma 16. *$f_{\mathbb{G}}\left(\rho^{(i)},\rho^{(j)}\right)$ is continuous.*

Proof. Since the continuity of $f_{\mathbb{D}_0}$ is shown in Lemma 15, it is sufficient to show the continuity of $f_{\mathbb{G}} + f_{\mathbb{D}_0}$. Note that

$$
\begin{aligned}
&\left(f_{\mathbb{G}} + f_{\mathbb{D}_0}\right)\left(\rho^{(i)}, \rho^{(j)}\right) \\
&= \begin{cases}
1 & \text{if } \rho^{(i)} = 0, \\
1 - \frac{\rho^{(i)}}{2} & \text{if } \rho^{(i)} \in (0,1], \rho^{(j)} = 0, \\
\frac{1}{2\rho^{(i)}} & \text{if } \rho^{(i)} \in (1,\overline{\rho}], \rho^{(j)} = 0, \\
\frac{\left(\rho^{(j)}\right)^2 + \rho^{(j)}}{2\rho^{(i)}\rho^{(j)}} & \text{if } \rho^{(i)} \in \left(\rho^{(j)} + 1, \overline{\rho}\right], \rho^{(j)} > 0, \\
\frac{\left(\rho^{(i)} - \rho^{(j)}\right)^3 - 3\left(\rho^{(i)}\right)^2 + 6\rho^{(i)}\rho^{(j)} + 3\rho^{(i)} - 1}{6\rho^{(i)}\rho^{(j)}} & \text{if } \rho^{(i)} \in (1,\overline{\rho}], \rho^{(i)} \in \left(\rho^{(j)}, \rho^{(j)} + 1\right], \\
\frac{-3\left(\rho^{(i)}\right)^2 + 6\rho^{(i)}\rho^{(j)} + 3\rho^{(i)} - 1}{6\rho^{(i)}\rho^{(j)}} & \text{if } \rho^{(i)} \in (1,\overline{\rho}], \rho^{(i)} \le \rho^{(j)}, \\
\frac{-3\left(\rho^{(i)}\right)^2\rho^{(j)} + 3\rho^{(i)}\left(\rho^{(j)}\right)^2 + 6\rho^{(i)}\rho^{(j)} - \left(\rho^{(j)}\right)^3}{6\rho^{(i)}\rho^{(j)}} & \text{if } \rho^{(i)} \in (0,1], \rho^{(i)} > \rho^{(j)}, \\
\frac{6\rho^{(i)}\rho^{(j)} - \left(\rho^{(i)}\right)^3}{6\rho^{(i)}\rho^{(j)}} & \text{if } \rho^{(i)} \in (0,1], \rho^{(i)} \le \rho^{(j)}.
\end{cases}
\end{aligned}
$$

We check the continuity of $f_{\mathbb{G}} + f_{\mathbb{D}_0}$ at (i) $\rho^{(i)} = \rho^{(j)} = 0$, (ii) $\rho^{(i)} \in (0,1)$ and $\rho^{(j)} = 0$, (iii) $\rho^{(i)} = 1$ and $\rho^{(j)} = 0$, (iv) $\rho^{(i)} \in (1,\overline{\rho}]$ and $\rho^{(j)} = 0$, (v) $\rho^{(i)} \in (1,\overline{\rho}]$ and $\rho^{(i)} = \rho^{(j)} + 1$, (vi) $\rho^{(i)} \in (0,1)$ and $\rho^{(i)} = \rho^{(j)}$, (vii) $\rho^{(i)} = 1$ and $\rho^{(j)} \in (0,1)$, (viii) $\rho^{(i)} = \rho^{(j)} = 1$, (ix) $\rho^{(i)} = 1$ and $\rho^{(j)} \in (1,\overline{\rho}]$, (x) $\rho^{(i)} \in (1,\overline{\rho}]$ and $\rho^{(i)} = \rho^{(j)}$, (xi) $\rho^{(i)} = 0$, $\rho^{(j)} \in (0,\overline{\rho}]$.
For (i), we obtain that

$$
\begin{aligned}
&\left(f_{\mathbb{G}} + f_{\mathbb{D}_0}\right)\left(\rho^{(i)}, \rho^{(j)}\right)\Big|_{\rho^{(i)}=\rho^{(j)}=0} = 1, \\
&\lim_{\rho^{(i)}\downarrow 0, \rho^{(j)}=0} \left(f_{\mathbb{G}} + f_{\mathbb{D}_0}\right)\left(\rho^{(i)}, \rho^{(j)}\right) = \lim_{\rho^{(i)}\downarrow 0, \rho^{(j)}=0} \left(1 - \frac{\rho^{(i)}}{2}\right) = 1, \\
&\lim_{\substack{\rho^{(i)}\downarrow 0, \rho^{(j)}\downarrow 0, \\ \rho^{(i)} > \rho^{(j)}}} \left(f_{\mathbb{G}} + f_{\mathbb{D}_0}\right)\left(\rho^{(i)}, \rho^{(j)}\right) = \lim_{\substack{\rho^{(i)}\downarrow 0, \rho^{(j)}\downarrow 0, \\ \rho^{(i)} > \rho^{(j)}}} \frac{3\rho^{(i)}\left(\rho^{(j)}\right)^2 - 3\left(\rho^{(i)}\right)^2\rho^{(j)} + 6\rho^{(i)}\rho^{(j)} - \left(\rho^{(j)}\right)^3}{6\rho^{(i)}\rho^{(j)}} \\
&\qquad = 1, \\
&\lim_{\substack{\rho^{(i)}\downarrow 0, \rho^{(j)}\downarrow 0, \\ \rho^{(i)} \le \rho^{(j)}}} \left(f_{\mathbb{G}} + f_{\mathbb{D}_0}\right)\left(\rho^{(i)}, \rho^{(j)}\right) = \lim_{\substack{\rho^{(i)}\downarrow 0, \rho^{(j)}\downarrow 0, \\ \rho^{(i)} \le \rho^{(j)}}} \frac{6\rho^{(i)}\rho^{(j)} - \left(\rho^{(i)}\right)^3}{6\rho^{(i)}\rho^{(j)}} = 1, \\
&\lim_{\rho^{(i)}=0, \rho^{(j)}\downarrow 0} \left(f_{\mathbb{G}} + f_{\mathbb{D}_0}\right)\left(\rho^{(i)}, \rho^{(j)}\right) = \lim_{\rho^{(i)}=0, \rho^{(j)}\downarrow 0} 1 = 1.
\end{aligned}
$$

For (ii), one has that

$$
\begin{aligned}
&\left(f_{\mathbb{G}} + f_{\mathbb{D}_0}\right)\left(\rho^{(i)}, \rho^{(j)}\right)\Big|_{\rho^{(i)}\in(0,1), \rho^{(j)}=0} = 1 - \frac{\rho^{(i)}}{2}, \\
&\lim_{\rho^{(i)}\in(0,1), \rho^{(j)}\downarrow 0} \left(f_{\mathbb{G}} + f_{\mathbb{D}_0}\right)\left(\rho^{(i)}, \rho^{(j)}\right) \\
&\quad = \lim_{\rho^{(i)}\in(0,1), \rho^{(j)}\downarrow 0} \frac{-3\left(\rho^{(i)}\right)^2\rho^{(j)} + 3\rho^{(i)}\left(\rho^{(j)}\right)^2 + 6\rho^{(i)}\rho^{(j)} - \left(\rho^{(j)}\right)^3}{6\rho^{(i)}\rho^{(j)}} \\
&\quad = 1 - \frac{\rho^{(i)}}{2}.
\end{aligned}
$$

For (iii), we can evaluate that

$$
\begin{aligned}
&\left(f_{\mathbb{G}}+f_{\mathbb{D}_0}\right)\left(\rho^{(i)},\rho^{(j)}\right)\big|_{\rho^{(i)}=1,\rho^{(j)}=0}=\frac{1}{2},\\
&\lim_{\rho^{(i)}\uparrow 1,\rho^{(j)}=0}\left(f_{\mathbb{G}}+f_{\mathbb{D}_0}\right)\left(\rho^{(i)},\rho^{(j)}\right)=\lim_{\rho^{(i)}\uparrow 1,\rho^{(j)}=0}\left(1-\frac{\rho^{(i)}}{2}\right)=\frac{1}{2},\\
&\lim_{\substack{\rho^{(i)}\to 1,\rho^{(j)}\to 0\\ \rho^{(i)}\in(0,1],\rho^{(i)}>\rho^{(j)}}}\left(f_{\mathbb{G}}+f_{\mathbb{D}_0}\right)\left(\rho^{(i)},\rho^{(j)}\right)\\
&=\lim_{\substack{\rho^{(i)}\to 1,\rho^{(j)}\to 0\\ \rho^{(i)}\in(0,1],\rho^{(i)}>\rho^{(j)}}}\frac{3\rho^{(i)}\left(\rho^{(j)}\right)^2-3\left(\rho^{(i)}\right)^2\rho^{(j)}+6\rho^{(i)}\rho^{(j)}-\left(\rho^{(j)}\right)^3}{6\rho^{(i)}\rho^{(j)}}=\frac{1}{2},\\
&\lim_{\substack{\rho^{(i)}\to 1,\rho^{(j)}\to 0\\ \rho^{(i)}\in(1,\overline{\rho}],\rho^{(i)}\in\left(\rho^{(j)},\rho^{(j)}+1\right]}}\left(f_{\mathbb{G}}+f_{\mathbb{D}_0}\right)\left(\rho^{(i)},\rho^{(j)}\right)\\
&=\lim_{\substack{\rho^{(i)}\to 1,\rho^{(j)}\to 0\\ \rho^{(i)}\in(1,\overline{\rho}],\rho^{(i)}\in\left(\rho^{(j)},\rho^{(j)}+1\right]}}\frac{\left(\rho^{(i)}-\rho^{(j)}\right)^3-3\left(\rho^{(i)}\right)^2+6\rho^{(i)}\rho^{(j)}+3\rho^{(i)}-1}{6\rho^{(i)}\rho^{(j)}}\\
&=\lim_{\substack{\rho^{(i)}\to 1,\rho^{(j)}\to 0\\ \rho^{(i)}\in(1,\overline{\rho}],\rho^{(i)}\in\left(\rho^{(j)},\rho^{(j)}+1\right]}}\frac{\left(\rho^{(i)}\right)^3-3\left(\rho^{(i)}\right)^2\rho^{(j)}-3\left(\rho^{(i)}\right)^2+3\rho^{(i)}-1}{6\rho^{(i)}\rho^{(j)}}+1\\
&=\lim_{\substack{\rho^{(i)}\to 1,\rho^{(j)}\to 0\\ \rho^{(i)}\in(1,\overline{\rho}],\rho^{(i)}\in\left(\rho^{(j)},\rho^{(j)}+1\right]}}\frac{\left(\rho^{(i)}\right)^3-3\left(\rho^{(i)}\right)^2+3\rho^{(i)}-1}{6\rho^{(i)}\rho^{(j)}}+\frac{1}{2}\\
&=\lim_{\substack{\rho^{(i)}\to 1,\rho^{(j)}\to 0\\ \rho^{(i)}\in(1,\overline{\rho}],\rho^{(i)}\in\left(\rho^{(j)},\rho^{(j)}+1\right]}}\frac{\left(\rho^{(i)}-1\right)^3}{6\rho^{(i)}\rho^{(j)}}+\frac{1}{2}\\
&=\frac{1}{2},\\
&\lim_{\substack{\rho^{(i)}\to 1,\rho^{(j)}\to 0\\ \rho^{(i)}\in(1,\overline{\rho}],\rho^{(i)}>\rho^{(j)}+1}}\left(f_{\mathbb{G}}+f_{\mathbb{D}_0}\right)\left(\rho^{(i)},\rho^{(j)}\right)=\lim_{\substack{\rho^{(i)}\to 1,\rho^{(j)}\to 0\\ \rho^{(i)}\in(1,\overline{\rho}],\rho^{(i)}>\rho^{(j)}+1}}\frac{\left(\rho^{(j)}\right)^2+\rho^{(j)}}{2\rho^{(i)}\rho^{(j)}}=\frac{1}{2},\\
&\lim_{\rho^{(i)}\downarrow 1,\rho^{(j)}=0}\left(f_{\mathbb{G}}+f_{\mathbb{D}_0}\right)\left(\rho^{(i)},\rho^{(j)}\right)=\lim_{\rho^{(i)}\downarrow 1,\rho^{(j)}=0}\frac{1}{2\rho^{(i)}}=\frac{1}{2}.
\end{aligned}
$$

For (iv), we obtain that

$$
\begin{aligned}
&\left(f_{\mathbb{G}}+f_{\mathbb{D}_0}\right)\left(\rho^{(i)},\rho^{(j)}\right)\big|_{\rho^{(i)}=1,\rho^{(j)}=0}=\frac{1}{2\rho^{(i)}},\\
&\lim_{\rho^{(i)}\in(1,\overline{\rho}],\rho^{(j)}\downarrow 0}\left(f_{\mathbb{G}}+f_{\mathbb{D}_0}\right)\left(\rho^{(i)},\rho^{(j)}\right)=\lim_{\rho^{(i)}\in(1,\overline{\rho}],\rho^{(j)}\downarrow 0}\frac{\left(\rho^{(j)}\right)^2+\rho^{(j)}}{2\rho^{(i)}\rho^{(j)}}=\frac{1}{2\rho^{(i)}}.
\end{aligned}
$$

For (v), it holds that

$$
\left(f_{\mathbb{G}}+f_{\mathbb{D}_0}\right)\left(\rho^{(i)},\rho^{(j)}\right)\big|_{\rho^{(i)}\in(1,\overline{\rho}],\rho^{(i)}=\rho^{(j)}+1}=\frac{1}{2},
$$

$$\lim_{\substack{\rho^{(i)}\in(1,\overline{\rho}],\rho^{(j)}-\rho^{(i)}+1\to 0\\ \rho^{(j)}>\rho^{(i)}-1}} \left(f_{\mathbb{G}}+f_{\mathbb{D}_0}\right)\left(\rho^{(i)},\rho^{(j)}\right)=\frac{1}{2},$$

$$\lim_{\substack{\rho^{(i)}\in(1,\overline{\rho}],\rho^{(j)}-\rho^{(i)}+1\to 0\\ \rho^{(j)}\le\rho^{(i)}-1}} \left(f_{\mathbb{G}}+f_{\mathbb{D}_0}\right)\left(\rho^{(i)},\rho^{(j)}\right)=\lim_{\substack{\rho^{(i)}\in(1,\overline{\rho}],\rho^{(j)}-\rho^{(i)}+1\to 0\\ \rho^{(j)}\le\rho^{(i)}-1}} \frac{\left(\rho^{(j)}\right)^2+\rho^{(j)}}{2\rho^{(i)}\rho^{(j)}}=\frac{1}{2}.$$

For (vi), we have that

$$\begin{aligned}
&\left(f_{\mathbb{G}}+f_{\mathbb{D}_0}\right)\left(\rho^{(i)},\rho^{(j)}\right)\Big|_{\rho^{(i)}\in(0,1),\rho^{(i)}=\rho^{(j)}}=1-\frac{\rho^{(i)}}{6},\\
&\lim_{\substack{\rho^{(i)}\in(0,1),\rho^{(i)}-\rho^{(j)}\to 0\\ \rho^{(i)}\le\rho^{(j)}}} \left(f_{\mathbb{G}}+f_{\mathbb{D}_0}\right)\left(\rho^{(i)},\rho^{(j)}\right)=1-\frac{\rho^{(i)}}{6},\\
&\lim_{\substack{\rho^{(i)}\in(0,1),\rho^{(i)}-\rho^{(j)}\to 0\\ \rho^{(i)}>\rho^{(j)}}} \left(f_{\mathbb{G}}+f_{\mathbb{D}_0}\right)\left(\rho^{(i)},\rho^{(j)}\right)\\
&=\lim_{\substack{\rho^{(i)}\in(0,1),\rho^{(i)}-\rho^{(j)}\to 0\\ \rho^{(i)}>\rho^{(j)}}} \frac{-3\left(\rho^{(i)}\right)^2\rho^{(j)}+3\rho^{(i)}\left(\rho^{(j)}\right)^2+6\rho^{(i)}\rho^{(j)}-\left(\rho^{(j)}\right)^3}{6\rho^{(i)}\rho^{(j)}}\\
&=1-\frac{\rho^{(i)}}{6}.
\end{aligned}$$

For (vii), it holds that

$$\begin{aligned}
&\left(f_{\mathbb{G}}+f_{\mathbb{D}_0}\right)\left(\rho^{(i)},\rho^{(j)}\right)\Big|_{\rho^{(i)}=1,\rho^{(j)}\in(0,1)}=\frac{1}{2}+\frac{\rho^{(j)}}{2}-\frac{\left(\rho^{(j)}\right)^2}{6},\\
&\lim_{\substack{\rho^{(i)}\to 1,\rho^{(j)}\in(0,1)\\ \rho^{(i)}>\rho^{(j)}}} \left(f_{\mathbb{G}}+f_{\mathbb{D}_0}\right)\left(\rho^{(i)},\rho^{(j)}\right)=\frac{1}{2}+\frac{\rho^{(j)}}{2}-\frac{\left(\rho^{(j)}\right)^2}{6},\\
&\lim_{\substack{\rho^{(i)}\to 1,\rho^{(j)}\in(0,1)\\ \rho^{(i)}\in\left(\rho^{(j)},\rho^{(j)}+1\right]}} \left(f_{\mathbb{G}}+f_{\mathbb{D}_0}\right)\left(\rho^{(i)},\rho^{(j)}\right)\\
&\quad=\lim_{\substack{\rho^{(i)}\to 1,\rho^{(j)}\in(0,1)\\ \rho^{(i)}\in\left(\rho^{(j)},\rho^{(j)}+1\right]}} \frac{\left(\rho^{(i)}-\rho^{(j)}\right)^3-3\left(\rho^{(i)}\right)^2+6\rho^{(i)}\rho^{(j)}+3\rho^{(i)}-1}{6\rho^{(i)}\rho^{(j)}}\\
&\quad=\lim_{\substack{\rho^{(i)}\to 1,\rho^{(j)}\in(0,1)\\ \rho^{(i)}\in\left(\rho^{(j)},\rho^{(j)}+1\right]}} \frac{\left(\rho^{(i)}-\rho^{(j)}\right)^3-1}{6\rho^{(i)}\rho^{(j)}}+1\\
&\quad=\lim_{\substack{\rho^{(i)}\to 1,\rho^{(j)}\in(0,1)\\ \rho^{(i)}\in\left(\rho^{(j)},\rho^{(j)}+1\right]}} \frac{\left(\rho^{(i)}\right)^3-3\left(\rho^{(i)}\right)^2\rho^{(j)}+3\rho^{(i)}\left(\rho^{(j)}\right)^2-\left(\rho^{(j)}\right)^3-1}{6\rho^{(i)}\rho^{(j)}}+1\\
&=\frac{1}{2}+\frac{\rho^{(j)}}{2}-\frac{\left(\rho^{(j)}\right)^2}{6}.
\end{aligned}$$

For (viii), we have that

$$\begin{aligned}
\left(f_{\mathbb{G}}+f_{\mathbb{D}_0}\right)\left(\rho^{(i)},\rho^{(j)}\right)\big|_{\rho^{(i)}=\rho^{(j)}=1} &= \frac{5}{6},\\
\lim_{\substack{\rho^{(i)}\to 1,\rho^{(j)}\to 1\\ \rho^{(i)}\in(0,1),\rho^{(i)}\le\rho^{(j)}}} \left(f_{\mathbb{G}}+f_{\mathbb{D}_0}\right)\left(\rho^{(i)},\rho^{(j)}\right) &= \frac{5}{6},\\
\lim_{\substack{\rho^{(i)}\to 1,\rho^{(j)}\to 1\\ \rho^{(i)}\in(0,1),\rho^{(i)}>\rho^{(j)}}} \left(f_{\mathbb{G}}+f_{\mathbb{D}_0}\right)\left(\rho^{(i)},\rho^{(j)}\right) &= \frac{5}{6},\\
\lim_{\substack{\rho^{(i)}\to 1,\rho^{(j)}\to 1\\ \rho^{(i)}\in(1,\overline{\rho}],\rho^{(i)}\in\left(\rho^{(j)},\rho^{(j)}+1\right]}} \left(f_{\mathbb{G}}+f_{\mathbb{D}_0}\right)\left(\rho^{(i)},\rho^{(j)}\right) &= \frac{5}{6},\\
\lim_{\substack{\rho^{(i)}\to 1,\rho^{(j)}\to 1\\ \rho^{(i)}\in(1,\overline{\rho}],\rho^{(i)}\le\rho^{(j)}}} \left(f_{\mathbb{G}}+f_{\mathbb{D}_0}\right)\left(\rho^{(i)},\rho^{(j)}\right) &= \frac{5}{6}
\end{aligned}$$

For (ix), we obtain that

$$\begin{aligned}
\left(f_{\mathbb{G}}+f_{\mathbb{D}_0}\right)\left(\rho^{(i)},\rho^{(j)}\right)\big|_{\rho^{(i)}=1,\rho^{(j)}\in(1,\overline{\rho}]} &= 1-\frac{1}{6\rho^{(j)}},\\
\lim_{\substack{\rho^{(i)}\to 1,\rho^{(j)}\in(1,\overline{\rho}]\\ \rho^{(i)}\in(0,1)}} \left(f_{\mathbb{G}}+f_{\mathbb{D}_0}\right)\left(\rho^{(i)},\rho^{(j)}\right) &= 1-\frac{1}{6\rho^{(j)}},\\
\lim_{\substack{\rho^{(i)}\to 1,\rho^{(j)}\in(1,\overline{\rho}]\\ \rho^{(i)}\in(1,\overline{\rho}]}} \left(f_{\mathbb{G}}+f_{\mathbb{D}_0}\right)\left(\rho^{(i)},\rho^{(j)}\right) &= \lim_{\substack{\rho^{(i)}\to 1,\rho^{(j)}\in(1,\overline{\rho}]\\ \rho^{(i)}\in(1,\overline{\rho}]}} \frac{-3\left(\rho^{(i)}\right)^2+6\rho^{(i)}\rho^{(j)}+3\rho^{(i)}-1}{6\rho^{(i)}\rho^{(j)}}\\
&= 1-\frac{1}{6\rho^{(j)}}.
\end{aligned}$$

For (x), it holds that

$$\begin{aligned}
\left(f_{\mathbb{G}}+f_{\mathbb{D}_0}\right)\left(\rho^{(i)},\rho^{(j)}\right)\big|_{\rho^{(i)}=\rho^{(j)}\in(1,\overline{\rho}]} &= \frac{3\left(\rho^{(i)}\right)^2+3\rho^{(i)}-1}{6\left(\rho^{(i)}\right)^2},\\
\lim_{\substack{\rho^{(i)}-\rho^{(j)}\to 0\\ \rho^{(i)}\le\rho^{(j)}}} \left(f_{\mathbb{G}}+f_{\mathbb{D}_0}\right)\left(\rho^{(i)},\rho^{(j)}\right) &= \frac{3\left(\rho^{(i)}\right)^2+3\rho^{(i)}-1}{6\left(\rho^{(i)}\right)^2},\\
\lim_{\substack{\rho^{(i)}-\rho^{(j)}\to 0\\ \rho^{(i)}>\rho^{(j)}}} \left(f_{\mathbb{G}}+f_{\mathbb{D}_0}\right)\left(\rho^{(i)},\rho^{(j)}\right) &= \frac{3\left(\rho^{(i)}\right)^2+3\rho^{(i)}-1}{6\left(\rho^{(i)}\right)^2}.
\end{aligned}$$

For (xi), we obtain that

$$\begin{aligned}
\left(f_{\mathbb{G}}+f_{\mathbb{D}_0}\right)\left(\rho^{(i)},\rho^{(j)}\right)\big|_{\rho^{(i)}=0,\rho^{(j)}\in(0,\overline{\rho}]} &= 1,\\
\lim_{\rho^{(i)}\downarrow 0,\rho^{(j)}\in(0,\overline{\rho}]} \left(f_{\mathbb{G}}+f_{\mathbb{D}_0}\right)\left(\rho^{(i)},\rho^{(j)}\right) &= 1.
\end{aligned}$$

Hence we have the continuity of $f_{\mathbb{G}}$. □

References

Genon-Catalot, V. and Jacod, J. (1993). On the estimation of the diffusion coefficient for multidimensional diffusion processes. *Annales de l'Institut Henri Poincaré Probabilités et statistiques*, 29:119–151.

Gloter, A. (2000). Discrete sampling of an integrated diffusion process and parameter estimation of the diffusion coefficient. *ESAIM: Probability and Statistics*, 4:205–227.

Gloter, A. (2006). Parameter estimation for a discretely observed integrated diffusion process. *Scandinavian Journal of Statistics*, 33(1):83–104.

Ibragimov, I. A. and Has'minskii, R. Z. (1981). *Statistical estimation.* Springer Verlag, New York.

Kessler, M. (1997). Estimation of an ergodic diffusion from discrete observations. *Scandinavian Journal of Statistics*, 24:211–229.

Nakakita, S. H. and Uchida, M. (2019). Inference for ergodic diffusions plus noise. *Scandinavian Journal of Statistics*, 46(2):470–516.
